# Supplementary material for: Disruption of pancreatic stellate cell myofibroblast phenotype promotes pancreatic tumor invasion
Source: Cell Rep. 2022 Jan 25;38(4):110227. doi: 10.1016/j.celrep.2021.110227 (PMC8810397; doi:10.1016/j.celrep.2021.110227)
Supplement: Document S1. Figures S1–S6 and Tables S3, S5, and S6 [file mmc1.pdf]

Cell Reports, Volume *38*

Supplemental information

Disruption of pancreatic stellate cell myofibroblast phenotype promotes pancreatic tumor invasion

Elizabeth R. Murray, Shinelle Menezes, Jack C. Henry, Josie L. Williams, Lorena Alba-Castellón, Priththivika Baskaran, Ivan Quétier, Ami Desai, Jacqueline J.T. Marshall, Ian Rosewell, Marianthi Tatari, Vinothini Rajeeve, Faraz Khan, Jun Wang, Panoraia Kotantaki, Eleanor J. Tyler, Namrata Singh, Claire S. Reader, Edward P. Carter, Kairbaan Hodivala-Dilke, Richard P. Grose, Hemant M. Kocher, Nuria Gavara, Oliver Pearce, Pedro Cutillas, John F. Marshall, and Angus J.M. Cameron

Figure S1

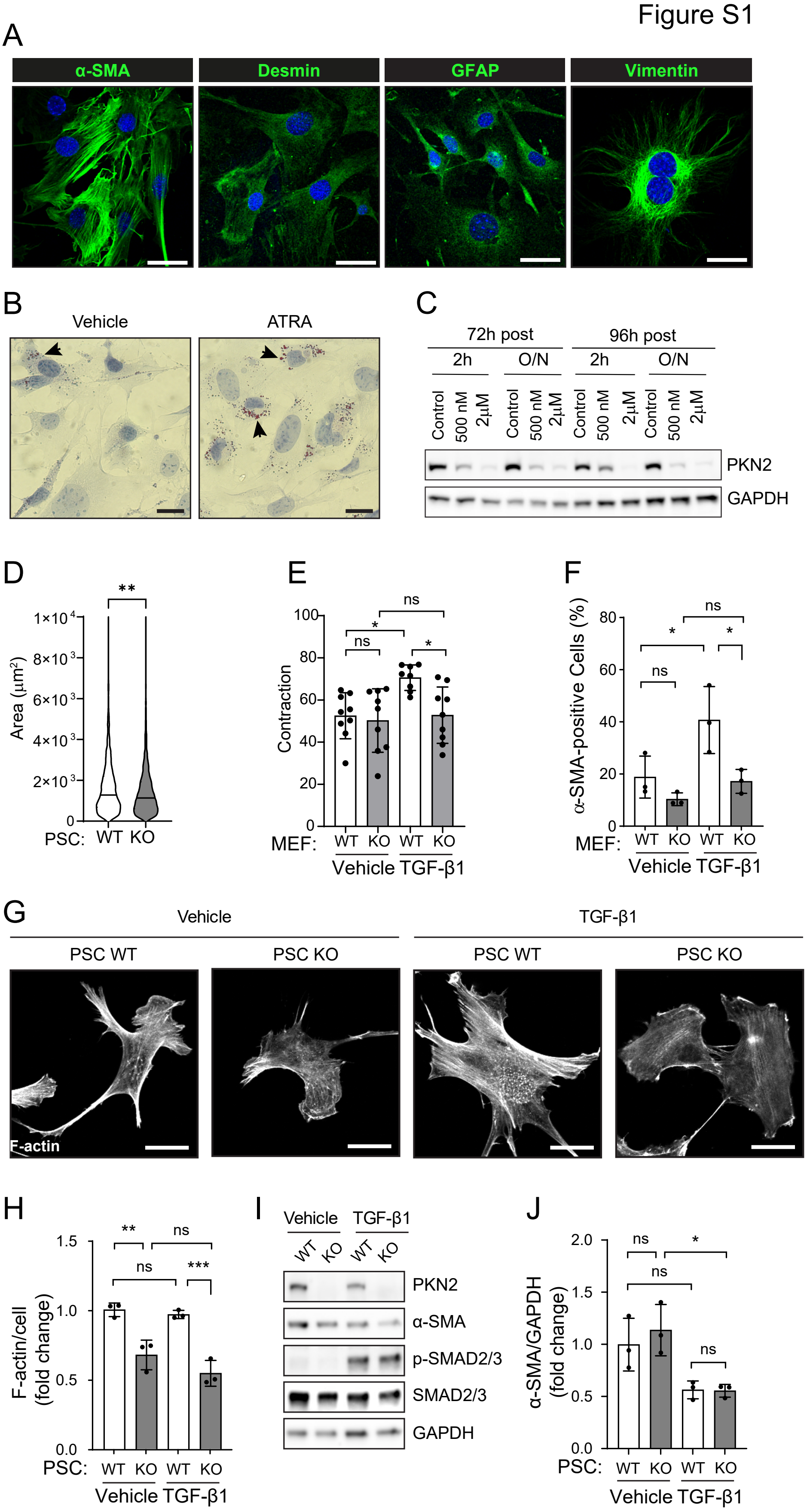

Supplemental Figure 1 (Related to Figure 1). Characterisation of inducible PKN2^{KO} PSCs and MEFs. (A) Immunofluorescence images of α-SMA, Desmin, GFAP and Vimentin staining of PSCs plated on glass coverslips. Scale bar=50 μm. (B) Oil Red O staining of PSCs plated on glass coverslips and treated with vehicle or ATRA daily for 4 days. Scale bar=25 μm. (C) Western Blot analysis of PKN2 deletion *in vitro* in PSCs for indicated time points of incubation and concentration of 4-hydroxy tamoxifen. (D) Quantification of area of WT and PKN2^{KO} PSCs as determined from phalloidin stains imaged by confocal microscopy. (E) Quantification of collagen gel contraction of WT and PKN2^{KO} MEFs treated with 5 ng/ml TGF-β1 or vehicle for 72 h expressed using the formula (1- ratio of gel size/well size)*100. Data are relative to WT MEFs treated with vehicle (n=3, two-way ANOVA with Tukey's multiple comparisons test). (F) Percentage of α-SMA-positive cells in WT or PKN2KO MEFs treated with vehicle or 5 ng/ml TGF-β1 for 72 h (n=3, two-way ANOVA with Tukey's multiple comparisons test). (G) F-actin staining in WT and PKN2^{KO} PSCs treated with vehicle or 5 ng/ml TGF-β1 for 72 h. Scale bar = 25 μm. (H) Number of F-actin fibres per cell treated with vehicle or 5 ng/ml TGF-β1 for 72 h relative to WT PSCs treated with vehicle, quantified by MATLAB algorithm (n=3, >30 cells/condition/experiment). (I) Total α-SMA expression in WT and PKN2^{KO} PSCs treated with vehicle or 5 ng/ml TGF-β1 for 72 h (representative of n=3). (J) Quantification of total α-SMA/GAPDH in WT and KO PSCs treated with vehicle or 5 ng/ml TGF-β1 for 72 h (n=3, two-way ANOVA with Tukey's multiple comparisons test); *p<0.05**p<0.01, ***p<0.001.

Figure S2

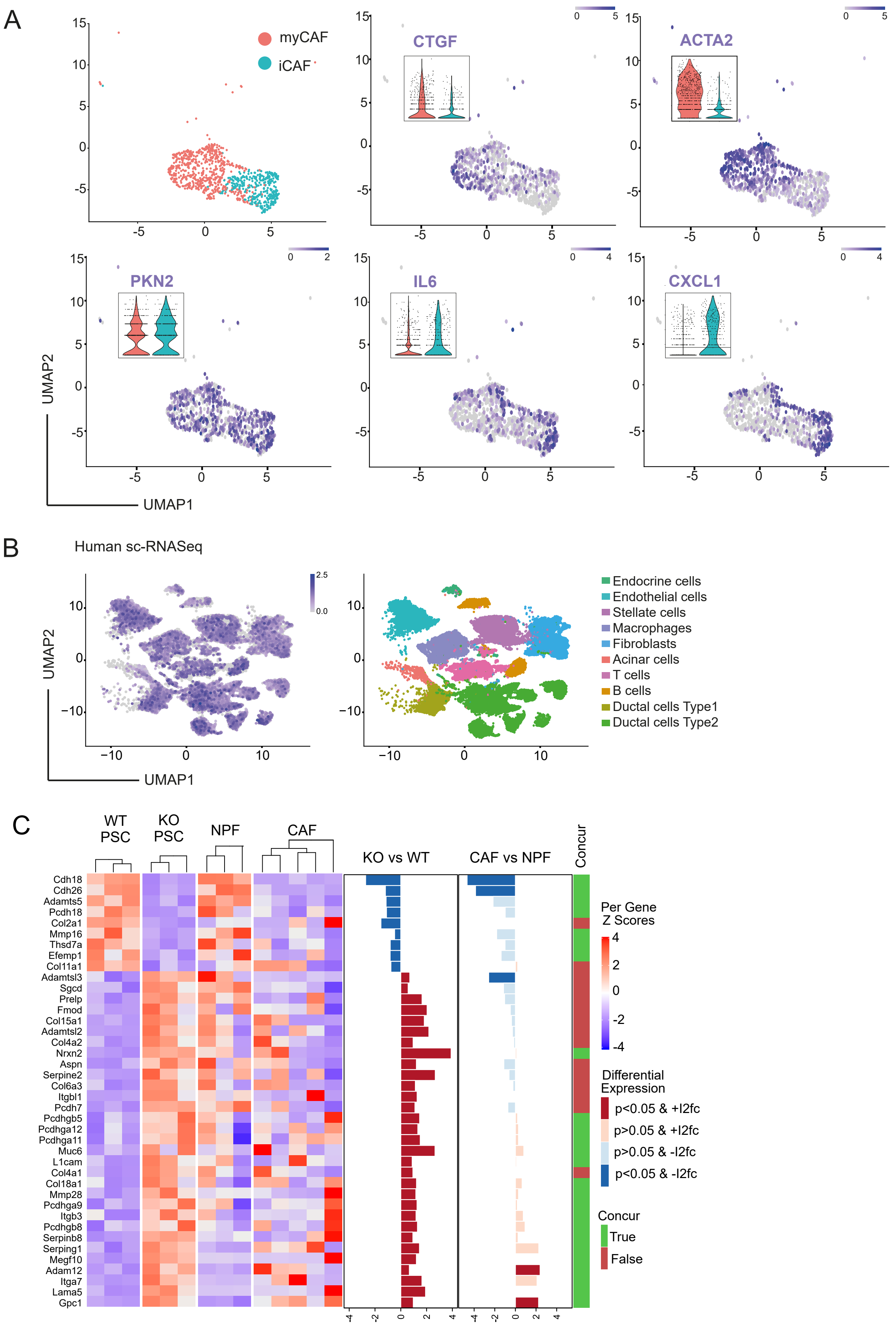

Supplemental Figure 2 (Related to Figure 2). PKN2-deleted PSCs show a distinct matrisome expression profile to normal pancreatic fibroblasts. (A) UMAP plots of single cell RNA-seq data from Biffi et al, 2019 resolving fibroblasts into iCAFs and myCAFs based on iCAF markers Il6, Lif and Cxcl1 and myCAF markers Acta2, Ctgf.; expression level of indicated genes among single cells overlayed in purple on UMAPs. Insets show violin plots of expression levels of indicated genes in iCAFs (pink) and myCAFs (blue). (B) UMAP analyses of single cell RNA seq data from Peng et al, 2019 of various tumour microenvironment cell populations overlayed with their expression level of PKN2 (purple). (C) The expression of ECM panel transcripts was compared between our WT and PKN2KO PSCs and mouse orthotopic pancreatic tumour CAF expression data from Djurek et al, using the panel of DE ECM genes. Concurrence of changes between the two datasets is indicated in the right-hand side bar (concur).

Figure S3

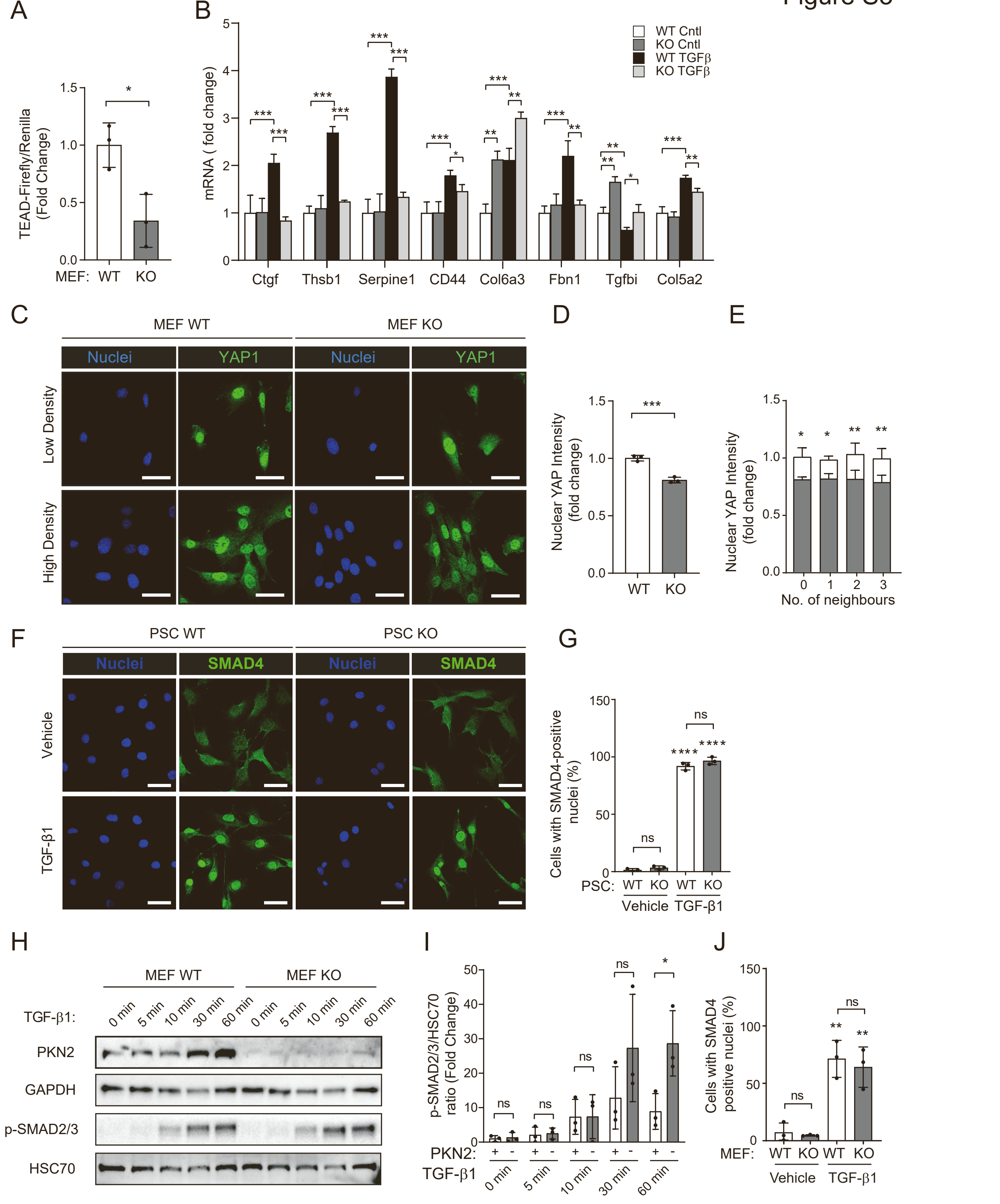

Supplemental Figure 3 (Related to Figure 3). Characterisation of YAP and TGF-β1 signalling in PKN2^{KO} MEFs and PSCs. (A) Normalised expression of TEAD Firefly luciferase reporter relative to the expression of a Renilla luciferase control reporter in WT and KO MEFs grown in 10% serum (unpaired t-test from triplicate transfections; n=2). (B) mRNA expression analysis of indicated genes from targeted RNA-seq data of WT and PKN2^{KO} PSCs treated with either control or TGFβ1 and expressed relative to WT control (n=4; ratio paired t-test). (C) Representative confocal microscopy images of YAP1 localisation (green) in WT and PKN2^{KO} MEFs plated at high or low density on glass coverslips for 48hrs; (minimum of 200 cells/ condition, n=3; scale bar=50 μm). (D) Quantification by Python algorithm of YAP nuclear intensity of all WT and PKN2^{KO} MEFs plated relative to WT (n=3, unpaired t-test). (E) Quantification by Python CellProfiler algorithm of YAP nuclear intensity grouped by number of cell neighbours in WT (white) and PKN2^{KO} (grey) MEFs relative to WT MEFs with zero neighbours.; n=3, two-way ANOVA with Sidak's multiple comparisons test. (F, G) Representative images (F) and quantification (G) (percentage of positive cells) of nuclear SMAD4 (green) in serum-starved or PSCs treated with 5 ng/ml TGF-β1 for 2h (Scale bar = 50 μm, n=3 , two-way ANOVA with Tukey's multiple comparisons test. (H) p-SMAD2/3 induction in WT and PKN2^{KO} MEFs treated with 5 ng/ml TGF-β1 for 0, 5, 10, 30 or 60 mins (representative of n=3). (I) Quantification of pSMAD2/3 induction relative to HSC70 (expressed relative to untreated WT MEFs); n=3, two-way ANOVA with Sidak's multiple comparison's test. (J) Percentage of WT and PKN2^{KO} MEFs with SMAD4-positive nuclei when serum-starved and treated with vehicle or 5 ng/ml TGF-β1 for 2 h; n=3, two-way ANOVA with Tukey's multiple comparisons test. *p<0.05, **p<0.01, ***p<0.001.

Figure S4

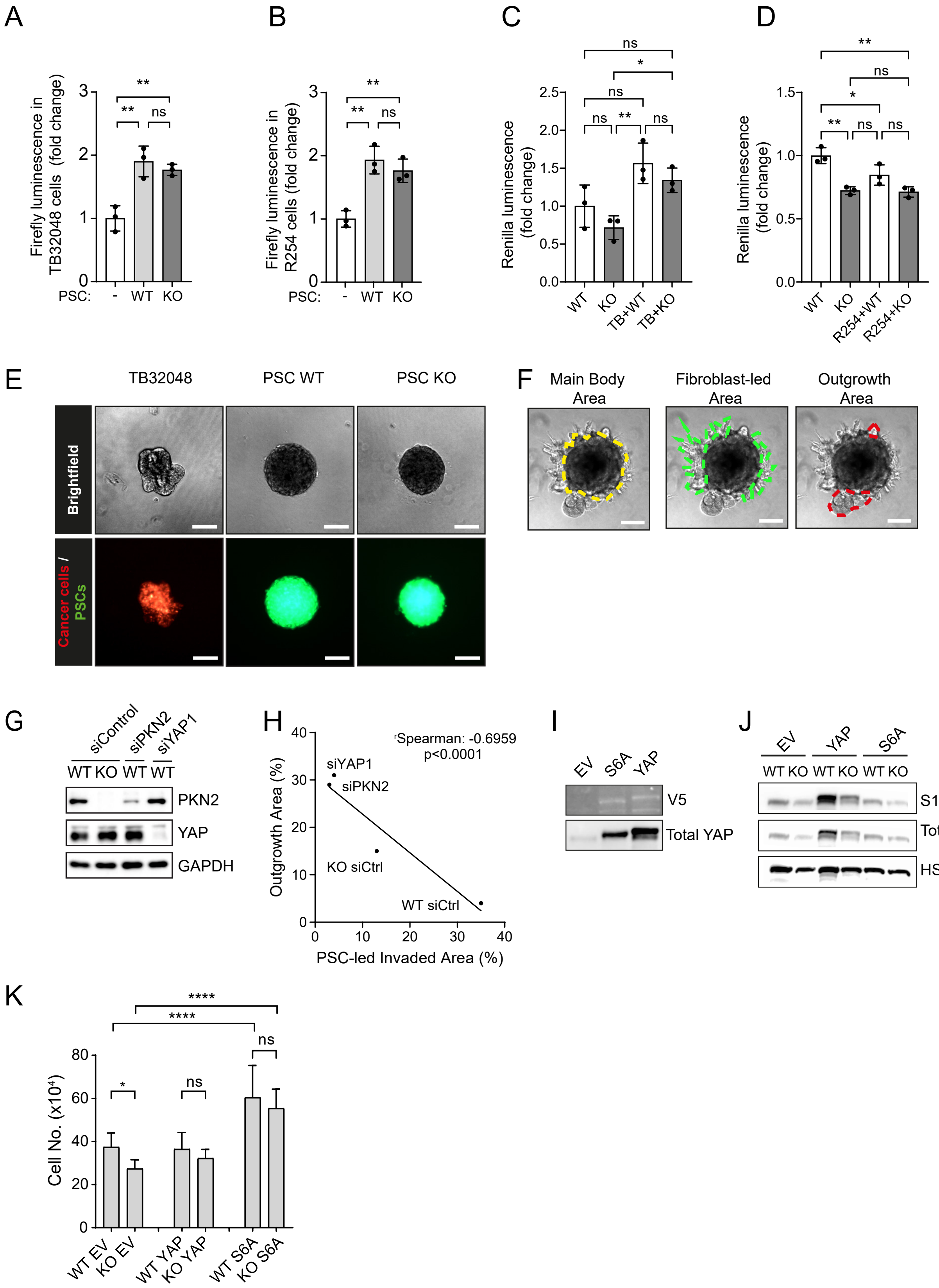

Supplemental Figure 4 (Related to Figure 4). Impact of PKN2 loss on PSC induced growth and invasion. (A, B) Firefly luminescence of TB32048 cells or R254 cells cultured alone or with WT or PKN2^{KO} PSCs in 0.5% serum for 3 days expressed as fold change relative to cells cultured alone (n=3, one-way ANOVA with Tukey's multiple comparisons test). (C,D) Renilla luminescence of WT or PKN2^{KO} PSCs cultured with (C) TB32048 cells or (D) R254 cells in 0.5% serum for 3 days expressed as fold change to WT PSCs cultured alone (n=3, one-way ANOVA with Tukey's multiple comparisons test). (E) Brightfield (top) and epifluorescence images (bottom) of spheroids containing H2B-RFP TB32048 PDAC cells (red), or H2B-GFP WT or PKN2^{KO} PSCs alone (green) embedded in Matrigel matrix for 3 days (n>5). (F) Overlays illustrating definition of spheroid body area (yellow), fibroblast-led invaded area (green) and cancer cell outgrowth area (red) based on cell morphology under brightfield microscope. (G) Western blot analysis of PKN2 and YAP expression in PSCs treated with siRNA against PKN2 or YAP. (H) Correlation of the percentages of PSC-led invaded area and cancer cell outgrowth area relative to total area of each spheroid. (I) Western blot of V5 tag and total YAP in PSCs overexpressing V5-tagged WT YAP, S6A YAP, or empty vector control (EV) as detected by fluorescence (V5 tag) and chemiluminescence (YAP). (J) p-YAP S112, total YAP and HSC70 western blot analysis of PKN2 WT or KO PSCs with empty vector (EV), WT YAP or S6A YAP (S6A). (K) Hemocytometer cell count of EV , WT YAP or YAP S6A PSCs (PKN2 WT and KO) grown in 6-well plates for 8 days in triplicate (n=3, 2-way ANOVA with Tukey's multiple comparisons test).

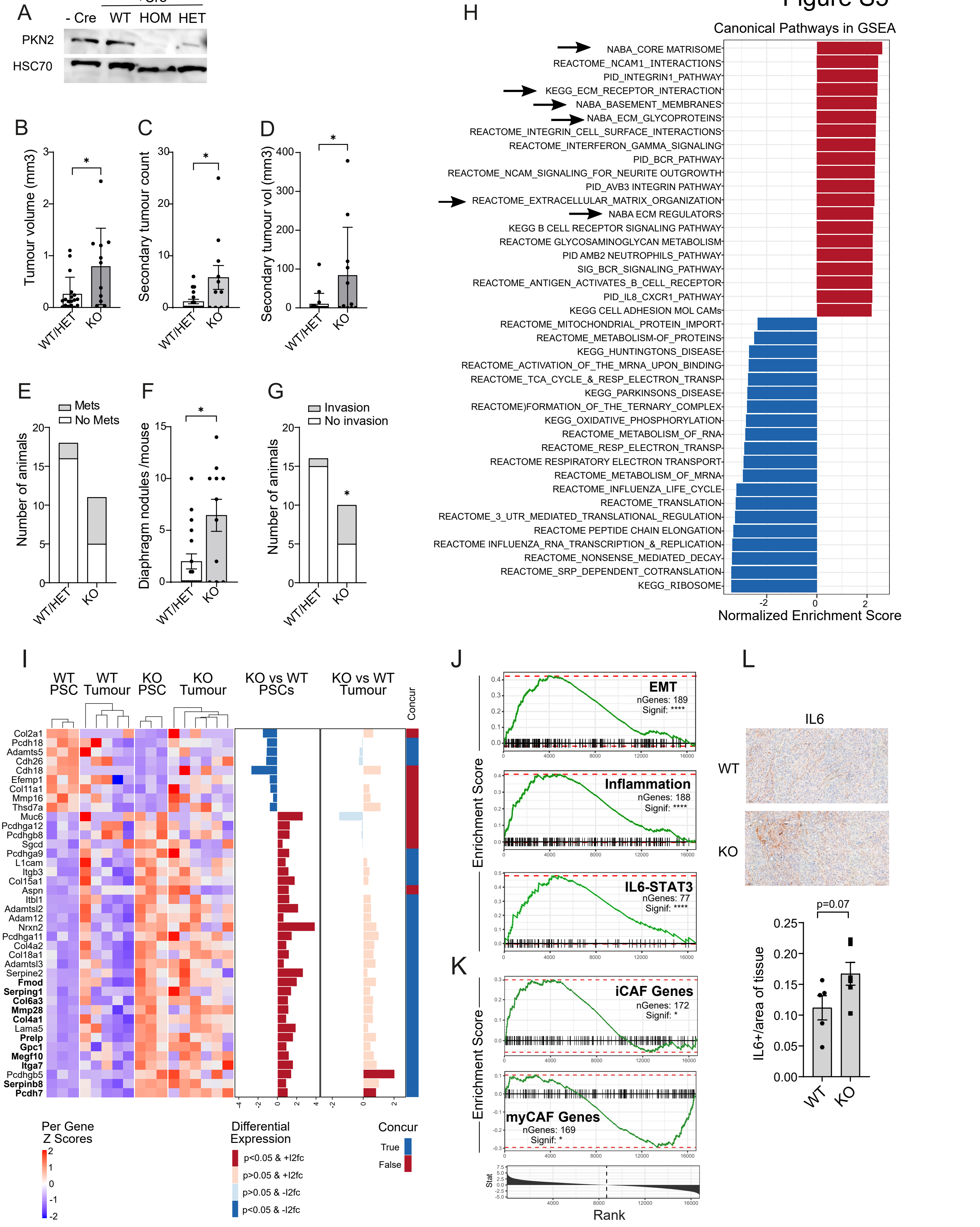

Supplemental Figure 5 (Related to Figure 5). Deletion of stromal PKN2 results in an enhanced iCAF phenotype in tumours in vivo. (A) Western blot of PKN2 and HSC70 in pancreas from Cre negative mice and Cre+ PKN2WT, PKN2KO and PKN2HET littermates. (B-D) Quantification of primary tumour volume (B) secondary tumour count per mouse and secondary tumour volume in controls (PKN2WT and PKN2HET) versus PKN2KO (n=8-11, Student's t-test, *p<0.05). (E-G) Quantification of number of animals with (grey) and without (white) peritoneal metastasis (Fisher's exact test), the number of diaphragmatic nodules per mouse (Student's t-test) (F) and the number of animals with (grey) and without (white) sites of invasion observed in cross-sections of the primary tumour (G) (n=8-11, *p<0.05, Fisher's exact test). (H) GSEA of orthotopic tumours showing the 20 most highly upregulated (red) and downregulated (blue) canonical pathways in PKN2KO tumours compared with WT tumours. (I) Comparison of transcriptomic expression data from WT and PKN2KO PSCs, with RNA-Seq data from tumours grown in WT and PKN2KO mice for DE ECM genes identified in Figure 2C. (J) GSEA enrichment plots for EMT, inflammation and IL6-Jak-STAT3 signalling in orthotopic tumours grown in PKN2KO mice as compared to WT mice (NES >2.0). (K) GSEA enrichment plots of genes upregulated in either iCAFs (bottom panel; NES = 1.41) or myCAFs (top panel; NES= -1.38) in PKN2KO tumours over PKN2WT tumours. (L) Representative images and quantification of IL6 staining in tumours grown in WT and PKN2KO mice.

Figure S6

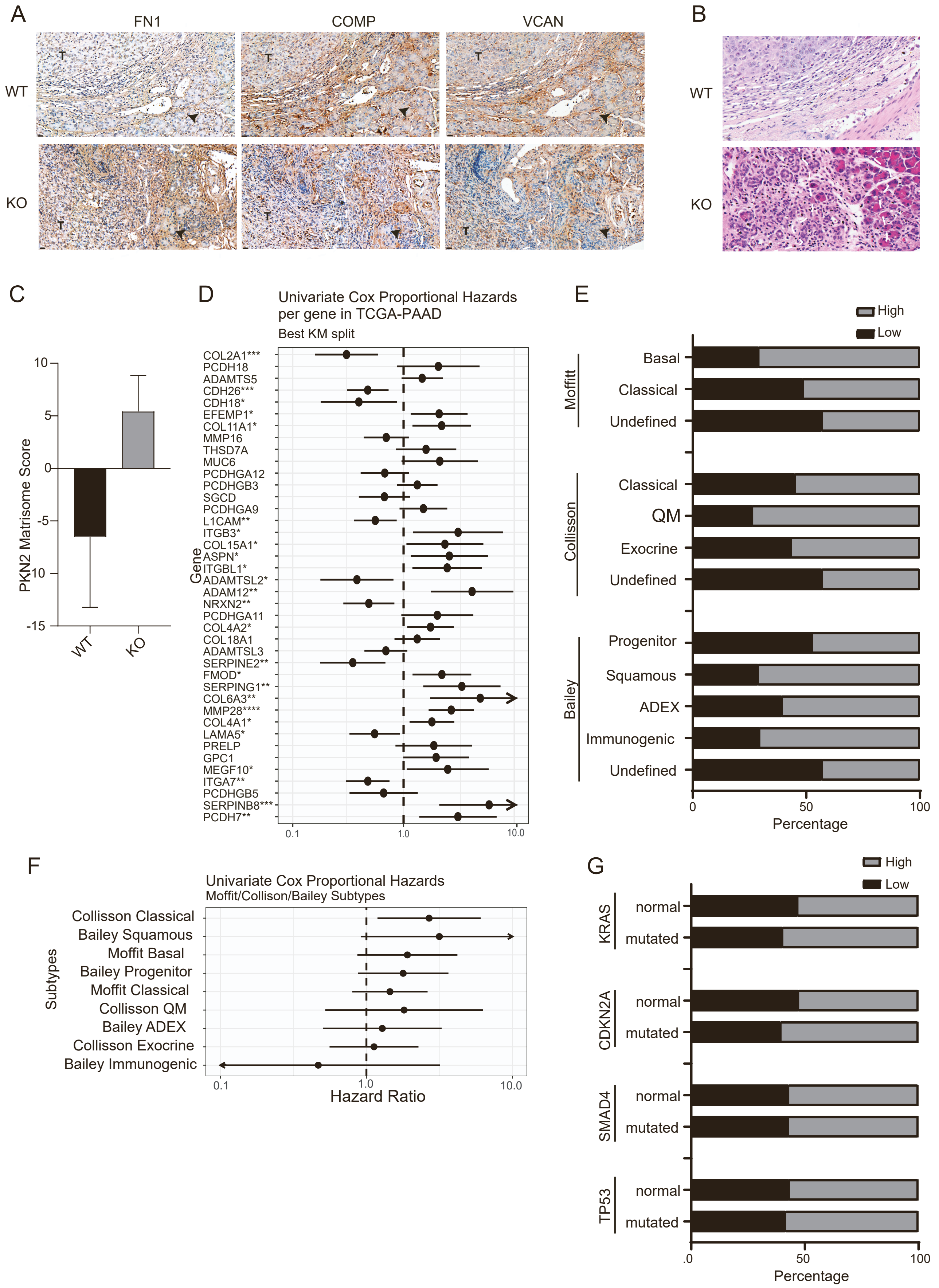

Supplemental Figure 6 (Related to Figure 6). PKN2^{KO} matrisome score identifies PDAC patients with poor prognosis. (A) IHC staining for ECM components FN1, COMP and VCAN, at the tumour edge or invasive front of representative WT (top panels) or PKN2^{KO} (bottom panels) tumours, performed on consecutive sections. (B) H&E of WT (top) and KO (bottom) tumours on sections cut approximately 50μm before those used for MI staining. (C) PKN2^{KO} matrisome score in orthotopic tumours from PKN2 WT and KO mice; n=5-6. (D) Cox proportional hazards test per gene from the PKN2^{KO} matrisome score in TCGA-PAAD data. (E) Percentage of TCGA-PAAD samples within each subtype of the Moffitt, Collison and Bailey datasets exhibiting high (grey bars) and low (black bars) PKN2^{KO} matrisome score. (F) Univariate Cox proportional Hazards test comparing high and low PKN2^{KO} matrisome score within each tumour subtype from Moffitt, Collison and Bailey datasets. (G) Percentage of samples with high or low PKN2^{KO} matrisome score that are WT or mutated for key PDAC driver genes.

SUPPLEMENTAL TABLES

Table S3 (Related to Figure 6): PKN2^{KO} ECM signature

Mouse_ensembl_gene_id	Mgi_symbol	Description	Human_ensembl_gene_id	Hgnc_symbol
ENSMUSG00000020682	Mmp28	matrix metallopeptidase 28 (epilysin) [Source:MGI Symbol;Acc:MGI:2153062]	ENSG00000271447	MMP28
ENSMUSG00000023224	Serping1	serine (or cysteine) peptidase inhibitor, clade G, member 1 [Source:MGI Symbol;Acc:MGI:894696]	ENSG00000149131	SERPING1
ENSMUSG00000024593	Megf10	multiple EGF-like-domains 10 [Source:MGI Symbol;Acc:MGI:2685177]	ENSG00000145794	MEGF10
ENSMUSG00000025348	Itga7	integrin alpha 7 [Source:MGI Symbol;Acc:MGI:102700]	ENSG00000135424	ITGA7
ENSMUSG00000026315	Serpinb8	serine (or cysteine) peptidase inhibitor, clade B, member 8 [Source:MGI Symbol;Acc:MGI:894657]	ENSG00000166401	SERPINB8
ENSMUSG00000029108	Pcdh7	protocadherin 7 [Source:MGI Symbol;Acc:MGI:1860487]	ENSG00000169851	PCDH7
ENSMUSG00000031502	Col4a1	collagen, type IV, alpha 1 [Source:MGI Symbol;Acc:MGI:88454]	ENSG00000187498	COL4A1
ENSMUSG00000034220	Gpc1	glypican 1 [Source:MGI Symbol;Acc:MGI:1194891]	ENSG00000063660	GPC1
ENSMUSG00000041559	Fmod	fibromodulin [Source:MGI Symbol;Acc:MGI:1328364]	ENSG00000122176	FMOD
ENSMUSG00000041577	Prelp	proline arginine-rich end leucine-rich repeat [Source:MGI Symbol;Acc:MGI:2151110]	ENSG00000188783	PRELP
ENSMUSG00000048126	Col6a3	collagen, type VI, alpha 3 [Source:MGI Symbol;Acc:MGI:88461]	ENSG00000163359	COL6A3

Table S5 (Related to STAR Methods- qPCR analysis): Oligonucleotides used in qPCR.

Resource/ Reagent		Source	Identifier
Name	Sequence		
IL-6-forward	5’-AGCCAGAGTCCTTCAGAGAGA-3’	This paper; Merck	N/A
IL-6-forward	5’-GGAGAGCATTGGAAATTGGGG-3’	This paper; Merck	N/A
Lif-Forward	5’-CGGCAACCTCATGAACCAGA-3’	This paper; Merck	N/A
Lif- Reverse	5’-GGAAACGGCTCCCCTTGA-3	This paper; Merck	N/A
Cxcl1-Forward	5’-CCAGAGCTTGAAGGTGTTG-3’	This paper; Merck	N/A
Cxcl1-Reverse	5’-AGCTTCAGGGTCAAGGCAAG-3’	This paper; Merck	N/A
Pparg- Forward	5’-GGGGATGTCTCACAATGCCA-3’	This paper; Merck	N/A
Pparg- Reverse	5’-TGGGTTCAGCTGGTCGATATC-3’	This paper; Merck	N/A
Plin2- Forward	5’-TGCCCATCATCCAGAAGCTG-3’	This paper; Merck	N/A
Plin2- Reverse	5’-AACAATCTCGGACGTTGGCT-3’	This paper; Merck	N/A
Diaph3- Forward	5’-GAGAAGCGACCCAAGTTGCAT-3’	This paper; Merck	N/A
Diaph3-Reverse	5’-GAAGGGGAGGTCTCTCTTTCTT-3’	This paper; Merck	N/A
Flna- Forward	5’-GTACCGTGTCCGGGCTGTGC-3’	This paper; Merck	N/A
Flna- Reverse	5’- ACATGCTCGCCACCGAAGCG-3’	This paper; Merck	N/A
Anln-Forward	5’-TGGGGCTGAGCAGATGGTCG-3’	This paper; Merck	N/A

Anln- Reverse	5’-TCCGGGACTGGCCATAACTGAAGA-3’	This paper; Merck	N/A
Ankrd1-Forward	5’-AAACGGACGGCACTCCACCG-3’	This paper; Merck	N/A
Ankrd1- Reverse	5’-CGCTGTGCTGAGAAGCTTGTCTCT-3’	This paper; Merck	N/A
Amotl2- Forward	5’-AACCGCCACCTGGCAAGCAA-3’	This paper; Merck	N/A
Amotl2- Reverse	5’-GGTCCTCGATGGCACCACGC-3’	This paper; Merck	N/A
Sdpr- Forward	5’-GCCCAGCAGGTGCGCTATGA-3’	This paper; Merck	N/A
Sdpr-Reverse	5’-CGGGGTGGCTTCCACGAGGT-3’	This paper; Merck	N/A
18s-Forward	5’-CACGGGAAACCTCACCCGGC-3’	This paper; Merck	N/A
18s- Reverse	5’-AACGGCCATGCACCACCACC-3’	This paper; Merck	N/A

Table S6 (Related to STAR Methods- siRNA transfection): Oligonucleotides used in siRNA experiment.

Reagent		Source	Identifier	
Reagent	Sequence		Pool Identifier	Identifier
siCtrl #1	UAGCGACUAAACACAUCAA	Dharmacon		D-001210-01-20
siPKN2 #1	GGAAAGUUACAACAGAUAA	Dharmacon	M-065210-02	D-065210-01
siPKN2 #2	UAUCCAAGGUUCUUAUCUA	Dharmacon	M-065210-02	D-065210-02
siPKN2 #3	CGACAUCAAGGAUCGAAUA	Dharmacon	M-065210-02	D-065210-03
siPKN2 #17	AGACUAGUGAGACGAGCUA	Dharmacon	M-065210-02	D-065210-17
siYAP1	GGAGAAGUUUACUACAUAA	Dharmacon	M-046247-01	D-046247-01
siYAP1	CCACCAAGCUAGAUAAAGA	Dharmacon	M-046247-01	D-046247-02
siYAP1	GAGAUGCAAUGAACAUAGA	Dharmacon	M-046247-01	D-046247-03
siYAP1	CAAUAGUUCCGAUCCCUUU	Dharmacon	M-046247-01	D-046247-04
