# Supplementary material for: Disruption of pancreatic stellate cell myofibroblast phenotype promotes pancreatic tumor invasion
Source: Cell Rep. 2022 Jan 25;38(4):110227. doi: 10.1016/j.celrep.2021.110227 (PMC8810397; doi:10.1016/j.celrep.2021.110227)
Supplement: Document S2. Article plus supplemental information [file mmc5.pdf]

Cell Reports

Article

Disruption of pancreatic stellate cell myofibroblast phenotype promotes pancreatic tumor invasion

Graphical abstract

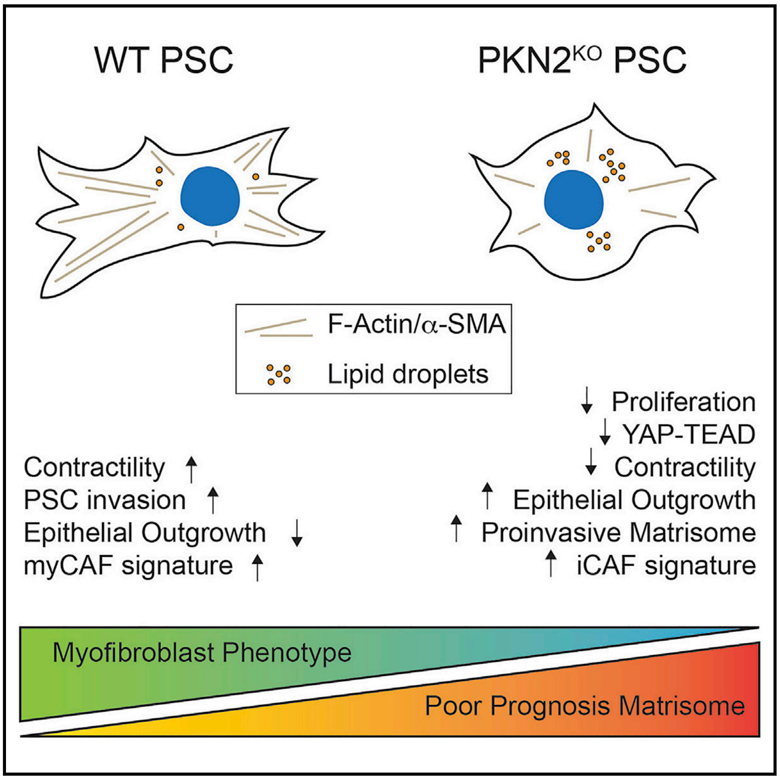

Highlights

- The Rho effector kinase PKN2 is a key regulator of myofibroblast phenotypes
- PKN2^{KO} induces a myofibroblast to inflammatory CAF switch in mouse pancreatic tumors
- Stromal deletion of PKN2 promotes more locally invasive orthotopic pancreatic tumors
- A PKN2^{KO} matrisome signature predicts poor outcome in human pancreatic cancer

Authors

Elizabeth R. Murray, Shinelle Menezes, Jack C. Henry, ..., Pedro Cutillas, John F. Marshall, Angus J.M. Cameron

Correspondence

a.cameron@qmul.ac.uk

In brief

Murray and Menezes et al. show that the Rho effector kinase PKN2 is essential for maintaining the myofibroblast phenotype of pancreatic stellate cells. Deletion of stromal PKN2 induces a switch to an inflammatory CAF phenotype both *in vitro* and *in vivo*, and this is associated with more invasive pancreatic tumors.

Murray et al., 2022, Cell Reports *38*, 110227
January 25, 2022 © 2021 The Author(s).
https://doi.org/10.1016/j.celrep.2021.110227

CellPress

Cell Reports

CellPress
OPEN ACCESS

Article

Disruption of pancreatic stellate cell myofibroblast phenotype promotes pancreatic tumor invasion

Elizabeth R. Murray,[1,7] Shinelle Menezes,[1,7] Jack C. Henry,[1] Josie L. Williams,[1] Lorena Alba-Castellón,[1] Priththivika Baskaran,[1] Ivan Quétier,[1] Ami Desai,[1] Jacqueline J.T. Marshall,[2] Ian Rosewell,[3] Marianthi Tatari,[4] Vinothini Rajeeve,[4] Faraz Khan,[4] Jun Wang,[4] Panoraia Kotantaki,[4] Eleanor J. Tyler,[4] Namrata Singh,[1] Claire S. Reader,[4] Edward P. Carter,[4] Kairbaan Hodivala-Dilke,[4] Richard P. Grose,[4] Hemant M. Kocher,[4,5] Nuria Gavara,[6] Oliver Pearce,[4] Pedro Cutillas,[4] John F. Marshall,[4] and Angus J.M. Cameron[1,8,*]
[1]Kinase Biology Laboratory, Barts Cancer Institute, Queen Mary University of London, John Vane Science Centre, Charterhouse Square, London EC1M 6BQ, UK
[2]Protein Phosphorylation Laboratory, Francis Crick Institute, 1 Midland Road, London NW1 1AT, UK
[3]Transgenic Services, Francis Crick Institute, 1 Midland Road, London NW1 1AT, UK
[4]Barts Cancer Institute, Queen Mary, University of London, John Vane Science Centre, Charterhouse Square, London EC1M 6BQ, UK
[5]Barts and the London HPB Centre, The Royal London Hospital, Barts Health NHS Trust, Whitechapel, London E1 1BB, UK
[6]Unitat de Biofísica i Bioenginyeria, Facultat de Medicina i Ciències de la Salut, Universitat de Barcelona, Barcelona, Spain
[7]These authors contributed equally
[8]Lead contact
*Correspondence: a.cameron@qmul.ac.uk
https://doi.org/10.1016/j.celrep.2021.110227

SUMMARY

In pancreatic ductal adenocarcinoma (PDAC), differentiation of pancreatic stellate cells (PSCs) into myofibroblast-like cancer-associated fibroblasts (CAFs) can both promote and suppress tumor progression. Here, we show that the Rho effector protein kinase N2 (PKN2) is critical for PSC myofibroblast differentiation. Loss of PKN2 is associated with reduced PSC proliferation, contractility, and alpha-smooth muscle actin (α-SMA) stress fibers. In spheroid co-cultures with PDAC cells, loss of PKN2 prevents PSC invasion but, counter-intuitively, promotes invasive cancer cell outgrowth. PKN2 deletion induces a myofibroblast to inflammatory CAF switch in the PSC matrisome signature both *in vitro* and *in vivo*. Further, deletion of PKN2 in the pancreatic stroma induces more locally invasive, orthotopic pancreatic tumors. Finally, we demonstrate that a $PKN2^{KO}$ matrisome signature predicts poor outcome in pancreatic and other solid human cancers. Our data indicate that suppressing PSC myofibroblast function can limit important stromal tumor-suppressive mechanisms, while promoting a switch to a cancer-supporting CAF phenotype.

INTRODUCTION

Fibroblasts play critical roles in mammalian development, homeostasis, and wound repair, where they dynamically regulate tissue structure through paracrine signaling and modulation of the extracellular matrix and connective tissue. During tissue remodeling and in response to inflammation, fibroblasts become activated into contractile alpha-smooth muscle actin (α-SMA)-positive myofibroblasts, which show enhanced extracellular matrix (ECM) deposition and matrix-remodeling activities. In fibrotic diseases and many solid cancers, the chronic activation of fibroblasts into myofibroblasts contributes directly to disease pathology and prognosis. In the pancreas, the predominant resident fibroblast cell type is the pancreatic stellate cell (PSC), characterized by lipid and vitamin storage droplets and intermediate filament expression (Apte et al., 1998; Froeling et al., 2011). In pancreatic ductal adenocarcinoma (PDAC), resident PSCs become activated in response to tumor-derived paracrine signals, such as transforming growth factor-β (TGF-β), sonic hedgehog (Shh), and platelet-derived growth factor (PDGF), resulting in desmoplastic, hypovascular tumors, which respond poorly to therapy. The reciprocal interaction between malignant PDAC cells and PSCs has therefore attracted increasing attention clinically, and identifying targets to modify PSC function is a priority (Froeling and Kocher, 2015; Kocher et al., 2020).

We reported recently that the Rho effector kinase, protein kinase N2 (PKN2), but not PKN1 or PKN3, plays a critical role during developmental expansion of the embryonic mesoderm (Quetier et al., 2016). Loss of PKN2 suppressed proliferation and migration of mesenchymal fibroblasts both *in vivo* and *in vitro*, since independently corroborated (Danno et al., 2017; Yang et al., 2017). Collapse of the mesodermal tissue and associated vasculature results in lethality at embryonic day 10 (E10), with failure in axial turning and morphogenetic defects indicating defective mesenchymal contractility. We hypothesized that PKN2 may play a conserved role in the expansion and activation

This is an open access article under the CC BY license (http://creativecommons.org/licenses/by/4.0/).

of fibroblasts into cancer-associated fibroblasts (CAFs) during tumor development and focused on pancreatic cancer as the archetype of desmoplastic myofibroblast-rich tumors. Recent work has identified several subpopulations of CAFs in PDAC, including myofibroblastic and secretory subtypes (Biffi et al., 2019; Elyada et al., 2019; Hutton et al., 2021; Neuzillet et al., 2019; Öhlund et al., 2017; Steele et al., 2021). Understanding how PKN2 contributes to specific CAF traits thus has the potential to define novel ways to modulate PDAC tumor biology.

Here, we report that PKN2 regulates both the activation of mouse PSCs and mouse embryonic fibroblasts (MEFs) into myofibroblasts. We identify PKN2 as a novel regulator of the mechanosensor YAP, which is central to myofibroblast function. Intriguingly, loss of PKN2 in PSCs results in a switch in cellular invasive mechanism in heterotypic spheroid cultures, suppressing PSC invasion while promoting polarized epithelial outgrowth. Further, stromal deletion of PKN2 *in vivo* results in more locally invasive tumors, with accompanying pro-invasive changes to the matrisome signature. Preventing myofibroblast differentiation in malignancy may therefore limit the tumor-suppressive role of fibroblasts, counter to the dogma that CAFs support cancer invasion. This work also highlights the potential impact that targeting specific fibroblast phenotypes may have on functionally distinct CAF subtypes in PDAC.

RESULTS

PKN2 regulates PSC growth and TGF-β1-induced myofibroblast differentiation

To generate a model in which the role of PKN2 in PSC function could be assessed, inducible PKN2 knockout (KO) PSCs were derived from the pancreas of a Rosa26CreERT2$^{+/WT}$ PKN2$^{fl/fl}$ mouse by Histodenz cushion centrifugation (Apte et al., 1998; Bachem et al., 1998; Vonlaufen et al., 2010). Isolated cells stained positively for the PSC markers α-SMA, desmin, glial fibrillary acidic protein (GFAP), and vimentin (Figure S1A). Storage of lipid droplets in the cytoplasm was also detected by staining with Oil Red O and was increased by treatment with all-*trans* retinoic acid (ATRA), a defining feature of stellate cells (Figure S1B; Apte et al., 1998; Bachem et al., 1998; Froeling et al., 2011). Penetrant loss of PKN2 protein expression was observed 96 h after a 2-h acute treatment of PSCs with 2 μM 4-hydroxytamoxifen (4-OHT) (Figure S1C), indicating penetrant Cre recombination. This treatment regimen was used to generate PKN2^{KO} PSCs.

We previously reported that induced PKN2 deletion in developing embryos results in a mesenchymal-specific loss of proliferation in the mesoderm (Quetier et al., 2016). This was further corroborated in isolated MEFs, where induced PKN2 recombination causes accumulation of cells in G0/G1 (Quetier et al., 2016). Here, we also observed a reduction in PSC growth rate in 2D culture following PKN2 deletion (Figure 1A) and that PKN2^{KO} PSCs arrest at a lower maximum cell density than wild-type (WT) PSCs (Figure 1B); individual cells are slightly smaller in 2D culture following PKN2^{KO} (Figure S1D). The slower growth rate of PKN2^{KO} PSCs was accompanied by a decrease in cyclin D1 expression levels (Figure 1C) but little change in cell cycle profile; importantly, there was no increase in the G2 fraction, as PKN2 deletion has been previously reported to cause G2 arrest and cytokinesis failure in other cell types (Schmidt et al., 2007; Figure 1D).

We next examined the effect of deleting PKN2 on myofibroblast differentiation. PSCs become activated toward a myofibroblast phenotype upon adherence in 2D tissue culture (Apte et al., 1998), which can be further exacerbated through stimulation with TGF-β. To phenotypically assess myofibroblast function, we conducted collagen gel contractility assays. Both unstimulated and TGF-β1-stimulated collagen gel contractility of PSCs was reduced by PKN2 deletion (Figures 1E and 1F). TGF-β1-induced collagen gel contractility was also reduced in MEFs following PKN2 deletion (Figure S1E). Consistent with reduced contractility (Hinz et al., 2001), PKN2 loss was also associated with a reduction in α-SMA fibers in unstimulated and TGF-β1-treated cells (Figures 1G and 1H). Indeed, in PKN2^{KO} PSCs, TGF-β1 stimulation marginally suppressed α-SMA fibers, indicating a clear switch in TGF-β1 signal output (Figure 1H). α-SMA fiber induction by TGF-β1 was similarly lost in PKN2^{KO} MEFs (Figure S1F). F-actin levels were also reduced in PKN2^{KO} PSCs (Figures S1G and S1H). However, total expressed α-SMA protein levels were not reduced following PKN2 deletion in PSCs (Figures S1I and S1J). Our data indicate that PKN2 plays a role in the adoption of a contractile myofibroblast phenotype stimulated by 2D adherence or TGF-β1. In addition, PKN2^{KO} PSCs exhibited enhanced storage of lipid droplets as assessed by Oil Red O (Figures 1I and 1J). Lipid droplets are considered a key marker of quiescent PSCs, lost upon acquisition of a myofibroblast phenotype (Apte et al., 1998; Froeling et al., 2011). Changes induced by PKN2 loss resemble those induced by ATRA, which has been demonstrated to de-differentiate PSCs in PDAC in pre-clinical and phase I clinical studies (Carapuca et al., 2016; Froeling et al., 2011; Kocher et al., 2020). Reduced cell growth, loss of contractility, and enhanced lipid storage all indicate that PKN2 loss suppresses adoption of an activated myofibroblast phenotype.

PKN2 loss suppresses PSC mechanosensing and modulates the extracellular matrix

PSCs play a critical role in the maintenance of tissue homeostasis through the regulation of the ECM. In turn, the activation status of PSCs is reciprocally regulated by the composition and rigidity of the ECM through mechanosensing pathways. We therefore sought to explore the interaction between PSCs and the ECM. First, we profiled the expression status of 411 gene transcripts associated with the ECM and cell adhesion (QIAGEN). WT and PKN2^{KO} PSCs were cultured in complete medium or exposed to TGF-β1 for 72 h. PKN2 deletion predominantly resulted in the upregulation of ECM-associated genes under 2D cell culture conditions (Figures 2A and 2B), including genes associated with metastasis (Serpine2, Fmod, Itgbl1, Aspn, MMP28, and Col6a3; Buchholz et al., 2003; Liu et al., 2021; Wiechec et al., 2021). Many of these genes were also differentially expressed (DE) in PKN2^{KO} PSCs compared with WT PSCs following TGF-β1 stimulation (Figures 2B and 2C; Table S1).

A number of studies have examined PSC and CAF expression signatures from human PDAC patients and mouse pancreatic

CellPress
OPEN ACCESS

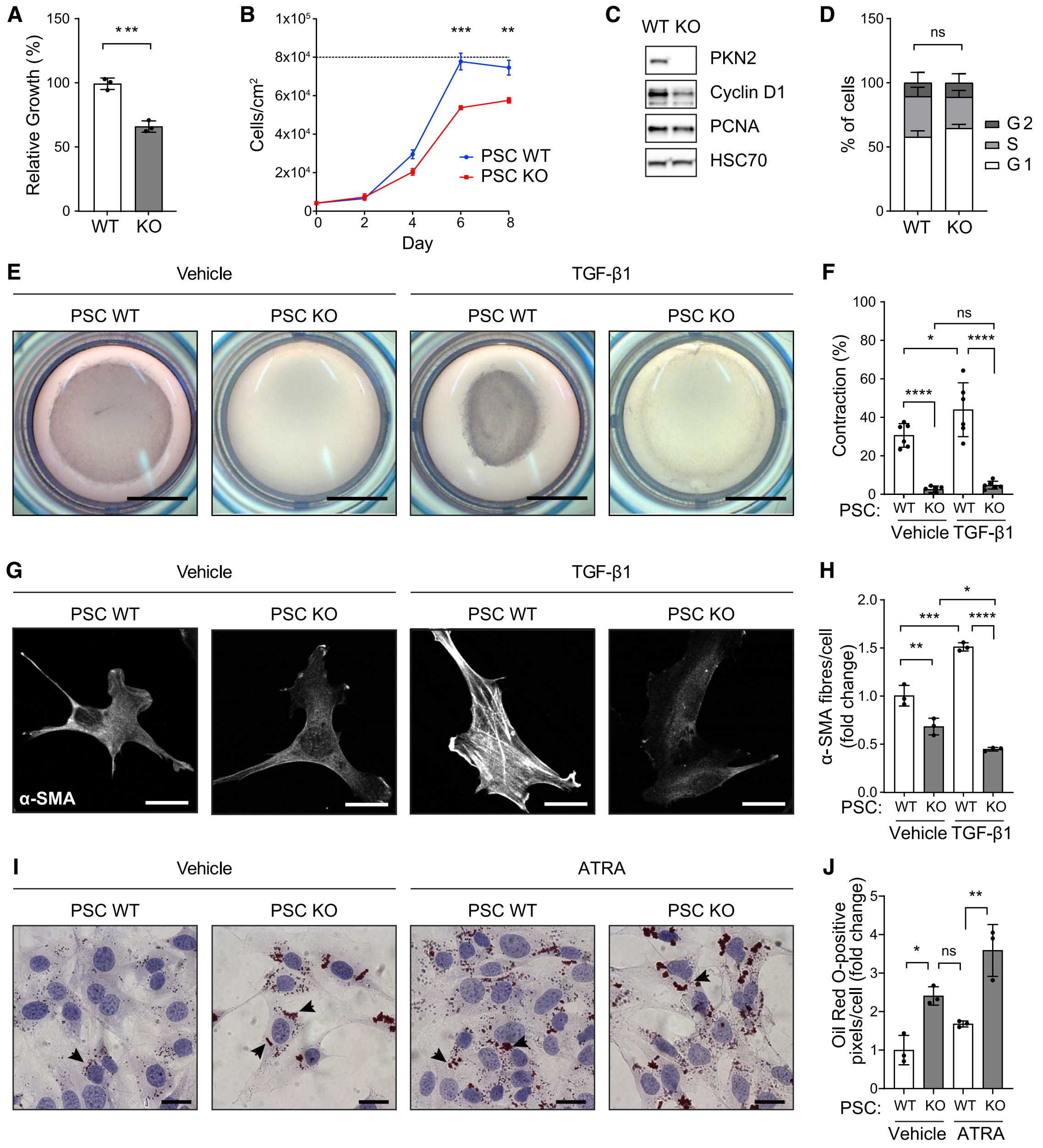

Figure 1. PKN2 loss reduces PSC growth and myofibroblast differentiation

(A) Growth of immortalized WT and PKN2^{KO} PSCs relative to WT PSCs on day 4 as assessed by MTT assay (n = 3; unpaired t test).

(B) Density of WT and PKN2^{KO} PSCs grown to confluency (8 days post-seeding) relative to maximum density of WT cells (n = 3; unpaired t test).

(C) Western blot of cyclin D1, proliferating cell nuclear antigen (PCNA), and housekeeping HSC70 in WT and PKN2^{KO} PSCs (n = 5).

(D) Percentage of WT and PKN2^{KO} PSCs in G1, S, and G2 of the cell cycle (n = 3; two-way ANOVA with Sidak's test). ns, not significant.

(E and F) Representative images and quantification of gel contraction from embedded WT and PKN2^{KO} PSCs treated with 5 ng/mL TGF-β1 or vehicle for 72 h using the formula (1 − ratio of gel size/well size) × 100. Scale bar represents 5 mm; (n = 2).

(G and H) Representative images and quantification of absolute number of α-SMA fibers in WT and PKN2^{KO} PSCs treated with vehicle or 5 ng/mL TGF-β1 for 72 h. Scale bar represents 25 μm. Quantification is relative to vehicle-treated WT PSCs using MATLAB algorithm (n = 3).

(I and J) Representative images (I) and quantification (J) of Oil Red O staining (arrows) of immortalized PSCs plated on glass coverslips and treated with vehicle or ATRA daily for 4 days (n = 3; scale bar represents 25 μm).

(F, H, and J) Statistics are two-way ANOVA with Tukey's multiple comparisons test.

*p < 0.05, **p < 0.01, ***p < 0.001, and ****p < 0.0001.

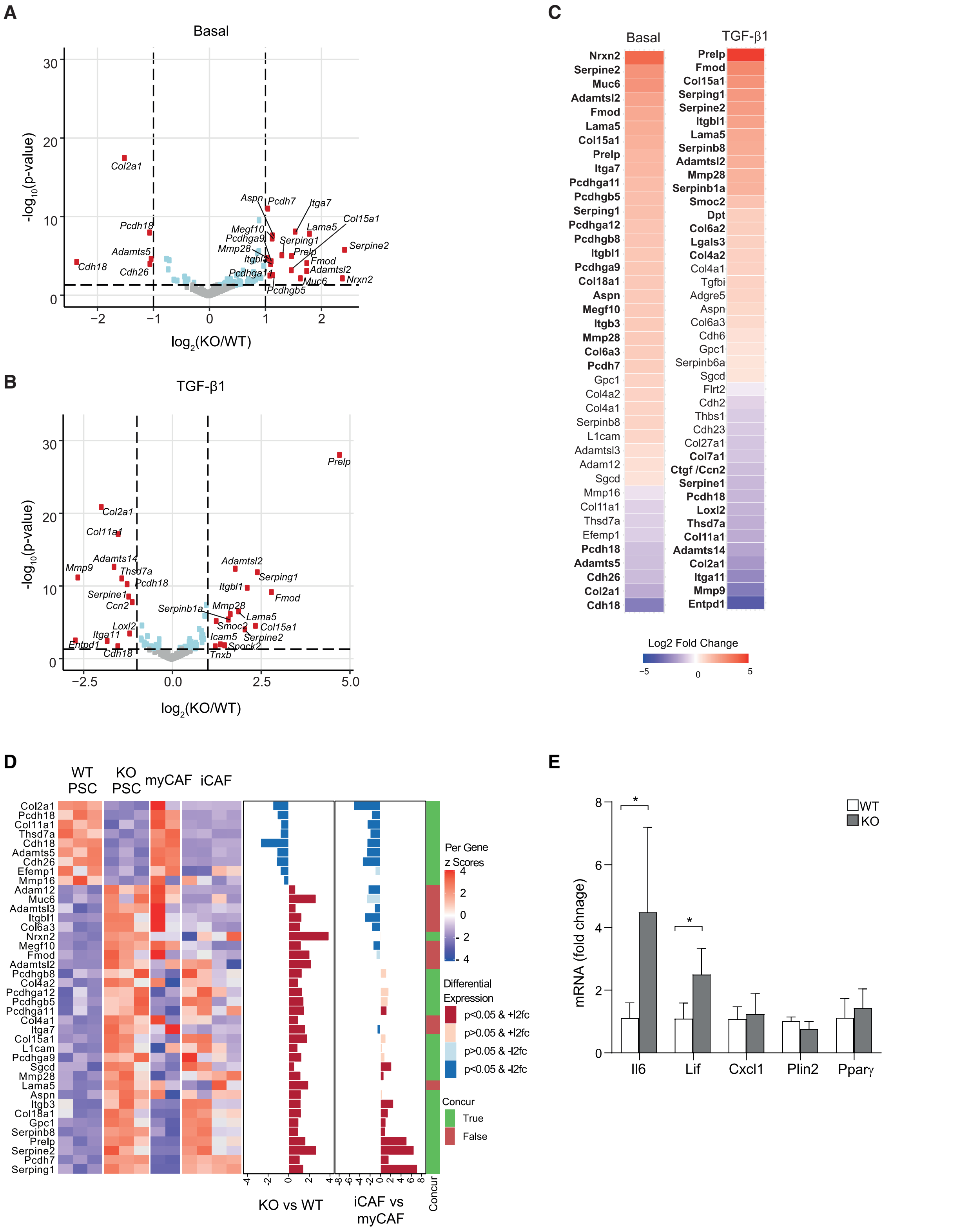

Figure 2. Deletion of PKN2 promotes a CAF-like ECM signature in PSCs

(A and B) Differentially expressed (DE) ECM and adhesion gene transcripts (QIAseq) in PKN2^{KO} PSCs relative to WT PSCs treated with vehicle (A) or 5 ng/mL TGF-β1 (B) for 72 h. Log2 fold change and p values determined by DESeq2 (n = 3; $p < 0.05$).

(legend continued on next page)

cancer models, defining distinct CAF expression patterns and fibroblast subtypes. Öhlund et al. initially proposed the existence of at least two spatially distinct CAF populations, termed myofibroblastic CAFs (myCAFs) and inflammatory CAFs (iCAFs) (Öhlund et al., 2017). Strikingly, the changes in matrisome expression between WT and KO PSCs were very similar to those observed between myCAFs and iCAFs, respectively (Figure 2D). To further explore this, we examined the expression of a panel of CAF signature genes by qRT-PCR (Öhlund et al., 2017); the iCAF markers Il6 and Lif were significantly upregulated following PKN2 loss, while the quiescence- and lipid-droplet-associated genes Pparγ and Plin2 were largely unchanged (Figure 2E). This supports a switch toward an iCAF phenotype in PKN2^{KO} PSCs rather than induction of quiescence. Examination of publicly available single-cell RNA sequencing data indicates that PKN2 is, however, expressed in both myCAF and iCAF populations, as well as in PSCs and other tumor cell types (Figures S2A and S2B; Biffi et al., 2019; Peng et al., 2019).

Djurec et al. (2018) also isolated a panel of normal pancreatic fibroblasts (NPFs) and CAFs from a genetically engineered C57BL/6 pancreatic cancer model and compared their transcriptomes. Many of the gene expression changes induced by PKN2 loss—particularly those showing decreased expression—were also mirrored in CAFs when compared with NPFs from the Djurec et al. study (Djurec et al., 2018; Figure S2C). Upregulation of pro-metastatic ECM genes and overlap with distinct CAF signatures suggests that suppressing myofibroblast functions of PSCs may support expression of a cancer-promoting iCAF signature.

To identify pathways underlying these PKN2-dependent myofibroblast and ECM changes, we used a panel of transcriptional reporters to probe the TGF-β1-SMAD pathway and the mechanotransduction- and Rho-responsive transcriptional regulators YAP and MRTF (Figure 3A; Calvo et al., 2013; Crider et al., 2011; Small, 2012). For the mechanosensing transcriptional regulators YAP and MRTF, we used luciferase reporters to measure TEAD- (Mahoney et al., 2005) and SRF (Promega)-driven transcription, respectively. Serum stimulation resulted in significant activation of SRF transcription, but this was minimally affected by loss of PKN2 (Figure 3B). In contrast, TEAD-driven transcription was significantly reduced under serum-starved and serum-stimulated conditions, implicating PKN2 as a novel regulator of YAP (Figure 3C). TEAD-driven transcription was lower in WT PSCs treated with TGF-β1 for 24 h compared with untreated controls, though the pattern of reduction with PKN2^{KO} was consistent with other conditions (Figure 3C). TEAD transcription was also compromised in PKN2-deleted MEFs, suggesting a common mechanism in distinct mesenchymal lineages (Figure S3A). As expected, TGF-β1 strongly induced SBE-luciferase expression in PKN2 WT PSCs, although this was not suppressed by PKN2 loss (Figure 3D). Thus, PKN2 loss results in reduced YAP-TEAD signaling, whereas SMAD and MRTF stimulation remains largely intact. Consistent with this, a number of CAF-associated YAP target genes (Calvo et al., 2013) show reduced expression following PKN2 deletion as assessed by qRT-PCR (Figure 3E). Foster et al. also comprehensively assessed YAP target genes in CAFs, and eight of these are present in the QIAseq ECM panel (Foster et al., 2017); TGF-β1 induction of most of these YAP targets was also suppressed following PKN2 loss (Figure S3B). PKN2-regulated YAP targets include the key myofibroblast marker Ctgf (Öhlund et al., 2017) and direct myofibroblast function regulators, Ankrd1 and Serpine1 (Masuda et al., 2019; Samaras et al., 2015). Together, these data identify the YAP-TEAD axis as a target of PKN2 in myofibroblast phenotype PSCs.

We next examined whether PKN2 loss impacts YAP nuclear localization and phosphorylation. Intriguingly, both PSCs (Figure 1B) and MEFs (Quetier et al., 2016) undergo growth arrest at reduced cell densities in the absence of PKN2, a phenotype associated with TEAD loss-of-function mutants (Ota and Sasaki, 2008). As YAP nuclear localization is modulated by cell density, we developed a CellProfiler pipeline to count cell neighbors and assessed the relationship between local cell density and YAP nuclear localization. Although the percentage of cells showing nuclear staining of YAP was comparable (Figures 3F and 3G), quantitation of YAP staining revealed a significant reduction in nuclear YAP intensity following PKN2 loss at all densities tested (Figure 3H). This was phenocopied in MEFs (Figures S3C–S3E). As expected, nuclear localization decreased with greater local cell density (Figure 3H). This suggests that PKN2 can promote YAP nuclear localization under basal conditions of cell growth. YAP-TEAD activity and nuclear localization is canonically regulated through inhibitory phosphorylation by the Hippo pathway kinase LATS. Loss of PKN2 was associated with little change in phosphorylation of YAP on the LATS site Ser112 (Figures 3I and 3J), equivalent to Ser127 in human YAP1. S112 phosphorylation was, however, robustly increased at high cell density in both WT and KO cells, consistent with functional Hippo-pathway-mediated contact inhibition (Ege et al., 2018; Zhao et al., 2007).

Consistent with the SBE-driven reporter expression, TGF-β1-induced phosphorylation of SMAD2/3 and nuclear translocation of SMAD4 were not suppressed by PKN2 loss in PSCs (Figures 3K, 3L, S3F, and S3G) or MEFs (Figures S3H–S3J), further indicating that PKN2 is not required for canonical TGF-β signaling. However, reduction in coupling of TGF-β1 to p70S6 kinase and ERK was observed in PKN2^{KO} PSCs (Figure 3M), indicating a potential role in non-canonical TGF-β1 signaling.

Together, our data indicate that PKN2 loss from PSCs decreases transcription promoted by the mechanosensor YAP and disrupts myofibroblast function while inducing a switch toward an iCAF ECM and inflammatory signature.

(C) DE gene transcripts in PKN2^{KO} PSCs relative to WT PSCs treated with vehicle or 5 ng/mL TGF-β1 for 72 h; transcripts in bold were at least halved or doubled in expression between WT and KO (n = 3; $p < 0.05$).

(D) Comparison of transcriptomic expression data between WT and PKN2^{KO} PSCs and CAF expression data from Öhlund et al. (2017), using the panel of DE ECM genes with significance greater than $p < 0.05$ (C). Concurrence of changes between the two datasets is indicated in the righthand side bar (concur).

(E) qPCR analysis of mRNA expression of Il6, Lif, Cxcl1, Plin2, and Pparγ in WT and PKN2^{KO} PSCs expressed as fold change to WT for each gene (n = 4; *$p < 0.05$; ratio paired t-rest).

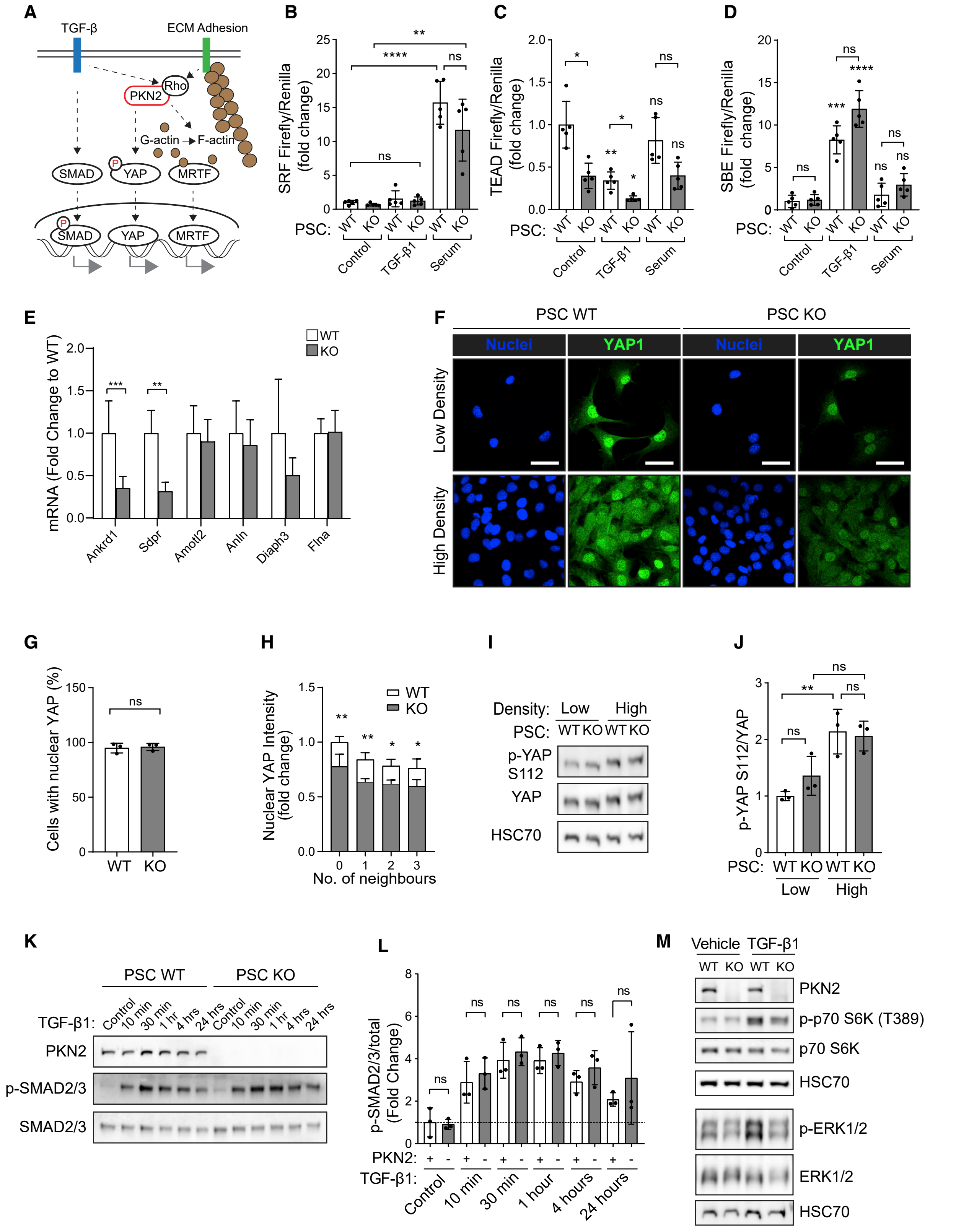

(legend on next page)

CellPress
OPEN ACCESS

Deletion of PKN2 from PSCs modifies the mode of PDAC cell invasion

As the Rho-YAP axis is implicated in CAF function (Calvo et al., 2013; Dupont et al., 2011; Wada et al., 2011; Zhao et al., 2012), we next sought to examine whether PKN2 loss could impact the reciprocal interaction between PSCs and pancreatic cancer (PDAC) cells. Induction of PDAC cell growth and invasion by PSCs has been extensively reported (Drifka et al., 2016; Eguchi et al., 2013; Heinrich et al., 2013; Kozono et al., 2013; Vonlaufen et al., 2008). We co-cultured our inducible PKN2^{KO} PSCs with mouse PDAC cell lines derived from *Pdx1-Cre; K-RAS*$^{+/LSL.G12D}$*; p53*$^{R172H/+}$ (KPC) or Pdx1-flp; *K-RAS*$^{+/LSL.G12D}$*; p53*$^{R172H/+}$ (KPF) mice (Schonhuber et al., 2014). TB32048 (KPC) and R254 (KPF) mouse PDAC cell lines were cultured alone or in co-culture with WT PSCs or PKN2^{KO} PSCs.

To assess proliferation of both PDAC cells and PSCs in co-culture, we generated TB32048 and R254 cells stably expressing Firefly luciferase and inducible PKN2^{KO} PSCs stably expressing Renilla luciferase by lentiviral transduction; cell growth can then be assessed in each population using the Dual-Glo luciferase assay system (Promega). WT and PKN2^{KO} PSCs were cultured alone or in co-culture with either TB32048 or R254 PDAC cells in 0.5% serum for 72 h. Both WT and PKN2^{KO} PSCs enhanced growth of both PDAC cell lines (Figures S4A and S4B). TB32048, but not R254, cells also reciprocally enhanced the growth of co-cultured PSCs (Figures S4C and S4D). Together, these data indicate that PSCs can support enhanced PDAC cell growth independently of PKN2 status.

To examine 3D interactions, spheroid co-cultures were generated by resuspension of PSCs and PDAC cells in hanging droplets containing methylcellulose (Leung et al., 2015; Ware et al., 2016). The following day, spheroids were collected and embedded in a 3D matrix in glass-bottomed 96-well plates. Invasion of cells from the center of the spheroid into the surrounding matrix was monitored by light microscopy. Invasion of PSCs and PDAC cells from spheroids into the matrix was confirmed by confocal microscopy. Neither TB32048 cells nor PSCs invaded when cultured alone (Figure S4E). Deletion of PKN2 suppressed the ability of PSCs to invade into the matrix in co-culture with TB32048 cells (Figures 4A, 4B, and S4F). Surprisingly, however, PKN2 deletion from PSCs also significantly enhanced epithelial cancer cell outgrowths from the surface of spheroids into the matrix (Figures 4A, 4C, and S4F–S4H). Small interfering RNA (siRNA)-induced suppression of either PKN2 or YAP1 also significantly suppressed PSC invasion while promoting invasive epithelial outgrowths from spheroids (Figures 4A–4C and S4F–S4H), corroborating the results seen with Cre-induced PKN2 deletion. This also confirms the central role for YAP1 in fibroblast-led invasion as a key mechanosensor (Calvo et al., 2013). Notably, the invasive polarized epithelial outgrowths were largely PSC negative (Figure 4A) and there was negative correlation between PSC invasion area and the area of these invasive epithelial outgrowths (Figure S4H). Importantly, however, outgrowths are not observed from PDAC cells grown alone (Figure S4E), indicating that this behavior is promoted by co-culture with PKN2 or YAP-depleted PSCs.

To confirm a role for YAP in PSC-driven invasion, we next examined the impact of transducing PSCs with lentiviral V5-tagged WT-YAP or constitutively active YAP-S6A (in which all inhibitory LATS target sites have been mutated to alanine; Rosenbluh et al., 2012; Figure S4I). Constitutively active YAP-S6A, but not WT-YAP, significantly enhanced PSC invasion in both WT and KO PSCs and rescued PKN2^{KO} suppression of invasion (Figures 4D and 4E). Retrovirally transduced stable cell lines induced more variable PDAC epithelial invasion from spheroids, and data showed little statistical significance (Figure 4F); epithelial outgrowths were, however, significantly diminished in YAP-S6A PSCs compared with empty vector (EV) or WT-YAP transduced cells. The failure of WT-YAP to phenocopy YAP-6A was surprising, so we assessed YAP activity status in our transduced cell lines. YAP-S6A induced a substantial increase in TEAD-reporter activity, whereas WT-YAP induced no increase above EV (Figure 4G). Further, WT-YAP overexpression resulted in enhanced total YAP and pYAP-S112 expression (Figure S4J). This indicates that overexpression of WT-YAP in our PSC model does not enhance YAP transcriptional activity but instead increases the expression of phosphorylated inactive YAP; this may have unexplored impact on the highly variable outgrowth seen with these WT-YAP cells (Figure 4F). Further, while constitutively active YAP-S6A robustly enhances PSC growth and rescues PKN2^{KO} growth suppression, WT-YAP has minimal impact, indicating it does

Figure 3. PKN2 modulates TEAD-driven transcription and nuclear localization of the mechanosensor YAP

(A) Schematic showing potential downstream targets of PKN2 involved in myofibroblast differentiation.

(B–D) Normalized expression of SRF (B), TEAD (C), or SMAD (D) responsive Firefly luciferase reporter in WT and KO PSCs starved in 0.5–1% serum or treated with 5 ng/mL TGF-β1 or 10% serum. Values are normalized to a Renilla luciferase control per sample and presented relative to WT serum-starved PSCs (n = 5; two-way ANOVA with Tukey's correction).

(E) qPCR analysis of expression of indicated genes in PKN2 WT and KO PSCs expressed as a fold change to WT control (n = 4).

(F) Immunofluorescent images of YAP1 localization (green) in WT and PKN2^{KO} PSCs plated at low and high density on glass coverslips for 48 h (minimum of 100 cells/condition; n = 3; scale bar represents 50 μm).

(G) Percentage of WT and PKN2^{KO} PSCs with YAP-positive nuclei plated at both high and low density (n = 3; unpaired t test).

(H) Quantification by Python CellProfiler algorithm of YAP nuclear intensity for indicated number of cell neighbors (n = 3; two-way ANOVA with Sidak's test).

(I and J) Representative western blot and quantification of p-YAP S112 and total YAP expression in WT and PKN2^{KO} PSCs plated at low and high density (n = 3; two-way ANOVA with Tukey's multiple comparisons test).

(K and L) Western blot and quantification of p-SMAD2/3 induction with 5 ng/mL TGF-β1 for indicated time points; quantification expressed relative to untreated WT PSCs (n = 3; unpaired t test).

(M) Western blot analysis of p-p70 S6K, total p70 S6K, p-ERK1/2, and total ERK in WT and PKN2^{KO} PSCs starved in 1% serum and treated with vehicle or 5 ng/mL TGF-β1 for 4 h (n = 2).

For statistics: *$p < 0.05$; **$p < 0.01$; ***$p < 0.001$; and ****$p < 0.0001$.

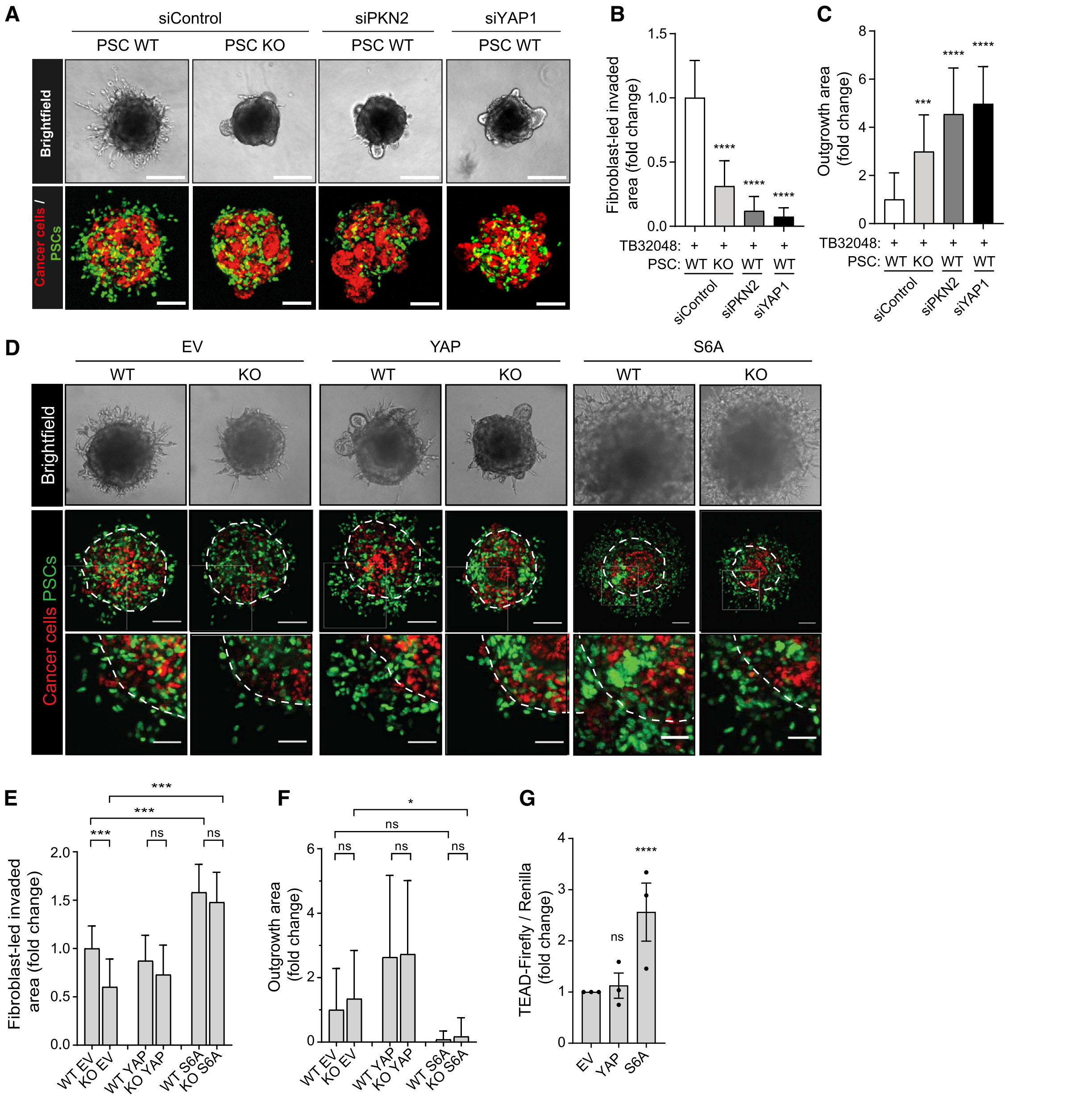

Figure 4. PKN2 loss reduces PSC-led cancer cell invasion but promotes cancer cell outgrowth.

(A) Bright-field (top; scale bar represents 200 μm) and live-cell confocal z stack projections (bottom; scale bar represents 100 μm) of spheroids (n > 16) containing H2B-RFP TB32048 PDAC cells (red) and H2B-GFP WT or PKN2^{KO} PSCs (green) embedded in Matrigel matrix for 3 days after siRNA treatment.

(B and C) Area of fibroblast-led invasion (B) or cancer cell outgrowth (C) per spheroid, normalized to total spheroid area and expressed as fold change relative to WT control (n > 16 spheroids/condition; one-way ANOVA with Tukey's multiple comparisons test).

(D) Bright-field (top panel) and confocal (bottom panels) images of spheroids containing TB32048 cancer cells with WT or PKN2^{KO} PSCs transduced with either empty vector (EV), YAP WT (YAP), or YAP S6A (S6A) vectors. Dotted white lines indicate core area of spheroid.

(E and F) Quantification of area of PSC-led (E) or epithelial (F) invasion, normalized to total spheroid area per spheroid, relative to EV (n = 3; two-way ANOVA with Tukey's multiple comparisons test).

(G) Dual luciferase analysis of TEAD reporter on WT PSCs transduced with EV, YAP, or S6A YAP.

Data expressed as Firefly or Renilla luminescence for each well relative to EV (n > 3; two-way ANOVA with Tukey's multiple comparisons test; *p < 0.05, ***p < 0.001, and ****p < 0.0001).

not act dominantly in these cells (Figure S4K). Together, our data indicate that high YAP activity in PSCs promotes PSC invasion while suppressing epithelial invasion in spheroid co-cultures (Figure 4F). Enhanced YAP activity also rescues PKN2^{KO} suppression of PSC invasion and growth, corroborating YAP as a functional PKN2 effector.

Our data suggest that, while PKN2 and YAP are important for the invasive capacity of PSCs, these cells may also be restraining malignant epithelial outgrowth, potentially through regulation of the ECM.

Deletion of stromal PKN2 *in vivo* promotes invasive multifocal tumors

We next wished to examine the impact of stromal PKN2 deletion on pancreatic tumors *in vivo*. PKN2 loss is embryonic lethal, but deletion in adult mice using the RosaCreERT$^{+/WT}$ PKN2^{flox} model is well tolerated and penetrant (Figure S5A). One thousand TB32048 (C57BL/6 background) cells were implanted into the pancreas to initiate syngeneic orthotopic tumor growth in both male and female littermate RosaCreERT$^{+/WT}$: PKN2$^{+/+}$ (WT), PKN2$^{fl/+}$ (heterozygous [HET]), and PKN2$^{fl/fl}$ (KO) mice (C57BL/6 background). All mice had been subjected to the same tamoxifen regime to control for any off-target effects of either tamoxifen or Cre (Figure 5A). Tumors were tracked by MRI, and the experiment was terminated at a single time point as a number of tumors within each cohort approached maximum size limits. Primary tumors were on average larger in the PKN2^{KO} cohort (Figures 5B, 5C, and S5B). There was also an increase in the incidence of local secondary tumor foci within the pancreas and proximal connective tissue in PKN2^{KO} mice (Figures 5D, 5E, S5C, and S5D) and an increase in the incidence of peritoneal and diaphragm-associated metastatic foci (Figures 5F and 5G). No metastatic secondary tumors were observed within the liver or lungs. Primary tumor invasion into normal pancreatic tissue was statistically enriched in the PKN2^{KO} cohort, with only one limited incidence observed across the WT and HET cohorts (Figures 5H and 5I). In contrast to PKN2^{KO}, heterozygous deletion of stromal PKN2 does not enhance growth or invasion. Finally, growth, invasion, and secondary tumor burden are all statistically enhanced in PKN2^{KO} tumors when compared with mice bearing at least one intact PKN2 allele (PKN2^{WT} and PKN2^{HET}; Figures S5B–S5G). These data indicate that stromal PKN2^{KO} *in vivo* promotes faster growing and more locally invasive pancreatic tumors. This concurs with previous reports where suppression of myofibroblasts in mouse PDAC models can promote, rather than suppress, aggressive PDAC growth (Ozdemir et al., 2014; Rhim et al., 2014). Loss of myofibroblast function thus appears to limit the tumor-restraining function of PSCs to promote a locally advanced pancreatic cancer (LAPC) phenotype (Seufferlein et al., 2019). This is of clinical importance as locally advanced disease, in the absence of distant metastasis, represents a significant proportion of inoperable and fatal PDAC cases (Seufferlein et al., 2019). It remains to be seen whether PKN2-dependent, PSC-led invasion is critical for distant metastasis, where PSCs have been shown to accompany PDAC cells to metastatic sites (Xu et al., 2010).

Enhanced tumor invasion in PKN2^{KO} mice is associated with a pro-metastatic matrisome signature

Sirius Red staining indicated comparable overall levels of collagen content (Figures 5J and 5K) and α-SMA-positive cell content in WT and PKN2^{KO} tumors (Figures 5J and 5L). Importantly, tissue staining for α-SMA does not assess incorporation into stress fibers, and *in vitro*, total expressed α-SMA protein levels are not reduced following PKN2 deletion in PSCs (Figure S1I). Endomucin-positive vessel density was marginally reduced in PKN2^{KO} mice over WT controls (Figures 5J and 5M). To comprehensively assess the effects of stromal PKN2 deletion on ECM components, and more globally on tumor biology, we conducted bulk RNA-sequencing (RNA-seq) analysis of all female PKN2 WT and KO tumors and conducted gene set enrichment analysis (GSEA); we prioritized the female tumors as we had samples for n $\geq$ 5 for WT and KO tumors, and the TB32048 cell line was derived from a female syngeneic mouse. Many of the most significantly upregulated gene sets in PKN2^{KO} mice were those associated with ECM and matrisome signatures (Figure S5H). We next compared the ECM signatures from our cultured PSCs (Figure 2) with the matching data extracted from the orthotopic tumors, which revealed a striking correlation between WT and KO ECM gene expression patterns *in vitro* in PSCs and *in vivo* in tumors (Figure S5I). This gives confidence that the alterations to the tumor matrisome result from deletion of PKN2 in PSCs and CAFs. This is additionally supported by Tian et al., who demonstrate that most ECM and ECM-regulating proteins in orthotopic PDAC tumors are derived from stromal cells and not the malignant epithelium (Tian et al., 2019). Notably, GSEA analysis also indicated upregulation of epithelial-mesenchymal transition (EMT), inflammatory response, and interleukin-6 (IL-6)-STAT3 signaling in bulk RNA-seq data, which concurs with the observed invasive tumor phenotypes and proposed iCAF switching in the PKN2^{KO} cohort (Figure S5J). Finally, we constructed gene sets based on DE genes from the iCAF and myCAF datasets (Öhlund et al., 2017); PKN2 loss was associated with an enriched iCAF signature and diminished myCAF signature in bulk tumor RNA-seq data (Figure S5K). These data also corroborate reports that iCAFs can promote more aggressive and invasive tumor growth with high EMT, STAT3, and inflammatory signatures (Biffi et al., 2019; Shi et al., 2019; Steele et al., 2021). IL-6 staining of tumors also shows an upward trend in PKN2^{KO} tumors (Figure S5L). This invasive phenotype is enhanced despite the fact that PSC-led invasion is likely to be compromised in the absence of PKN2 (Figure 2).

To further explore the impact of PKN2 deletion on the tumor matrisome, we next assessed the pro-metastatic matrix index (MI) defined by Pearce et al. (2018). The MI is calculated from the expression pattern of 22 genes associated with metastasis and poor outcome across multiple tumor types, including pancreatic cancer (Pearce et al., 2018). Tumors isolated from PKN2^{KO} mice exhibited a significantly increased MI relative to PKN2 WT mice (Figures 6A and 6B), which predicts PKN2^{KO} tumors to be more invasive, as we have observed. One WT tumor exhibited high expression of most MI genes, indicating heterogeneity between mice (Figure 6A). Also, while the MI was increased, a number of protective genes from the MI index are upregulated following PKN2^{KO}, indicating distinctions between the MI and PKN2^{KO} matrisome signatures. We also stained tumor sections for the core MI components COMP, FN1, CTSB, and VCAN, which were transcriptionally upregulated in PKN2^{KO} tumors (Figure 6A); stains were enhanced in invasive regions and in regions of connective tissue (Figures 6C, S6A, and S6B). Only one limited region of invasion was identified in the WT and HET cohorts, with insufficient material to stain for MI components; staining of MI

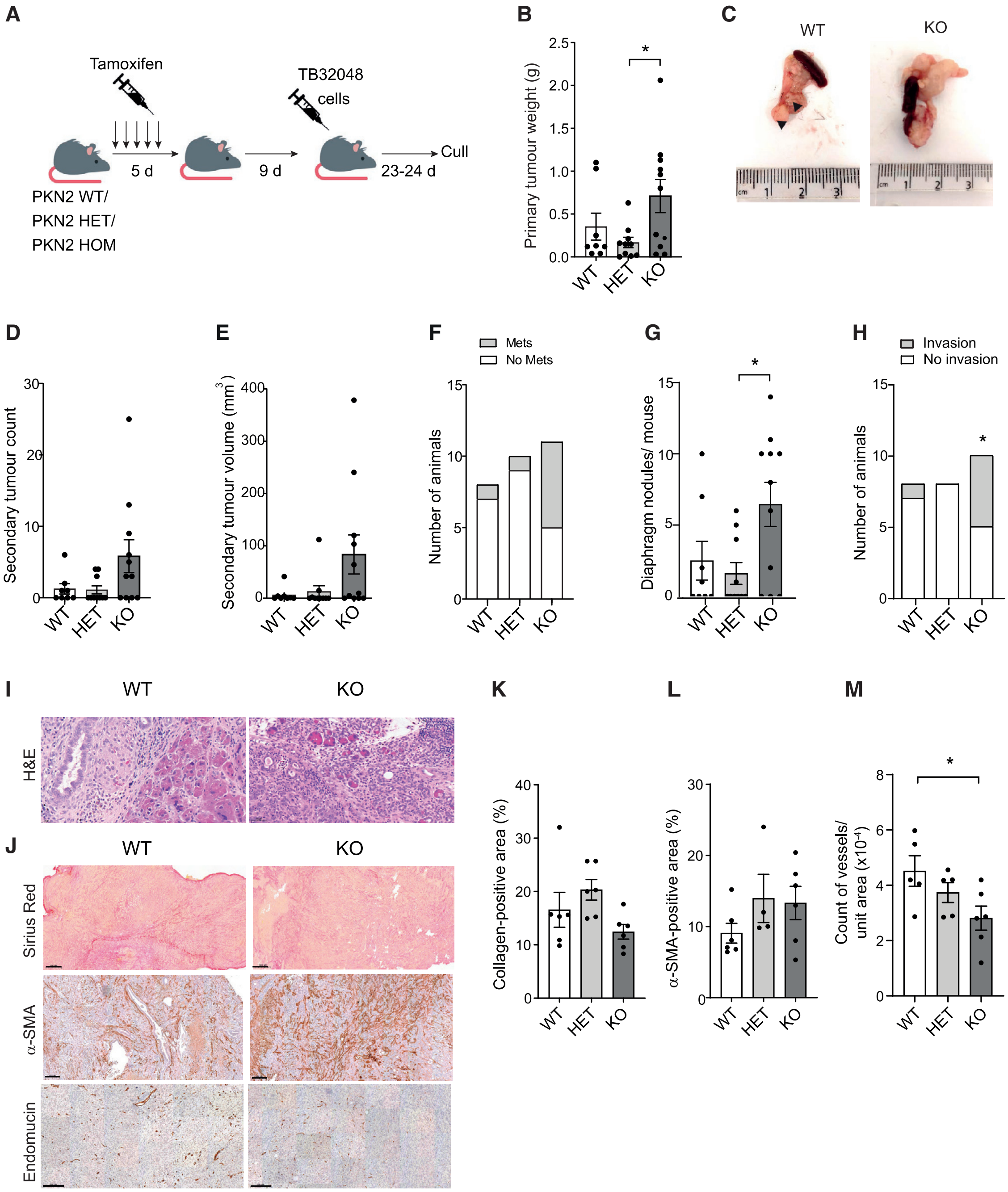

Figure 5. Deletion of stromal PKN2 *in vivo* promotes pancreatic tumor invasion

(A) Schematic of experimental model for orthotopic pancreatic tumor development in inducible conditional $PKN2^{KO}$ mice; Rosa26 CreERT2 was induced with tamoxifen in PKN2 WT, HET, or KO mice; n = 8–11/group; d, days.

(B and C) Quantification of primary tumor volume (B), with representative pictures of tumors alongside spleens (C).

(D–F) Quantification of the number (D) and volume (E) of secondary tumors found associated with the peritoneum and the number of mice with (gray) or without (white) these foci (F).

(G) Quantification of the number of diaphragm nodules found per mouse. $^*p < 0.05$; one-way ANOVA with Sidak's multiple comparison's test.

(H) Quantification of the number of animals with (gray) or without (white) sites of invasion observed in cross-sections of the tumor ($^*p < 0.05$; chi-squared test for distribution of invasive sites across genotypes).

(I) Representative H&E staining of abutted region of tumor with healthy pancreas in the WT (left) and invasive tumor region of a tumor in a $PKN2^{KO}$ mouse (right; scale bar represents 50 μm).

(J–M) Sirius Red (scale bar represents 500 μm), α-SMA (scale bar represents 200 μm), and endomucin (scale bar represents 200 μm) staining (J) of primary tumors with respective quantification of positive stain per pixel area (K and L) or vessel count (M).

(M) $^*p<0.05$; one-way ANOVA with Sidak's multiple comparison's test.

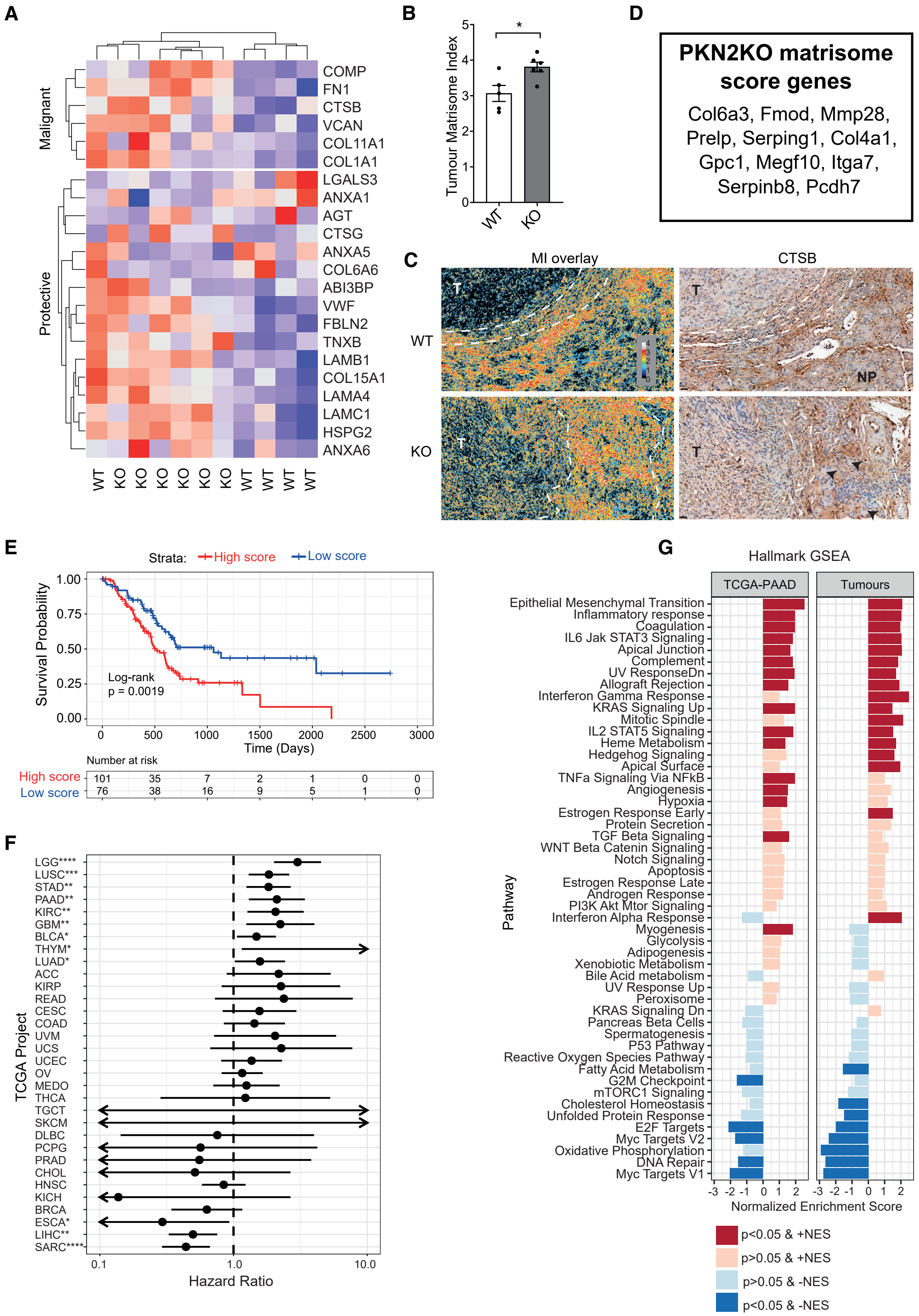

Figure 6. Enhanced tumor invasion in PKN2^{KO} mice is associated with a pro-metastatic matrisome score

(A) Unsupervised clustering of PKN2 WT and KO tumors based on their expression of the 22 MI genes defined by Pearce et al.

(B) MI score of WT and PKN2^{KO} tumors (n = 5–6 tumors/group; *p < 0.05; unpaired t test).

(legend continued on next page)

components in the tumor interior was, however, comparable between genotypes.

To validate our observations in human cancer data, we defined a PKN2-null matrisome signature of statistically significant DE genes from PSCs (Table S1), which concurred with expression in orthotopic tumors (Table S2); we selected the top 11 genes as a high-confidence PKN2^{KO} matrisome gene set (Figure 6D; Table S3). Bulk tumor expression data were then used to generate a PKN2^{KO} matrisome score based on the sum of the *Z* scores of the gene set (Figure 6D). Stromal PKN2^{KO} tumors have a significantly higher PKN2^{KO} matrisome score than WT tumors, as expected (Figure S6C). Next, we used our PKN2^{KO} matrisome score to stratify The Cancer Genome Atlas (TCGA) expression data (Table S4). High PKN2^{KO} matrisome score was associated with poor outcome in pancreatic cancer by Kaplan-Meier (Figure 6E) and multivariate analysis with covariates for age; tumor, node, and metastasis (TNM) staging; and therapy history (Figure 6F; Liu et al., 2018); univariate survival analysis also indicates strong prognostic value for the majority of the individual PKN2^{KO} matrisome genes (Figure S6D). Stratification using the PKN2^{KO} matrisome score did not enrich for any specific pancreatic tumor subtypes defined by Moffit et al. (2015), Bailey et al. (2016), or Collisson et al. (2011; Figures S6E and S6F) or for common PDAC driver mutations (Figure S6G). The PKN2^{KO} matrisome score was prognostic in additional solid tumors, including lung cancers (lung squamous cell carcinoma [LUSC] and lung adenocarcinoma [LUAD]) and gliomas (glioblastoma multiforme [GBM]; Figure 6F). Finally, we used GSEA to compare stratified TCGA-pancreatic adenocarcinoma (PAAD) (PDAC) expression data with our orthotopic tumor dataset. This revealed almost identical phenotypic patterns across a broad set of tumor phenotypes; stromal PKN2 loss (orthotopics) or a high PKN2^{KO} matrisome score (TCGA) is associated with high tumor EMT, inflammation, and IL-6-Jak-STAT3 and KRAS signaling, alongside a reduction in Myc targets, DNA repair, and oxidative phosphorylation (Figure 6G). These data suggest that stromal changes to the matrisome associated with stromal PKN2 loss are not favorable in pancreatic and other solid cancers and identify a novel stromal intervention, which can dictate tumor phenotype.

DISCUSSION

We have identified PKN2 as a novel regulator of PSC phenotype. Deletion of PKN2 results in a loss of myofibroblast features, inducing a switch toward a secretory iCAF phenotype and driving significant pro-tumorigenic alterations to matrisome and inflammatory expression signatures. Interestingly, *in vivo*, deletion of stromal PKN2 resulted in more invasive pancreatic tumors, in agreement with studies where myofibroblast phenotype CAFs have been suppressed or ablated (Ozdemir et al., 2014; Rhim et al., 2014). This concurs with studies from the Tuveson lab proposing that myCAF populations can restrain pancreatic tumor growth while iCAFs drive aggressive inflammatory tumors (Biffi et al., 2019). Our study adds weight to the growing understanding that CAFs exist in interconvertible states, which can be manipulated to modify tumor phenotypes, with potential to modify therapy response (Biffi et al., 2019; Grauel et al., 2020; Hutton et al., 2021; Steele et al., 2021). Importantly, our work shows for the first time that suppressing myofibroblast features by targeting a Rho effector and mechanotransduction is sufficient to trigger iCAF reprogramming.

The importance of the cancer matrisome as a prognostic indicator was recently examined by Pearce et al., who defined a MI associated with ovarian cancer metastasis, which predicts outcome in many solid malignancies (Pearce et al., 2018). Here, we derived a PKN2^{KO} matrisome signature score that also predicts poor outcome in many solid tumor types, including pancreatic cancer. In contrast to the MI, our PKN2^{KO} score predicted outcome for high- and low-grade gliomas and prognostic power differed for several other tumor types. Notably, for some cancers, including sarcomas and hepatocellular carcinoma, a high PKN2^{KO} matrisome score was associated with better outcome, suggesting that, in selected contexts, targeting PKN2 in the stroma may be beneficial.

Targeting PSC function and stromal fibrosis to modulate PDAC disease course and improve therapy responses has yielded mixed and often conflicting results. As examples, targeting the Hedgehog pathway or FAK has been shown to reduce desmoplasia and enhance therapy responses, while separate reports indicate intervention in the same pathways promotes more aggressive PDAC tumors (Demircioglu et al., 2020; Jiang et al., 2016; Lee et al., 2014; Olive et al., 2009; Rhim et al., 2014). Suppression of fibrosis through deletion of Col1a1 from α-SMA^{+} myofibroblasts has also been recently shown to accelerate pancreatic tumor growth (Chen et al., 2021). Stromal reprogramming with the vitamin A analogue, ATRA, or the vitamin D receptor agonist calcipotriol has shown promise, with both approaches promoting a quiescent PSC phenotype, reduced tumor fibrosis, and enhanced chemotherapy responses (Carapuca et al., 2016; Froeling et al., 2011; Kocher et al., 2020; Sherman et al., 2014). Interestingly, PKN2 loss was also associated with enhanced lipid droplet accumulation in PSCs *in vitro*, a key marker of quiescence, although no reduction in fibrosis was observed in tumors; in contrast to ATRA, our data support a switch in CAF phenotype as opposed to adoption of quiescence *in vivo*. These conflicting

(C) Pseudocolor overlay of MI ECM proteins VCAN, FN1, COMP, and CTSB at the edge or invasive front of tumors in a representative PKN2 WT (top) or PKN2^{KO} tumor (bottom, left panels). Cathepsin B staining (right panels) of tissue sections used in the overlay is shown; calibration bar in overlay indicates the number of overlapping ECM proteins at each pixel (T, tumor; NP and arrows indicate healthy pancreatic acini; area within white dotted lines indicates edge or invasive area).

(D) PKN2^{KO} matrisome signature genes based on high-confidence PSC and orthotopic DE gene set.

(E) Kaplan-Meier analysis of TCGA-PAAD patients with high (red) or low (blue) expression of PKN2^{KO} matrisome score.

(F) Hazard ratio (HR) scores with 95% confidence interval (CI) determined by multivariate Cox proportional hazards model across TCGA tumor datasets. HR > 1; high PKN2^{KO} matrisome score associated with poor prognosis. *p < 0.05, **p < 0.01, ***p < 0.001, and ****p < 0.0001.

(G) Hallmark GSEA analysis of RNA-seq data from TCGA-PAAD stratified PKN2^{KO} matrisome score compared with WT versus PKN2^{KO} orthotopic tumors.

studies likely reflect the tumor-suppressive roles of the matrisome and myofibroblasts, which co-exist with the less desirable effects of limiting therapy response. Our data highlight that targeting specific CAF functions, such as myofibroblast contractility, may induce a switch in transcriptional profiles toward distinct CAF subtypes with potentially significant impact on prognosis.

Encouragingly, we have evidence that PKN2 may regulate the contractile motile myofibroblast phenotype in distinct mesenchymal models. Both MEFs and PSCs show a dependence on PKN2 for growth and invasion, in both cases sharing YAP as a common effector. Further, during development, neural crest cells fail to migrate in PKN2^{KO} embryos, suggesting emerging dependence on PKN2 post-EMT, with implications for cancer cell invasion (Quetier et al., 2016). Indeed, we recently contributed to work identifying that PKN2 and ROCK1 collaborate to mediate rear end retraction in durotaxis (Hetmanski et al., 2019), focusing on mesenchymal migratory cancer cell models, a process which also requires mechanical activation of YAP (Lachowski et al., 2018). Defining how PKN2 modulates heterogeneous mesenchymal populations in diverse settings represents a key next challenge, which will be aided through the development of selective PKN2 inhibitors.

Together, our data identify PKN2 as a potential target to modulate the pathological activation of fibroblasts. However, preventing fibroblast activation could also suppress the ability of myofibroblasts to contain and suppress malignant tumor growth by altering the fibroblast matrisome and secretome. The fibrotic, hypovascular nature of the pancreatic cancer stroma nonetheless remains a critical barrier to both chemo- and immunotherapy. Targeting fibrosis to improve therapy responses while retaining the tumor-suppressive functions of fibroblasts thus presents a clinical dilemma.

Limitations of the study

Challenges with orthotopic and genetic models to deconvolute PKN2 function in tumors

While our data provide further support for myofibroblast CAFs in a tumor-restraining role, invasive CAF subtypes may remain important for distant lymphatic or hematogenous metastasis. Indeed, PSCs have been reported to accompany PDAC cells to metastatic sites (Xu et al., 2010), although this does not provide causative evidence. The orthotopic model employed in our study does not metastasize to either the liver or lung across the time course examined. Additional orthotopic models with metastatic potential could be used to address this. Targeting stromal PKN2 in a genetic metastatic PDAC model would provide an alternative albeit complex multi-locus model; targeting PKN2 through Cre-Lox recombination would necessitate pancreatic tumor induction through a non-Cre-driven model, such as the KPF mouse (Schonhuber et al., 2014).

As an additional caveat, our Rosa26-CreERT2 model targets PKN2 systemically throughout the stroma and normal pancreas. While we present evidence that this results in CAF phenotypic switching in tumors, we cannot rule out the impact of PKN2 deletion on other cells in the tumor microenvironment (TME). Additional cell-type-specific Cre models will be needed to address this limitation.

Development of PKN2 selective inhibitors, which do not exhibit the confounding off-target effects of currently available compounds, such as Y27632, Fasudil, and PKC412, would provide a pharmacological route for addressing specific PKN2 roles in tumor biology (Falk et al., 2014). Encouragingly, these non-selective inhibitors are known to suppress PSC myofibroblast function (Masamune et al., 2003; Whatcott et al., 2017) and show promising pre-clinical activity in mouse PDAC models (El Fitori et al., 2007; Vennin et al., 2017, 2020; Whatcott et al., 2017), where PKN2 is likely to have roles in both stromal and tumor compartments; the contribution of PKN2 targeting to the *in vivo* effects of these inhibitors remains to be addressed. Inhibiting the invasive capacity of mesenchymal stromal and cancer cells, with concomitant switching of CAFs toward an inflammatory phenotype, may yet prove to have significant beneficial impact when combined with chemotherapy or immunotherapy.

STAR★METHODS

Detailed methods are provided in the online version of this paper and include the following:

- KEY RESOURCES TABLE
- RESOURCE AVAILABILITY
  - Lead contact
  - Materials availability
  - Data and code availability
- EXPERIMENTAL MODELS
  - Cell lines
  - Mice
- METHOD DETAILS
  - MTT staining
  - Growth assay
  - Western blotting
  - Oil red O staining
  - Cell cycle analysis
  - Collagen gel contraction assay
  - QIAseq targeted RNA expression analysis
  - Reporter assays
  - Nuclear localisation and cell size analysis
  - qPCR analysis
  - Spheroid 3D co-cultures
  - RNA sequencing and analysis
  - Bioinformatics
  - siRNA transfection
  - Immunohistochemistry
  - *In vivo* tumour experiment
- QUANTIFICATION AND STATISTICAL ANALYSIS

SUPPLEMENTAL INFORMATION

Supplemental information can be found online at https://doi.org/10.1016/j.celrep.2021.110227.

ACKNOWLEDGMENTS

We thank Core Services at Barts Cancer Institute, including the Histopathology, Microscopy, Flow Cytometry, *In Vivo* Imaging, and Animal Technical Services. We also thank Ms. Eva Wozniak and Dr. Charles Mein for QIAseq

NGS (Barts and the London Genome Center, QMUL). Finally, thanks to the BRF and histopathology unit at the Francis Crick Institute, who supported mouse models and cell line derivation. This research was funded by Worldwide Cancer Research/Pancreatic Cancer Research Fund (18-0713), Pancreatic Cancer UK (PCUK2015_A26_Cameron), the Academy of Medical Sciences (SBF001\1004), The Royal Society (RG140568), Barts Charity (MGU0605), and Cancer Research UK Center Grants to Barts Cancer Institute (C355/A25137) and the City of London Centre (C7893/A26233); E.R.M. and J.C.H. were funded by Cancer Research UK studentships (C16420/A20916 and C355/A29277) and the Rosetrees Trust (M483). J.L.W. is supported by a BBSRC/AstraZeneca iCASE/LIDo studentship, and P.B. was supported by an MRC studentship to QMUL.

AUTHOR CONTRIBUTIONS

E.R.M. and S.M. performed the majority of the experiments; J.L.W., L.A.C., P.B., I.Q., A.D., C.S.R., N.S., and A.J.M.C. contributed cell-based experiments; J.C.H. conducted bioinformatic, image, and data analysis; H.M.K., P.K., E.J.T., and O.P. assessed tumor pathology and matrix signatures; V.R. and P.C. performed mass spectrometry analysis; E.P.C. and R.P.G. supported 3D model development; F.K., J.L.W., and J.C.H. analyzed RNA-seq data; A.J.M.C., I.R., J.J.T.M., M.T., and K.H.-D. supported *in vivo* studies and mouse model development; J.F.M. and S.M. supported project development; and A.J.M.C., E.R.M., and S.M. conceived and developed the study and wrote the manuscript, with comments and approval from all the authors.

DECLARATION OF INTERESTS

The authors declare no competing interests.

INCLUSION AND DIVERSITY

We worked to ensure sex balance in the selection of non-human subjects. We worked to ensure diversity in experimental samples through the selection of the genomic datasets. One or more of the authors of this paper self-identifies as a member of the LGBTQ+ community.

Received: March 23, 2021
Revised: October 18, 2021
Accepted: December 15, 2021
Published: January 25, 2022

REFERENCES

Anders, S., Pyl, P.T., and Huber, W. (2015). HTSeq–a Python framework to work with high-throughput sequencing data. Bioinformatics *31*, 166–169. https://doi.org/10.1093/bioinformatics/btu638.

Apte, M.V., Haber, P.S., Applegate, T.L., Norton, I.D., McCaughan, G.W., Korsten, M.A., Pirola, R.C., and Wilson, J.S. (1998). Periacinar stellate shaped cells in rat pancreas: identification, isolation, and culture. Gut *43*, 128–133.

Bachem, M.G., Schneider, E., Gross, H., Weidenbach, H., Schmid, R.M., Menke, A., Siech, M., Beger, H., Grunert, A., and Adler, G. (1998). Identification, culture, and characterization of pancreatic stellate cells in rats and humans. Gastroenterology *115*, 421–432.

Bailey, P., Chang, D.K., Nones, K., Johns, A.L., Patch, A.M., Gingras, M.C., Miller, D.K., Christ, A.N., Bruxner, T.J., Quinn, M.C., et al. (2016). Genomic analyses identify molecular subtypes of pancreatic cancer. Nature *531*, 47–52. https://doi.org/10.1038/nature16965.

Bankhead, P., Loughrey, M.B., Fernandez, J.A., Dombrowski, Y., McArt, D.G., Dunne, P.D., McQuaid, S., Gray, R.T., Murray, L.J., Coleman, H.G., et al. (2017). QuPath: open source software for digital pathology image analysis. Sci. Rep. *7*, 16878. https://doi.org/10.1038/s41598-017-17204-5.

Biffi, G., Oni, T.E., Spielman, B., Hao, Y., Elyada, E., Park, Y., Preall, J., and Tuveson, D.A. (2019). IL1-Induced JAK/STAT signaling is antagonized by TGFbeta to shape CAF heterogeneity in pancreatic ductal adenocarcinoma. Cancer Discov. *9*, 282–301. https://doi.org/10.1158/2159-8290.CD-18-0710.

Buchholz, M., Biebl, A., Neesse, A., Wagner, M., Iwamura, T., Leder, G., Adler, G., and Gress, T.M. (2003). SERPINE2 (protease nexin I) promotes extracellular matrix production and local invasion of pancreatic tumors in vivo. Cancer Res. *63*, 4945–4951.

Calvo, F., Ege, N., Grande-Garcia, A., Hooper, S., Jenkins, R.P., Chaudhry, S.I., Harrington, K., Williamson, P., Moeendarbary, E., Charras, G., and Sahai, E. (2013). Mechanotransduction and YAP-dependent matrix remodelling is required for the generation and maintenance of cancer-associated fibroblasts. Nat. Cell Biol. *15*, 637–646. https://doi.org/10.1038/ncb2756.

Carapuca, E.F., Gemenetzidis, E., Feig, C., Bapiro, T.E., Williams, M.D., Wilson, A.S., Delvecchio, F.R., Arumugam, P., Grose, R.P., Lemoine, N.R., et al. (2016). Anti-stromal treatment together with chemotherapy targets multiple signalling pathways in pancreatic adenocarcinoma. J. Pathol. *239*, 286–296. https://doi.org/10.1002/path.4727.

Carpenter, A.E., Jones, T.R., Lamprecht, M.R., Clarke, C., Kang, I.H., Friman, O., Guertin, D.A., Chang, J.H., Lindquist, R.A., Moffat, J., et al. (2006). CellProfiler: image analysis software for identifying and quantifying cell phenotypes. Genome Biol. *7*, R100. https://doi.org/10.1186/gb-2006-7-10-r100.

Chen, Y., Kim, J., Yang, S., Wang, H., Wu, C.J., Sugimoto, H., LeBleu, V.S., and Kalluri, R. (2021). Type I collagen deletion in alphaSMA(+) myofibroblasts augments immune suppression and accelerates progression of pancreatic cancer. Cancer Cell *39*, 548–565.e6. https://doi.org/10.1016/j.ccell.2021.02.007.

Collisson, E.A., Sadanandam, A., Olson, P., Gibb, W.J., Truitt, M., Gu, S., Cooc, J., Weinkle, J., Kim, G.E., Jakkula, L., et al. (2011). Subtypes of pancreatic ductal adenocarcinoma and their differing responses to therapy. Nat. Med. *17*, 500–503. https://doi.org/10.1038/nm.2344.

Cotsiki, M., Lock, R.L., Cheng, Y., Williams, G.L., Zhao, J., Perera, D., Freire, R., Entwistle, A., Golemis, E.A., Roberts, T.M., et al. (2004). Simian virus 40 large T antigen targets the spindle assembly checkpoint protein Bub1. Proc. Natl. Acad. Sci. U S A *101*, 947–952. https://doi.org/10.1073/pnas.0308006100.

Crider, B.J., Risinger, G.M., Jr., Haaksma, C.J., Howard, E.W., and Tomasek, J.J. (2011). Myocardin-related transcription factors A and B are key regulators of TGF-beta1-induced fibroblast to myofibroblast differentiation. J. Invest. Dermatol. *131*, 2378–2385. https://doi.org/10.1038/jid.2011.219.

Danno, S., Kubouchi, K., Mehruba, M., Abe, M., Natsume, R., Sakimura, K., Eguchi, S., Oka, M., Hirashima, M., Yasuda, H., and Mukai, H. (2017). PKN2 is essential for mouse embryonic development and proliferation of mouse fibroblasts. Genes Cell *22*, 220–236. https://doi.org/10.1111/gtc.12470.

Demircioglu, F., Wang, J., Candido, J., Costa, A.S.H., Casado, P., de Luxan Delgado, B., Reynolds, L.E., Gomez-Escudero, J., Newport, E., Rajeeve, V., et al. (2020). Cancer associated fibroblast FAK regulates malignant cell metabolism. Nat. Commun. *11*, 1290. https://doi.org/10.1038/s41467-020-15104-3.

Dennler, S., Itoh, S., Vivien, D., ten Dijke, P., Huet, S., and Gauthier, J.M. (1998). Direct binding of Smad3 and Smad4 to critical TGF beta-inducible elements in the promoter of human plasminogen activator inhibitor-type 1 gene. EMBO J. *17*, 3091–3100. https://doi.org/10.1093/emboj/17.11.3091.

Djurec, M., Grana, O., Lee, A., Troule, K., Espinet, E., Cabras, L., Navas, C., Blasco, M.T., Martin-Diaz, L., Burdiel, M., et al. (2018). Saa3 is a key mediator of the protumorigenic properties of cancer-associated fibroblasts in pancreatic tumors. Proc. Natl. Acad. Sci. U S A *115*, E1147–E1156. https://doi.org/10.1073/pnas.1717802115.

Drifka, C.R., Loeffler, A.G., Esquibel, C.R., Weber, S.M., Eliceiri, K.W., and Kao, W.J. (2016). Human pancreatic stellate cells modulate 3D collagen alignment to promote the migration of pancreatic ductal adenocarcinoma cells. Biomed. Microdevices *18*, 105. https://doi.org/10.1007/s10544-016-0128-1.

Dupont, S., Morsut, L., Aragona, M., Enzo, E., Giulitti, S., Cordenonsi, M., Zanconato, F., Le Digabel, J., Forcato, M., Bicciato, S., et al. (2011). Role of YAP/TAZ in mechanotransduction. Nature *474*, 179–183. https://doi.org/10.1038/nature10137.

Ege, N., Dowbaj, A.M., Jiang, M., Howell, M., Hooper, S., Foster, C., Jenkins, R.P., and Sahai, E. (2018). Quantitative analysis reveals that actin and src-

family kinases regulate nuclear YAP1 and its export. Cell Syst. *6*, 692–708.e13. https://doi.org/10.1016/j.cels.2018.05.006.

Eguchi, D., Ikenaga, N., Ohuchida, K., Kozono, S., Cui, L., Fujiwara, K., Fujino, M., Ohtsuka, T., Mizumoto, K., and Tanaka, M. (2013). Hypoxia enhances the interaction between pancreatic stellate cells and cancer cells via increased secretion of connective tissue growth factor. J. Surg. Res. *181*, 225–233. https://doi.org/10.1016/j.jss.2012.06.051.

El Fitori, J., Su, Y., Buchler, P., Ludwig, R., Giese, N.A., Buchler, M.W., Quentmeier, H., Hines, O.J., Herr, I., and Friess, H. (2007). PKC 412 small-molecule tyrosine kinase inhibitor: single-compound therapy for pancreatic cancer. Cancer *110*, 1457–1468. https://doi.org/10.1002/cncr.22931.

Elyada, E., Bolisetty, M., Laise, P., Flynn, W.F., Courtois, E.T., Burkhart, R.A., Teinor, J.A., Belleau, P., Biffi, G., Lucito, M.S., et al. (2019). Cross-species single-cell analysis of pancreatic ductal adenocarcinoma reveals antigen-presenting cancer-associated fibroblasts. Cancer Discov. *9*, 1102–1123. https://doi.org/10.1158/2159-8290.CD-19-0094.

Falk, M.D., Liu, W., Bolanos, B., Unsal-Kacmaz, K., Klippel, A., Grant, S., Brooun, A., and Timofeevski, S. (2014). Enzyme kinetics and distinct modulation of the protein kinase N family of kinases by lipid activators and small molecule inhibitors. Biosci. Rep. https://doi.org/10.1042/BSR20140010.

Foster, C.T., Gualdrini, F., and Treisman, R. (2017). Mutual dependence of the MRTF-SRF and YAP-TEAD pathways in cancer-associated fibroblasts is indirect and mediated by cytoskeletal dynamics. Genes Dev. *31*, 2361–2375. https://doi.org/10.1101/gad.304501.117.

Froeling, F.E., Feig, C., Chelala, C., Dobson, R., Mein, C.E., Tuveson, D.A., Clevers, H., Hart, I.R., and Kocher, H.M. (2011). Retinoic acid-induced pancreatic stellate cell quiescence reduces paracrine Wnt-beta-catenin signaling to slow tumor progression. Gastroenterology *141*, 1486–1497. https://doi.org/10.1053/j.gastro.2011.06.047.

Froeling, F.E., and Kocher, H.M. (2015). Homeostatic restoration of desmoplastic stroma rather than its ablation slows pancreatic cancer progression. Gastroenterology *148*, 849–850. https://doi.org/10.1053/j.gastro.2015.02.043.

Grauel, A.L., Nguyen, B., Ruddy, D., Laszewski, T., Schwartz, S., Chang, J., Chen, J., Piquet, M., Pelletier, M., Yan, Z., et al. (2020). TGFbeta-blockade uncovers stromal plasticity in tumors by revealing the existence of a subset of interferon-licensed fibroblasts. Nat. Commun. *11*, 6315. https://doi.org/10.1038/s41467-020-19920-5.

Heinrich, E.L., Arrington, A.K., Ko, M.E., Luu, C., Lee, W., Lu, J., and Kim, J. (2013). Paracrine activation of chemokine receptor CCR9 enhances the invasiveness of pancreatic cancer cells. Cancer Microenviron. *6*, 241–245. https://doi.org/10.1007/s12307-013-0130-6.

Hetmanski, J.H.R., de Belly, H., Busnelli, I., Waring, T., Nair, R.V., Sokleva, V., Dobre, O., Cameron, A., Gauthier, N., Lamaze, C., et al. (2019). Membrane tension orchestrates rear retraction in matrix-directed cell migration. Dev. Cell. https://doi.org/10.1016/j.devcel.2019.09.006.

Hingorani, S.R., Wang, L., Multani, A.S., Combs, C., Deramaudt, T.B., Hruban, R.H., Rustgi, A.K., Chang, S., and Tuveson, D.A. (2005). Trp53R172H and KrasG12D cooperate to promote chromosomal instability and widely metastatic pancreatic ductal adenocarcinoma in mice. Cancer Cell *7*, 469–483. https://doi.org/10.1016/j.ccr.2005.04.023.

Hinz, B., Celetta, G., Tomasek, J.J., Gabbiani, G., and Chaponnier, C. (2001). Alpha-smooth muscle actin expression upregulates fibroblast contractile activity. Mol. Biol. Cell *12*, 2730–2741.

Hutton, C., Heider, F., Blanco-Gomez, A., Banyard, A., Kononov, A., Zhang, X., Karim, S., Paulus-Hock, V., Watt, D., Steele, N., et al. (2021). Single-cell analysis defines a pancreatic fibroblast lineage that supports anti-tumor immunity. Cancer Cell. https://doi.org/10.1016/j.ccell.2021.06.017.

Jiang, H., Hegde, S., Knolhoff, B.L., Zhu, Y., Herndon, J.M., Meyer, M.A., Nywening, T.M., Hawkins, W.G., Shapiro, I.M., Weaver, D.T., et al. (2016). Targeting focal adhesion kinase renders pancreatic cancers responsive to checkpoint immunotherapy. Nat. Med. *22*, 851–860. https://doi.org/10.1038/nm.4123.

Kim, D., Langmead, B., and Salzberg, S.L. (2015). HISAT: a fast spliced aligner with low memory requirements. Nat. Methods *12*, 357–360. https://doi.org/10.1038/nmeth.3317.

Kocher, H.M., Basu, B., Froeling, F.E.M., Sarker, D., Slater, S., Carlin, D., deSouza, N.M., De Paepe, K.N., Goulart, M.R., Hughes, C., et al. (2020). Phase I clinical trial repurposing all-trans retinoic acid as a stromal targeting agent for pancreatic cancer. Nat. Commun. *11*, 4841. https://doi.org/10.1038/s41467-020-18636-w.

Kozono, S., Ohuchida, K., Eguchi, D., Ikenaga, N., Fujiwara, K., Cui, L., Mizumoto, K., and Tanaka, M. (2013). Pirfenidone inhibits pancreatic cancer desmoplasia by regulating stellate cells. Cancer Res. *73*, 2345–2356. https://doi.org/10.1158/0008-5472.CAN-12-3180.

Lachowski, D., Cortes, E., Robinson, B., Rice, A., Rombouts, K., and Del Rio Hernandez, A.E. (2018). FAK controls the mechanical activation of YAP, a transcriptional regulator required for durotaxis. FASEB J. *32*, 1099–1107. https://doi.org/10.1096/fj.201700721R.

Lee, J.J., Perera, R.M., Wang, H., Wu, D.C., Liu, X.S., Han, S., Fitamant, J., Jones, P.D., Ghanta, K.S., Kawano, S., et al. (2014). Stromal response to Hedgehog signaling restrains pancreatic cancer progression. Proc. Natl. Acad. Sci. U S A *111*, E3091–E3100. https://doi.org/10.1073/pnas.1411679111.

Leung, B.M., Lesher-Perez, S.C., Matsuoka, T., Moraes, C., and Takayama, S. (2015). Media additives to promote spheroid circularity and compactness in hanging drop platform. Biomater. Sci. *3*, 336–344. https://doi.org/10.1039/c4bm00319e.

Liu, J., Lichtenberg, T., Hoadley, K.A., Poisson, L.M., Lazar, A.J., Cherniack, A.D., Kovatich, A.J., Benz, C.C., Levine, D.A., Lee, A.V., et al. (2018). An integrated TCGA pan-cancer clinical data resource to drive high-quality survival outcome analytics. Cell *173*, 400–416.e11. https://doi.org/10.1016/j.cell.2018.02.052.

Liu, N., Zhong, L., Ni, G., Lin, J., Xie, L., Li, T., Dan, H., and Chen, Q. (2021). High matrix metalloproteinase 28 expression is associated with poor prognosis in pancreatic adenocarcinoma. OncoTargets Ther. *14*, 4391–4406. https://doi.org/10.2147/OTT.S309576.

Love, M.I., Huber, W., and Anders, S. (2014). Moderated estimation of fold change and dispersion for RNA-seq data with DESeq2. Genome Biol. *15*, 550. https://doi.org/10.1186/s13059-014-0550-8.

Mahoney, W.M., Jr., Hong, J.H., Yaffe, M.B., and Farrance, I.K. (2005). The transcriptional co-activator TAZ interacts differentially with transcriptional enhancer factor-1 (TEF-1) family members. Biochem. J. *388*, 217–225. https://doi.org/10.1042/BJ20041434.

Masamune, A., Kikuta, K., Satoh, M., Satoh, K., and Shimosegawa, T. (2003). Rho kinase inhibitors block activation of pancreatic stellate cells. Br. J. Pharmacol. *140*, 1292–1302. https://doi.org/10.1038/sj.bjp.0705551.

Masuda, T., Nakashima, T., Namba, M., Yamaguchi, K., Sakamoto, S., Horimasu, Y., Miyamoto, S., Iwamoto, H., Fujitaka, K., Miyata, Y., et al. (2019). Inhibition of PAI-1 limits chemotherapy resistance in lung cancer through suppressing myofibroblast characteristics of cancer-associated fibroblasts. J. Cell Mol. Med. *23*, 2984–2994. https://doi.org/10.1111/jcmm.14205.

Moffitt, R.A., Marayati, R., Flate, E.L., Volmar, K.E., Loeza, S.G., Hoadley, K.A., Rashid, N.U., Williams, L.A., Eaton, S.C., Chung, A.H., et al. (2015). Virtual microdissection identifies distinct tumor- and stroma-specific subtypes of pancreatic ductal adenocarcinoma. Nat. Genet. *47*, 1168–1178. https://doi.org/10.1038/ng.3398.

Neuzillet, C., Tijeras-Raballand, A., Ragulan, C., Cros, J., Patil, Y., Martinet, M., Erkan, M., Kleeff, J., Wilson, J., Apte, M., et al. (2019). Inter- and intra-tumoural heterogeneity in cancer-associated fibroblasts of human pancreatic ductal adenocarcinoma. J. Pathol. *248*, 51–65. https://doi.org/10.1002/path.5224.

Öhlund, D., Handly-Santana, A., Biffi, G., Elyada, E., Almeida, A.S., Ponz-Sarvise, M., Corbo, V., Oni, T.E., Hearn, S.A., Lee, E.J., et al. (2017). Distinct populations of inflammatory fibroblasts and myofibroblasts in pancreatic cancer. J. Exp. Med. *214*, 579–596. https://doi.org/10.1084/jem.20162024.

Olive, K.P., Jacobetz, M.A., Davidson, C.J., Gopinathan, A., McIntyre, D., Honess, D., Madhu, B., Goldgraben, M.A., Caldwell, M.E., Allard, D., et al. (2009). Inhibition of Hedgehog signaling enhances delivery of chemotherapy in a mouse model of pancreatic cancer. Science *324*, 1457–1461. https://doi.org/10.1126/science.1171362.

Ota, M., and Sasaki, H. (2008). Mammalian Tead proteins regulate cell proliferation and contact inhibition as transcriptional mediators of Hippo signaling. Development *135*, 4059–4069. https://doi.org/10.1242/dev.027151.

Ozdemir, B.C., Pentcheva-Hoang, T., Carstens, J.L., Zheng, X., Wu, C.C., Simpson, T.R., Laklai, H., Sugimoto, H., Kahlert, C., Novitskiy, S.V., et al. (2014). Depletion of carcinoma-associated fibroblasts and fibrosis induces immunosuppression and accelerates pancreas cancer with reduced survival. Cancer Cell *25*, 719–734. https://doi.org/10.1016/j.ccr.2014.04.005.

Pearce, O.M.T., Delaine-Smith, R.M., Maniati, E., Nichols, S., Wang, J., Bohm, S., Rajeeve, V., Ullah, D., Chakravarty, P., Jones, R.R., et al. (2018). Deconstruction of a metastatic tumor microenvironment reveals a common matrix response in human cancers. Cancer Discov. *8*, 304–319. https://doi.org/10.1158/2159-8290.CD-17-0284.

Peng, J., Sun, B.F., Chen, C.Y., Zhou, J.Y., Chen, Y.S., Chen, H., Liu, L., Huang, D., Jiang, J., Cui, G.S., et al. (2019). Single-cell RNA-seq highlights intra-tumoral heterogeneity and malignant progression in pancreatic ductal adenocarcinoma. Cell Res. *29*, 725–738. https://doi.org/10.1038/s41422-019-0195-y.

Quetier, I., Marshall, J.J., Spencer-Dene, B., Lachmann, S., Casamassima, A., Franco, C., Escuin, S., Worrall, J.T., Baskaran, P., Rajeeve, V., et al. (2016). Knockout of the PKN family of Rho effector kinases reveals a non-redundant role for PKN2 in developmental mesoderm expansion. Cell Rep. *14*, 440–448. https://doi.org/10.1016/j.celrep.2015.12.049.

Rhim, A.D., Oberstein, P.E., Thomas, D.H., Mirek, E.T., Palermo, C.F., Sastra, S.A., Dekleva, E.N., Saunders, T., Becerra, C.P., Tattersall, I.W., et al. (2014). Stromal elements act to restrain, rather than support, pancreatic ductal adenocarcinoma. Cancer Cell *25*, 735–747. https://doi.org/10.1016/j.ccr.2014.04.021.

Robinson, M.D., McCarthy, D.J., and Smyth, G.K. (2010). edgeR: a Bioconductor package for differential expression analysis of digital gene expression data. Bioinformatics *26*, 139–140. https://doi.org/10.1093/bioinformatics/btp616.

Rosenbluh, J., Nijhawan, D., Cox, A.G., Li, X., Neal, J.T., Schafer, E.J., Zack, T.I., Wang, X., Tsherniak, A., Schinzel, A.C., et al. (2012). beta-Catenin-driven cancers require a YAP1 transcriptional complex for survival and tumorigenesis. Cell *151*, 1457–1473. https://doi.org/10.1016/j.cell.2012.11.026.

Samaras, S.E., Almodovar-Garcia, K., Wu, N., Yu, F., and Davidson, J.M. (2015). Global deletion of Ankrd1 results in a wound-healing phenotype associated with dermal fibroblast dysfunction. Am. J. Pathol. *185*, 96–109. https://doi.org/10.1016/j.ajpath.2014.09.018.

Schmidt, A., Durgan, J., Magalhaes, A., and Hall, A. (2007). Rho GTPases regulate PRK2/PKN2 to control entry into mitosis and exit from cytokinesis. EMBO J. *26*, 1624–1636. https://doi.org/10.1038/sj.emboj.7601637.

Schonhuber, N., Seidler, B., Schuck, K., Veltkamp, C., Schachtler, C., Zukowska, M., Eser, S., Feyerabend, T.B., Paul, M.C., Eser, P., et al. (2014). A next-generation dual-recombinase system for time- and host-specific targeting of pancreatic cancer. Nat. Med. *20*, 1340–1347. https://doi.org/10.1038/nm.3646.

Seufferlein, T., Hammel, P., Delpero, J.R., Macarulla, T., Pfeiffer, P., Prager, G.W., Reni, M., Falconi, M., Philip, P.A., and Van Cutsem, E. (2019). Optimizing the management of locally advanced pancreatic cancer with a focus on induction chemotherapy: expert opinion based on a review of current evidence. Cancer Treat. Rev. *77*, 1–10. https://doi.org/10.1016/j.ctrv.2019.05.007.

Sherman, M.H., Yu, R.T., Engle, D.D., Ding, N., Atkins, A.R., Tiriac, H., Collisson, E.A., Connor, F., Van Dyke, T., Kozlov, S., et al. (2014). Vitamin D receptor-mediated stromal reprogramming suppresses pancreatitis and enhances pancreatic cancer therapy. Cell *159*, 80–93. https://doi.org/10.1016/j.cell.2014.08.007.

Shi, Y., Gao, W., Lytle, N.K., Huang, P., Yuan, X., Dann, A.M., Ridinger-Saison, M., DelGiorno, K.E., Antal, C.E., Liang, G., et al. (2019). Targeting LIF-mediated paracrine interaction for pancreatic cancer therapy and monitoring. Nature *569*, 131–135. https://doi.org/10.1038/s41586-019-1130-6.

Small, E.M. (2012). The actin-MRTF-SRF gene regulatory axis and myofibroblast differentiation. J. Cardiovasc. Transl. Res. *5*, 794–804. https://doi.org/10.1007/s12265-012-9397-0.

Spear, S., Candido, J.B., McDermott, J.R., Ghirelli, C., Maniati, E., Beers, S.A., Balkwill, F.R., Kocher, H.M., and Capasso, M. (2019). Discrepancies in the tumor microenvironment of spontaneous and orthotopic murine models of pancreatic cancer uncover a new immunostimulatory phenotype for B cells. Front. Immunol. *10*, 542. https://doi.org/10.3389/fimmu.2019.00542.

Steele, N.G., Biffi, G., Kemp, S.B., Zhang, Y., Drouillard, D., Syu, L., Hao, Y., Oni, T.E., Brosnan, E., Elyada, E., et al. (2021). Inhibition of hedgehog signaling alters fibroblast composition in pancreatic cancer. Clin. Cancer Res. *27*, 2023–2037. https://doi.org/10.1158/1078-0432.CCR-20-3715.

Subramanian, A., Tamayo, P., Mootha, V.K., Mukherjee, S., Ebert, B.L., Gillette, M.A., Paulovich, A., Pomeroy, S.L., Golub, T.R., Lander, E.S., and Mesirov, J.P. (2005). Gene set enrichment analysis: a knowledge-based approach for interpreting genome-wide expression profiles. Proc. Natl. Acad. Sci. U S A *102*, 15545–15550. https://doi.org/10.1073/pnas.0506580102.

Tian, C., Clauser, K.R., Ohlund, D., Rickelt, S., Huang, Y., Gupta, M., Mani, D.R., Carr, S.A., Tuveson, D.A., and Hynes, R.O. (2019). Proteomic analyses of ECM during pancreatic ductal adenocarcinoma progression reveal different contributions by tumor and stromal cells. Proc. Natl. Acad. Sci. U S A *116*, 19609–19618. https://doi.org/10.1073/pnas.1908626116.

Vennin, C., Chin, V.T., Warren, S.C., Lucas, M.C., Herrmann, D., Magenau, A., Melenec, P., Walters, S.N., Del Monte-Nieto, G., Conway, J.R., et al. (2017). Transient tissue priming via ROCK inhibition uncouples pancreatic cancer progression, sensitivity to chemotherapy, and metastasis. Sci. Transl. Med. *9*. https://doi.org/10.1126/scitranslmed.aai8504.

Vennin, C., Rath, N., Pajic, M., Olson, M.F., and Timpson, P. (2020). Targeting ROCK activity to disrupt and prime pancreatic cancer for chemotherapy. Small GTPases *11*, 45–52. https://doi.org/10.1080/21541248.2017.1345712.

Vonlaufen, A., Joshi, S., Qu, C., Phillips, P.A., Xu, Z., Parker, N.R., Toi, C.S., Pirola, R.C., Wilson, J.S., Goldstein, D., and Apte, M.V. (2008). Pancreatic stellate cells: partners in crime with pancreatic cancer cells. Cancer Res. *68*, 2085–2093. https://doi.org/10.1158/0008-5472.CAN-07-2477.

Vonlaufen, A., Phillips, P.A., Yang, L., Xu, Z., Fiala-Beer, E., Zhang, X., Pirola, R.C., Wilson, J.S., and Apte, M.V. (2010). Isolation of quiescent human pancreatic stellate cells: a promising in vitro tool for studies of human pancreatic stellate cell biology. Pancreatology *10*, 434–443. https://doi.org/10.1159/000260900.

Wada, K., Itoga, K., Okano, T., Yonemura, S., and Sasaki, H. (2011). Hippo pathway regulation by cell morphology and stress fibers. Development *138*, 3907–3914. https://doi.org/10.1242/dev.070987.

Ware, M.J., Colbert, K., Keshishian, V., Ho, J., Corr, S.J., Curley, S.A., and Godin, B. (2016). Generation of homogenous three-dimensional pancreatic cancer cell spheroids using an improved hanging drop technique. Tissue Eng. Part C Methods *22*, 312–321. https://doi.org/10.1089/ten.TEC.2015.0280.

Whatcott, C.J., Ng, S., Barrett, M.T., Hostetter, G., Von Hoff, D.D., and Han, H. (2017). Inhibition of ROCK1 kinase modulates both tumor cells and stromal fibroblasts in pancreatic cancer. PLoS One *12*, e0183871. https://doi.org/10.1371/journal.pone.0183871.

Wiechec, E., Magan, M., Matic, N., Ansell-Schultz, A., Kankainen, M., Monni, O., Johansson, A.C., and Roberg, K. (2021). Cancer-associated fibroblasts modulate transcriptional signatures involved in proliferation, differentiation and metastasis in head and neck squamous cell carcinoma. Cancers (Basel) *13*. https://doi.org/10.3390/cancers13133361.

Xu, Z., Vonlaufen, A., Phillips, P.A., Fiala-Beer, E., Zhang, X., Yang, L., Biankin, A.V., Goldstein, D., Pirola, R.C., Wilson, J.S., and Apte, M.V. (2010). Role of

CellPress
OPEN ACCESS

pancreatic stellate cells in pancreatic cancer metastasis. Am. J. Pathol. *177*, 2585–2596. https://doi.org/10.2353/ajpath.2010.090899.

Yang, C.S., Melhuish, T.A., Spencer, A., Ni, L., Hao, Y., Jividen, K., Harris, T.E., Snow, C., Frierson, H.F., Jr., Wotton, D., and Paschal, B.M. (2017). The protein kinase C super-family member PKN is regulated by mTOR and influences differentiation during prostate cancer progression. Prostate *77*, 1452–1467. https://doi.org/10.1002/pros.23400.

Zhang, Z., Li, H., Deng, Y., Schuck, K., Raulefs, S., Maeritz, N., Yu, Y., Hechler, T., Pahl, A., Fernandez-Saiz, V., et al. (2021). AGR2-dependent nuclear import of RNA polymerase II constitutes a specific target of pancreatic ductal adenocarcinoma in the context of wild-type p53. Gastroenterology *161*, 1601–1614.e23. https://doi.org/10.1053/j.gastro.2021.07.030.

Zhao, B., Li, L., Wang, L., Wang, C.Y., Yu, J., and Guan, K.L. (2012). Cell detachment activates the Hippo pathway via cytoskeleton reorganization to induce anoikis. Genes Dev. *26*, 54–68. https://doi.org/10.1101/gad.173435.111.

Zhao, B., Wei, X., Li, W., Udan, R.S., Yang, Q., Kim, J., Xie, J., Ikenoue, T., Yu, J., Li, L., et al. (2007). Inactivation of YAP oncoprotein by the Hippo pathway is involved in cell contact inhibition and tissue growth control. Genes Dev. *21*, 2747–2761. https://doi.org/10.1101/gad.1602907.

STAR★METHODS

KEY RESOURCES TABLE

REAGENT or RESOURCE	SOURCE	IDENTIFIER
Antibodies		
Anti-BrdU (FACS)	Dako	Cat. No.:0744; RRID: AB_10013660; Clone: Bu20A
Anti-α-SMA (IF)	Dako	Cat No.: A2547; RRID: AB_476701 Clone: 1A4
Anti-YAP1 (IF)	Cell Signaling Technology	Cat. No.:14074; RRID:AB_2650491; Clone: D8H1X
anti-GFAP (IF)	Sigma-Aldrich	Cat. No.: G3893; RRID: AB_477010; Clone:G-A-5
Anti-Vimentin (IF)	Sigma- Aldrich	Cat. No.: sc-5565; RRID: AB_793999; Clone: H-84
Phalloidin (F-actin) - A546	Invitrogen	Cat. No.:A22283; RRID: N/A
Anti-CyclinD1 (WB)	Spring Bioscience	Cat. No.:M3040; RRID: AB_1661031; Clone: SP4
Anti-PCNA (WB)	Oncogene	Cat. No.:NA03-200U6; RRID: AB_10681357; Clone: PC10
Anti-HSC70 (WB)	Santa Cruz	Cat. No.:sc-7298; RRID: AB_627761; Clone: B-6
Anti-PKN2 (WB)	R&D Systems	Cat. No.:MAB5686; RRID: AB_2163979; Clone: 509105
Anti-p-YAP (S112) (WB)	Cell signaling Technology	Cat. No.:4911; RRID: AB_2218913; Polyclonal
Anti-YAP (WB)	Cell signaling Technology	Cat. No.:12395; RRID: AB_2797897; Clone: 1A12
Anti-pSMAD2/3 (WB)	Cell signaling Technology	Cat. No.:8828; RRID: AB_2631089; Clone: D27F4
Anti-SMAD2/3 (WB)	BD Transduction Laboratories	Cat. No.:610843; RRID: AB_398162; Clone: Not available
Anti-p-p70S6K (T389) (WB)	Cell Signaling Technology	Cat. No.:9206S; RRID: AB_2285392; Clone: 1A5
Anti-P70 S6K (WB)	Cell Signaling Technology	Cat. No.:9202S; RRID: AB_331676; Polyclonal
Anti-P-ERK 1/2 (WB)	Cell Signaling Technology	Cat. No.:4370; RRID: AB_2315112; Clone: D13.14.4E
Anti-ERK1/2 (WB)	BD Transduction Laboratories	Cat. No.:E17120; RRID: AB_399647; Clone: 20A
Anti-GAPDH (WB)	Santa Cruz	Cat. No.:sc-25778; RRID: AB_10167668; Clone: FL-335
Anti-V5 -FITC (WB)	Bethyl	Cat. No.:A190-119F; RRID: AB_67319; Polyclonal
Anti-α-SMA (WB)	Dako	Cat. No.:M0851; RRID: AB_2223500; Clone: 1A4
Anti-FN1 (IHC)	Sigma	Cat. No.:F3648; RRID: AB_476976; Polyclonal
Anti-COMP (IHC)	Genetex	Cat. No.:GTX14515; RRID: AB_845475; Polyclonal
Anti-CTSB (IHC)	Novus Biological	Cat. No.:NBP-19797; RRID: AB_1641648; Polyclonal
Anti-VCAN (IHC)	Sigma	Cat. No.:HPA004726; RRID: AB_1080561; Polyclonal
Anti-Mouse IgG HRP (IHC)	GE Healthcare	Cat. No.:NXA931; RRID: AB_772209; Polyclonal
Anti-Endomucin (IHC)	Santa Cruz	Cat. No.:sc-65495; RRID: AB_2100037; Clone: V.7C7
Anti-SMAD4 (IF)	Santa Cruz	Cat. No.:sc-7966; RRID: AB_627905; Clone: B-8
Anti-IL6 (IHC)	Cell Signaling Technology	Cat. No.:12912; RRID: AB_2798059; Clone: D5W4V
Anti-Desmin (IF)	Sigma Aldrich	Cat. No.:D1033; RRID: AB_476897; Clone: DE-U-10
Anti-Mouse IgG A488 (Secondary- IF)	Life Technologies	Cat. No.:A11001; RRID: AB_2534069; Polyclonal
Anti-Rabbit IgG A488 (Secondary- IF)	Life Technologies	Cat. No.:A21206; RRID: AB_2535792; Polyclonal
Anti-Rabbit IgG A555 (Secondary- IF)	Life Technologies	Cat. No.:A31572; RRID: AB_162543; Polyclonal
Anti-Rabbit IgG HRP (Secondary -WB)	GE Healthcare	Cat. No.:NA934V; RRID: N/A
Anti-mouse A488 secondary antibody (FACS for BrDU)	Invitrogen	Cat. No.: A11001; RRID: AB_2534069; Polyclonal

(*Continued on next page*)

Continued

REAGENT or RESOURCE	SOURCE	IDENTIFIER
Bacterial and virus strains		
YAP-EV (pLX304)	David Root - Addgene;	Cat. No.:25890; RRID:Addgene_25890; http://n2t.net/addgene:25890
YAP WT (YAP1-V5 in pLX304)	William Hahn - Addgene;	Cat. No.: 42555; RRID:Addgene_42555; http://n2t.net/addgene:42555
YAP S6A (YAP1 (S6A) - V5 in pLX304)	William Hahn - Addgene	Cat.No.: 42562; RRID:Addgene_42562; http://n2t.net/addgene:42562
MRTF-Luciferase (pGL4.34 luc2P/SRF-RE/Hygro)	Promega	Cat. No.: E1350; RRID: N/A
SBE-Luciferase (p-GL3-CAGA-Luciferase)	Prof. Edel O'Toole; Dennler et al., 1998	N/A
TEAD-Luciferase (pGL3-4xGTIIC-49)	Dr. Nic Tapon; Mahoney et al., 2005	N/A
Renilla plasmid (pRL)	Promega	Cat. No.: E2231
pBabeSV40 Large T	Prof. Parmjit Jat; Cotsiki et al., 2004	N/A
Chemicals, peptides, and recombinant proteins		
TGF-beta 1 (recombinant)	Promega	Cat. No.:100-21-10uG
ATRA (All-Trans Retinoic Acid)	Sigma-Aldrich	Cat. No.: R2625
4-OHT (4-Hydroxytamoxifen)	Sigma-Aldrich	Cat. No.: T176
Tamoxifen (used *in vivo*)	Sigma	Cat. No.: T5648
Mowoil	Calbiochem	Cat. No. : 475904
Collagen I (Part of Collagen gels)	Corning	Cat. No.: 354236
10x low-glucose DMEM	Sigma-Aldrich	Cat. No.: D2429
Lipofectamine LTX / Plus reagent	Invitrogen	Cat. No.: 15388-100
PowerUP SYBR Green Master Mix	Life Technologies	Cat. No.: A25776
Turbo DNaseI	Life Technologies	Cat. No.: AM2238
Methyl Cellulose	Sigma-Aldrich	Cat. No.: M7027
Matrigel (used for orthotopic injections *in vivo* and spheroid cultures *in vitro*) (Corning Matrigel Basement Membrane Matrix)	Corning	Cat. No.: 356234; Lot No.: 8057020
Tween 20 (used in PBS-Tween at 0.1%)	Fisher Bioreagents	Cat. No.: BP337-500
DAB (Diaminobenzidene)	Dako	Cat. No.: K3468
4X NuPAGE LDS sample buffer	Novex	Cat. No.: NP008
PageRuler Plus	Thermo Scientific	Cat. No.: 26616
Bis-Tris (4-12%) pre-cast gels	Nusep	Cat. Nos.: NG11-420, NG21-420, NG31-420
Nitrocellulose membranes	GE Healthcare	Cat. No.: 10600008
Tris-Glycine PAGE buffer	Severn Biotech	Cat. No.: 20-6300-10
Luminata Forte	Millipore	Cat. No.: WBLUF0100
Luminata Crescendo	Millipore	Cat. No.: WBLUR0100
1X ReBlot Plus Mild Antibody Stripping Solution	Merck Millipore	Cat. No.: 2502
Fetal bovine serum (FBS for PSCs)	Gibco	Cat. No.: 10500-064
Critical commercial assays		
Qiaseq- Mouse extracellular matrix & cell adhesion molecules targeted RNA panel	Qiagen	Cat. No.: RMM-004Z
Dual-Glo luciferase assay system	Promega	Cat. No.: E2940
Lunascript RT Supermix Kit	Lunascript	Cat. No.:E3010L
Universal Vectastain ABC kit	Vector Laboratories	Cat. No.: PK-6200
Deposited data		
PKN2 stromal KO pancreatic orthotopic tumour RNA sequencing data	This paper	Gene Expression Omnibus: GSE189027

(*Continued on next page*)

Continued

REAGENT or RESOURCE	SOURCE	IDENTIFIER
ECM targeted RNA sequencing data from PKN2 KO or WT PSCs treated with veichle control or TGFβ	This paper	Gene Expression Omnibus: GSE189245
iCAF/myCAF RNA sequencing data	Öhlund et al., 2017	Gene Expression Omnibus: GSE93313
NPF/CAF RNA sequencing data	Djurec et al., 2018	Gene Expression Omnibus: GSE106901
TCGA RNA sequencing data	Genomic Data Commons (GDC);	portal.gdc.cancer.gov/
TCGA clinical data	TCGA Pan-Cancer Clinical Data Resource (TCGA-CDR); Liu et al., 2018; https://doi.org/10.1016/j.cell.2018.02.052	Table S1
GSEA genesets	Molecular Signatures Database (MSigDB) v7.2; https://www.gsea-msigdb.org/gsea/msigdb/	Hallmark (H), C2 Canonical Pathways (CP) collections
ssRNAseq-Mouse	Gene Expression Omnibus; Biffi et al., 2019	Gene Expression Omnibus: GSE114417- Replicate GSM3141422
ssRNAseq: Human	Genome Sequencing Archive; Peng et al., 2019	GSA: CRA001160 – Project code: PRJCA001063
TCGA Moffitt, Collisson, Bailey subtype metadata	Pancreatic Expression Database (PED);	pancreasexpression.org/analytics/cohort/tcga/
**Experimental models: Cell lines**		
PSCs	This paper (Derived from Rosa26-CreER^{T2}PKN2$^{fl/fl}$ mouse)	RRID: N/A
MEFs	Quetier et al., 2016 (Derived from Rosa26-CreER^{T2}PKN2$^{fl/fl}$embryos)	RRID: N/A
TB32048	Gifted by Dr. David Tuveson. (Spear et al., 2019) Obtained from KPC mice	RRID: N/A
R254	Gifted by Dr. Dieter Saur (Obtained from p48$^{Cre/+}$; LSL-Kras$^{G12D/+}$; p53$^{fl/fl}$ mouse)	RRID: N/A
**Experimental models: Organisms/strains**		
Mouse: *Pkn2*$^{fl/fl}$;*Rosa*$^{CreERT2+/-}$ ˙*C57BL/6NA*^{tm1Brd}-*Pkn2*$^{tm1a(KOMP)Wtsi}$; *Gt(ROSA)26Sor*$^{tm1(cre/ERT2)Tyj}$	This paper; Francis Crick Institute (London Research Institute), Quetier et al., 2016	N/A
Mouse: Rose26^{CreERT2}; *Gt(ROSA)26Sor*$^{tm1(cre/ERT2)Tyj}$	Jackson Laboratories (mice)	RRID: IMSR_JAX:008463
Mouse: *Pkn2* $^{fl/fl}$; *Pkn2*$^{tm1a(KOMP)Wtsi}$	KOMP (ES Cells)	RRID: MMRRC_060318-UCD
**Oligonucleotides**		
Oligonucleotides used in qPCR	See Table S1	N/A
Oligonucleotides used in siRNA experiments	See Table S2	N/A
**Software and algorithms**		
CellProfiler v3.1.9	Carpenter et al., 2006	www.cellprofiler.org
QuPath v0.3.0	Bankhead et al., 2017	qupath.github.io/
Visiopharm Version 2019.07.3.7092	Visiopharm	www.visiopharm.com
ImageQuant TL 8.1	Imagequant; GE Healthcare	N/A
R v4.1.1	**R** Foundation for Statistical Computing	www.r-project.org
GraphPad Prism v8.0.1	GraphPad	www.graphpad.com/scientific-software/prism/
Original Code	This paper	https://doi.org/10.5281/zenodo.5719508

RESOURCE AVAILABILITY

Lead contact

Further information and requests for resources and reagents should be directed to the lead contact, Angus Cameron (a.cameron@qmul.ac.uk).

Materials availability

Rosa26^{CreERT2}*PKN2*$^{fl/fl}$ mice were generated by crossing *Rosa26*^{CreERT2} mice (Jackson Laboratory) with *PKN2*$^{fl/fl}$ mice (KO mouse project (KOMP)) at the Charterhouse biological services unit (BSU) at Queen Mary University. These are available from the lead contact upon request subject to MTA.

Data and code availability

- RNA-sequencing data has been deposited at GEO and are publicly available as of the date of publication. Accession numbers are listed in the key resources table. Western blot and microscopy data are available upon request from the lead contact. This paper also analyzes existing, publicly available data. These accession numbers for the datasets are listed in the key resources table.
- All original code has been deposited at Github and is publicly available as of the date of publication. DOIs are listed in the key resources table.
- Any additional information required to reanalyze the data reported in this paper is available from the lead contact upon request.

EXPERIMENTAL MODELS

Cell lines

TB32048 cells were a kind gift from Prof. David Tuveson and R254 cells from Dr Dieter Saur. TB32048 cell line was derived from a female KPC mouse (Hingorani et al., 2005; Spear et al., 2019) within the Tuveson lab. The R254 cells were derived from the tumour of a LSL-Kras$^{G12D/+}$; p53$^{fl/fl}$ mouse (Schonhuber et al., 2014; Zhang et al., 2021). The sex of the R254 cells is unknown. Cell line authentication was not possible on these cell lines as these details have not been published on either cell line. All cancer cell lines were cultured at 37°C and 5% CO_2 in complete DMEM supplemented with 10% FBS (Sigma),100 U/ml penicillin and 0.1 mg/ml streptomycin (Pen-strep). Inducible PKN2 (iPKN2) knockout (KO) MEFs were isolated from *Rosa26*^{CreERT2}*PKN2*$^{fl/fl}$ C57BL/6 mice as previously described (Quetier et al., 2016). Inducible PKN2^{KO} mouse PSCs were isolated from the pancreas of a male *Rosa26*^{CreERT2}*PKN2*$^{fl/fl}$ mouse using a variation of the density centrifugation method (Apte et al., 1998; Bachem et al., 1998; Vonlaufen et al., 2010). Briefly, the pancreas was digested using Collagenase P, 0.1% DNase1 in GBSS for 30 mins at 37°C. The suspension was washed in 0.3% BSA with 0.1% DNase1 in GBSS and stellate cells separated on a Histodenz cushion, washed in 3% FBS in PBS and plated. PSCs were immortalised by transduction with pBabe SV40 large T plasmid (Prof. Parmjit Jat (Cotsiki et al., 2004)). Mouse embryonic fibroblasts (MEFs) were derived as described previously (Quetier et al., 2016); embryos were decapitated and foetal liver was removed prior to trypsin digestion and serial passage in DMEM with 10% FBS. Lines were immortalised using a 3T3 protocol of serial passage and subsequent senescence escape. To induce PKN2 recombination, iPKN2 PSCs and MEFs were treated with 2 μM and 400 nM 4-hydroxytamoxifen (4-OHT) respectively in cell culture medium for 2 h at 37°C. PSCs were used 3-4 days later and MEFs 7 days later, and cultured in DMEM with 10% FBS (Gibco) and pen-strep.

Mice

All mice and procedures were approved by our local animal ethics committee (Queen Mary University of London) and carried out in accordance with the UK Home Office Animal and Scientific Procedures Act 1986. All mice used in experiments were of *Pkn2*$^{fl/fl}$*Rosa*^{CreERT2} strain developed within Charterhouse Campus biological services Unit by crossing *Pkn2*$^{fl/fl}$ (KOMP) mice with *Rosa*^{CreERT2} (Jackson Laboratories- *Gt(ROSA)26Sor*$^{tm1(cre/ERT2)Tyj}$) mice so that all mice were heterozygous for RosaCreERT2 and were either homozygous, heterozygous or wild type for the floxed PKN2 allele. Conditional PKN2 knockout mice were generated as described by (Quetier et al., 2016). To generate PKN2 null mice targeted ES cells were obtained from the KOMP Repository (www.komp.org: Project ID66263 - pkn2 MGI:109211). An independent ES cell clone, G05 (allele: *Pkn2*$^{tm1a(KOMP)Wtsi}$) underwent germline transmission. To convert the PKN2 to a conditional allele, PKN2 heterozygous mice were crossed with a Flp deleter mouse (Tg(CAG-Flpo)1Afst; background C57Bl/6N); to genotype, sense primer PKN2-F2 (5'-GGTTTGGTGACCAGTAAAAACTG-3') was used with a second gene specific antisense primer (PKN2-R2; 5'-CTGAAGACACTTTGAAAAGGATG -3') to generate 489bp and 635bp products for the wt and conditional alleles respectively. Mice were crossed to C57Bl/6J mice for more than 10 generations before being crossed with *Rosa*^{CreERT2} mice. Mice of all genotypes were maintained in grouped housing of no more than 6 mice per IVC unit and were housed together regardless of genotype. They were sex- and age- matched (24 – 28 weeks old) at the time of experiment initiation. Both males and females were used in the study.

METHOD DETAILS

MTT staining

500 PSCs/well were seeded in 200μl medium in a 96-well plate (at least 5 technical replicate wells per condition). After 4 days, the medium was removed and cells were incubated in 100 μl 0.5 mg/ml 3-(4,5-dimethylthiazol-2-yl)-2,5-diphenyltetrazolium (MTT) (Sigma-Aldrich) prepared in cell culture medium for 2h at 37°C. Medium was then removed from wells and formazan crystals were solubilised in 50 μl DMSO. Absorbance was measured at 570 nm using a colourimetric plate reader (Tecan).

Growth assay

PKN WT or KO cells were plated at 0.4 x 10^4 cells/ well of a 6-well plate in 3 technical triplicates per experiment. They were then trypsinized at the indicated time points (days 2, 4, 6, and 8) and counted using a hemocytometer. Counts are normalized to the area of the well.

Western blotting

Unless otherwise stated, whole cell lysates were prepared by placing cells on ice, washing three times in cold PBS, and adding an appropriate volume of sample buffer (3% SDS, 60 mM sucrose, 65 mM Tris, pH 6.8). Lysates were homogenised by passing through a 25G needle with a 1 ml syringe and then centrifuging at 13,000 RPM for 3 minutes. The Bio-Rad DC protein assay kit (Bio-Rad) was used according to the manufacturer's guidelines to calculate the protein concentration for each sample. Samples were then aliquoted and diluted in distilled H_2O as required to generate samples of an equal protein concentration. 4X LDS sample buffer was prepared by adding 100 μl of 1M DTT to 900 μl 4X NuPAGE LDS sample buffer (Novex) for a concentration of 100 mM DTT. An appropriate volume of 4X LDS sample buffer was added to cell lysates. Samples were then heated in a hot block at 95°C for 5 minutes. Sample proteins were resolved by SDS-PAGE alongside PageRuler Plus molecular weight standards (Thermo Scientific) in Bis-Tris (4-12%) pre-cast gels (Nusep) in 1X Tris-Glycine SDS-PAGE running buffer (Severn Biotech). Proteins were electroblotted onto nitrocellulose membranes (GE Healthcare) by the wet transfer method in Tris-Glycine transfer buffer (20% ethanol in 1X Tris-Glycine PAGE buffer, Severn Biotech 20-6300-10) at 120 V for 1h at 4°C. Blots were blocked for 30 minutes in blocking buffer (3% BSA in TBST (0.1% Tween-20, 20 mM Tris, 150 mM NaCl, pH 7.4)) and incubated in primary antibody in blocking buffer overnight at 4°C. The following day, blots were washed in TBST and incubated in secondary antibody diluted in 5% milk TBST for 1h at room temperature. Blots were washed in TBST and developed using Luminata Forte (Millipore) or Crescendo (Millipore) chemiluminescent substrate in an automated chemiluminescent imager (Amersham Imager 600, GE Healthcare). Band intensity was quantified by densitometry analysis in ImageQuant TL 8.1 software (GE Healthcare). For the stripping and re-probing of blots, membranes were washed 3x5 minutes in distilled H2O, incubated with shaking in 1X ReBlot Plus Mild Antibody Stripping Solution (Merck Millipore) for 15 minutes, washed 3x5 mins in TBST, and re-blocked for 30 mins in blocking buffer. Stripping was confirmed by application of chemiluminescent substrate and imaging.

Oil red O staining

2000 PSCs were seeded on glass coverslips and treated daily with 1 μM ATRA (Sigma-Aldrich) or ethanol vehicle control for 3 days. Cells were washed and fixed for 10 min in 10% neutral buffered formalin (NBF) and washed 3 times with PBS. Lipid-containing vesicles were stained with 0.3% Oil Red O (Sigma-Aldrich) in 60% isopropanol for 1 h at room temperature. Coverslips were then washed with distilled water and stained with Mayer's haematoxylin (incubation for 2 mins) and mounted with Mowiol (10% mowoil (Calbiochem), 24% glycerol, 100mM Tris-HCl pH 8.5). Brightfield pictures of each condition were taken with an Axiophot microscope (Zeiss) and analysed using ImageJ. Oil Red O staining was quantified by adjusting colour threshold to specifically detect area of dark red staining. The total number of stained pixels per image was normalised to cell number per field.

Cell cycle analysis

PSCs were passaged at low density in T175 tissue culture flasks and allowed to grow till approximately 50% confluency. Cells were pulsed with 10 μM BrdU (Sigma) for 30 min, Trypsinized, and collected by centrifugation (300g, 5 mins). Cells were washed in Ca^{2+} and Mg^{2+} free PBS (Gibco) and then resuspended in ice-cold 70% ethanol dropwise whilst vortexing and stored at -20°C at least overnight. Cells were incubated sequentially in 1% Triton X-100 PBS (PBST) for 20-30 mins (permeabilization), 2M HCl for 20 mins at room temperature and then washed in PBS-Tween(0.1%). They were then incubated in 0.1M $Na_2B_4O_7$ for 20 min at RT and washed in PBS-T. Samples were stained with 100 μl α-BrdU (Dako, 0744) diluted in PBS-T for 20 mins, washed twice in PBS-T and then incubated in anti-mouse Alexa fluor 488 secondary antibody (Invitrogen) for 20 mins each. RNA was digested (100 μg/ml RNase 37°C for 15 min) and DNA stained with 50 μg/ml propidium iodide (Sigma-Aldrich) prior to flow cytometry (BD Fortessa) and data analysis (FlowJo).

Collagen gel contraction assay

For each condition, PSCs were resuspended to a final concentration of 1 x 10^5 in 400 μl collagen gel (1mg/ml Collagen I (Corning)) with 10x low-glucose DMEM (Sigma-Aldrich) diluted in complete PSC medium, and neutralized with 12.5 μl/ml 1 M NaOH) on ice and seeded in 24-well plates. PSCs were gently mixed without introducing bubbles. Gels were incubated for 2 h at 37°C to set, and then

500 μl PSC medium added above; 24 h later, this was replaced with 5 ng/ml TGF-β1 (Peprotech) or vehicle control (0.1% BSA, 10 mM citrate, pH 3.0) in complete PSC medium. Gels were released from the edges of the well using a needle and brightfield images taken daily. Percentage gel contraction was calculated by measuring the area of each gel using ImageJ and normalizing the well area revealed by contraction with the equation: 1-(gel area/well area) x 100.

QIAseq targeted RNA expression analysis

PSCs were seeded at 7.5 x 10^4 cells in 15 cm dishes in complete PSC medium (at least 3 dishes for WT PSCs and 4 dishes of PKN2KO /condition). 24 h later, plates were spiked with 5 ng/ml TGF-β1 (Peprotech) or vehicle control (0.1% BSA, 10mM citrate, pH 3.0). After 72 h, RNA was harvested using the QIAGEN RNeasy plus kit (Qiagen). 100 ng RNA from each sample was then processed using the QIAseq mouse extracellular matrix & cell adhesion molecules targeted RNA panel (QIAGEN- RMM-004Z) to isolate and amplify a cDNA library of 419 genes associated with the ECM and cell adhesion as per the manufacturer's instructions. Samples were pooled to generate a 12plex cDNA library and sequenced on the Illumina MiSeq platform with a read depth of 7.5 million. The number of reads per gene per sample was determined using the Qiagen in-house bioinformatics pipeline. Genes were considered differentially expressed if the fold change reached statistical significance p-value <0.05. Count normalization and differential expression was performed using pre-determined reference genes and DEseq2 v1.32. All gene-expression data is available through Gene Expression Omnibus (GEO): GSE189245.

Reporter assays

2 x 10^4 PKN2^{WT} PSCs or 4 x 10^4 PKN2^{KO} PSCs were seeded in 500 μl PSC medium in 24-well plates. After 24 h, PSCs were co-transfected with a Renilla luciferase control plasmid (pRL, Promega) and a reporter plasmid encoding SMAD-Firefly luciferase (Dennler et al., 1998), or SRF-Firefly luciferase (pGL4.34 luc2P/SRF-RE/Hygro, Promega), or TEAD-Firefly luciferase (pGL3-4xGTIIC-49 (Mahoney et al., 2005)) in a ratio of 3:2 (Renilla plasmid :Firefly plasmid) and total DNA quantity amounting to 500ng/well. Transfection was performed with Lipofectamine LTX/Plus reagent (Invitrogen, 15388-100) at a final amount of 2 μl of Lipofectamine LTX and 0.5 μl of Plus reagent per 50 μl reaction in OptiMEM . Cells were starved overnight in 0.5% serum for 24 hrs and then stimulated with 5 ng/ml TGF-β1 (Peprotech) or vehicle for 24 h or 10% serum (50 μl FBS (Gibco)) for 3-6 h as indicated. Dual-Glo luciferase assay system (Promega) was used to detect Firefly- and Renilla-luciferase activity.

Nuclear localisation and cell size analysis

Images were analysed using CellProfiler (v3.1.9) (Carpenter et al., 2006) using a custom pipeline. Briefly, nuclear and cytoplasmic masks were created using the DAPI and phalloidin channels respectively and average YAP1 intensity calculated within each mask. Numbers of neighbours were determined by creating "centroids" of each nucleus and counting the number of cell points present within a 46 μm radius of each centroid; this radius was empirically assessed to best estimate number of cells in direct contact. For cell size, customized CellProfiler pipelines were used to determine cell area based on phalloidin staining.

qPCR analysis

PKN2 WT and KO PSCs were plated for 72 hours before being lysed with Trizol and RNA separated using chloroform extraction method as per manufactuer's instructions. 2 ml of Trizol was used to lyse 2 x 175 cm^2 TC flast of either PKN2 WT or KO PSCs, incubated for 5 mins at RT and then collected in 2 x 2 ml microcentrifuge tubes (1 ml each). 200 μl of chloroform was added to 1ml of trizol, incubated for 2-3 mins at RT, centrifuged at 12,000g for 15mins at 4°C. The aqueous phase was then separated into a fresh labelled tube and 500 ul of Isopropenol added for every 1 ml of trizol. This was incubated for 10 mins in the fridge and then centrifuged at 12000g at 4°C. RNA was then precipitated with Isopropanol and 70% ethanol and then dissolved in 30 μl of nuclease-free water. RNA was measured using a Nandrop (ThermoFisher Scientific). cDNA was prepared using Lunascript RT Supermix kit (Lunascript) as per manufacturer's instructions in a 20 μl reaction. This was then treated with Turbo DNase I (Life technologies) and diluted to 50 μl. qPCR on cDNA was performed using PowerUp SYBR Green Master Mix (Life technologies) with manufacturer's recommendations a Quantstudio 7 Flex (Applied biosystems). Oligonucleotides listed in Table S5.

Spheroid 3D co-cultures

Spheroid invasion assays were performed using a modified methylcellulose hanging drop protocol (Leung et al., 2015; Ware et al., 2016) optimised with Dr Edward Carter (BCI). Cancer cells and PSCs were aliquoted at a concentration of 2.2 x 10^4 cells/ml cancer cells ± 4.4 x 10^4 cells/ml PSCs, in 1 ml complete PSC medium. This was mixed with 1.2% methylcellulose (Sigma-Aldrich) in DMEM in a 4:1 ratio to give a final concentration of 0.24% methylcellulose and 1000 cells (333 cancer cells ± 667 PSCs) / 20 μl suspension. For each condition 20 μl hanging drops were incubated overnight at 37°C. The following day, 25 spheroids per condition were collected and centrifuged at 300g for 4 mins with the brake off and washed in PSC culture medium again. 40 μl Matrigel basement membrane matrix (Corning) was diluted 1:1 with PSC culture medium and added to the bottom of a 96-well clear bottom ultra-low attachment plates. For Immunofluorescence spheroids were plated in 96-well clear bottom black plates (Greiner, 655976-SIN). Gels were incubated at 37°C for 30 mins to set. After washing, spheroids were resuspended on ice in 300 μl Matrigel:medium. Spheroids were gently mixed by pipetting before aliquoting 50 μl of suspension per well (6 wells/ condition). After a final incubation of 30 mins at 37°C, 200 μl PSC medium was placed on top. Spheroids were incubated for 2-3 days and cell invasion was monitored daily by light microscopy

and epifluorescence and brightfield micrographs were taken on a light microscope. Z-stack immunofluorescence of spheroids was carried out using an LSM710 confocal microscope (Zeiss) and. All spheroids were analysed using ImageJ (Fiji) for area.

RNA sequencing and analysis

Tumours were extracted and a portion of each stored in RNA*later* (ThermoFisher Scientific, AM7020) at -20°C. To extract RNA, samples were macerated using the TissueLyzer II (QIAGEN) with 5 mm TissueLyzer beads (QIAGEN, 69989) in RLT buffer. RNA was prepared using the RNeasy mini kit (QIAGEN) as per manufacturer's instructions. RNA integrity was checked using the Agilent Fragment Analyzer (Agilent Technologies). The library was prepared using NEB Next Ultra II Directional RNA Library Prep Kit for Illumina using manufacturer's recommendations (NEB) and loaded on the Illumina NovaSeq6000 for paired-end sequencing. Adapter and Quality trimming, Genome Alignment and Annotation RNA-seq was performed in house. Raw FASTQ reads of length 151 bases were adapter and quality trimmed using trimmomatic before mapping to the mouse genome (mm10, Genome Reference Consortium GRCm38), to address 3′-end adapter contamination. Trimmed reads were aligned to the mouse genome in strand-specific mode using HISAT2 (Kim et al., 2015). A number of uniquely aligned reads ($q > 10$) to the exonic region of each gene were counted using HTSeq (Anders et al., 2015) based on Genome Reference Consortium Mouse Build 38 patch release 6. All gene-expression data is available through Gene Expression Omnibus (GEO): GSE189027.

Bioinformatics

Differential expression analysis was conducted using the Bioconductor R packages edgeR (Robinson et al., 2010) and DESeq2 (Love et al., 2014). Gene Set Enrichment Analysis (GSEA) was performed using the Broad Institute GSEA software (Subramanian et al., 2005) and R package fgsea (DOI: https://doi.org/10.1101/060012) using gene sets curated from the Molecular Signatures Database (MSigDB v6.2). Survival and other bioinformatic analyses were conducted using customised R scripts. All code available through Zenodo. Single-cell RNA seq data was obtained from: (Biffi et al., 2019) and (Peng et al., 2019) to probe for PKN2 expression across different tumour cell types. The filtered unique molecular identifier (UMI) count matrix was processed using the R package Seurat (v4.0.4). Using the top 18 principal components (PCs), the main cell clusters were identified using the FindClusters function of Seurat and visualised using 2D uniform manifold approximation and projection (UMAP).

siRNA transfection

5×10^4 PSCs were seeded in 6-well plates. 24 h later cells the medium was changed and cells were transfected with 20 nM pooled control or PKN2-targeting siRNAs from SMARTpool siGENOME siRNAs (Dharmacon, GE Healthcare) with Lipofectamine 2000 (Invitrogen) as per the manufacturer's instructions. Transfection complexes were prepared as follows: 5 μl Lipofectamine 2000 reagent was diluted in 100 μl Opti-MEM. In a separate tube, 2 μl 20 μM pooled siRNA was diluted in 100 μl Opti-MEM. The siRNA/Opti-MEM solution was then added to the lipofectamine solution. The resulting transfection complex solution was mixed, incubated for 5 mins at RT and 200 μl transfection mix added to each well. 48 h after transfection PSCs were embedded in spheroids with cancer cells as described in "Spheroid 3D co-cultures" and imaged after 2-3 days by light microscopy and epifluorescence. Invasion was quantified from brightfield images by measuring the area of invading cells protruding from the body of each spheroid relative to the area of the entire spheroid in ImageJ. Z-stack immunofluorescence images of sleected spheroids were also imaged 3 days after seeding by live cell confocal microscopy using the LSM 710 confocal microscope (Zeiss) with cells maintained in the imaging chamber at 37°C and 5% CO_2. siRNA sequences are provided in Table S6.

Immunohistochemistry

Paraffin-embedded sections of tissue were dewaxed and rehydrated by heating slides at 60°C for 1 hr and then incubating in xylene (5 mins), 100% ethanol (10 mins), 80% ethanol (2 mins), 70% ethanol (2mins), 50% ethanol (2 mins), distilled water (2 mins). They were then treated with 3% hydrogen peroxide for 10 mins and antigen retrieval was performed by incubating in boiling hot 0.01 M tri-sodium citrate pH 6 buffer for 10 mins with continued boiling. Slides were then incubated in 0.1% PBS-Tween for 5mins, and incubated in blocking buffer (0.02% Fish Skin gelatin with horse serum in PBS-T). Primary and secondary antibodies as listed in the key resources table were diluted in blocking buffer and then used to bind indicated markers. Antigen detection using Universal Vectastain ABC Kit (Vector Laboratories, PK-6200) and DAB (Dako, K3468) used as per manufacturer's instructions. MI stains were done on consecutive sections of paraffin embedded blocks and images taken using Pannoramic Scanner (3D Histech). MI overlay was processed using ImageJ software. Quantitative analysis was done using various measurement applications on Visiopharm (Version 2019.07.3.7092) and QuPath (Bankhead et al., 2017).

In vivo tumour experiment

All mice received the same tamoxifen injections (5 injections at 2 mg/21 g weight) to induce Cre recombinase activity, were rested for a week and then orthotopically injected with PDAC cell line TB32048. Animals were administered with analgesic (60 ml of 0.3 mg/ml buprenol) and anaesthetic (Isofluorane) before having orthotopic injections of 1000 TB32048 cells in a 1:1 solution of DMEM and Matrigel into the pancreas using an insulin syringe. Tumour endpoint measurements did not exceed $1.4 cm^3$ as per the project licence. This was assessed by MRI and mice were culled for tumour and organ harvest. At harvest, tumours were weighed, photographed and then measured for widest length and width using Vernier calipers. Volumetric measurements were made using length x $(width)^2/2$.

Tumours were then collected in bijous containing 4% PFA, transferred to 70% ethanol the following day and then paraffinized and sectioned at 4 μm for immunohistochemical staining.

QUANTIFICATION AND STATISTICAL ANALYSIS

Unless otherwise stated, quantitative results are presented as mean ± standard deviation (SD). Where appropriate, statistical analysis utilised two-tailed unpaired t-test, one-way ANOVA or two-way ANOVA in Prism 8 (GraphPad Software). For ANOVA, Tukey's post-hoc test was used for data with more than one independent variable and Sidak's test was used for multiple pairwise comparisons when considering a single variable; repeated unpaired t-tests with Bonferroni correction was performed to account for variance within the specified comparisons. The statistical analyses used in each experiment, if different to these, is detailed in the corresponding figure legends. In *in vivo* experiments, n refers to the number of animals. In *in vitro* experiments, n refers to the number of biological replicates. *p<0.05, **p<0.01, ***p<0.001 and ****p<0.0001 were considered significant.

Cell Reports, Volume *38*

Supplemental information

Disruption of pancreatic stellate cell myofibroblast phenotype promotes pancreatic tumor invasion

Elizabeth R. Murray, Shinelle Menezes, Jack C. Henry, Josie L. Williams, Lorena Alba-Castellón, Priththivika Baskaran, Ivan Quétier, Ami Desai, Jacqueline J.T. Marshall, Ian Rosewell, Marianthi Tatari, Vinothini Rajeeve, Faraz Khan, Jun Wang, Panoraia Kotantaki, Eleanor J. Tyler, Namrata Singh, Claire S. Reader, Edward P. Carter, Kairbaan Hodivala-Dilke, Richard P. Grose, Hemant M. Kocher, Nuria Gavara, Oliver Pearce, Pedro Cutillas, John F. Marshall, and Angus J.M. Cameron

Figure S1

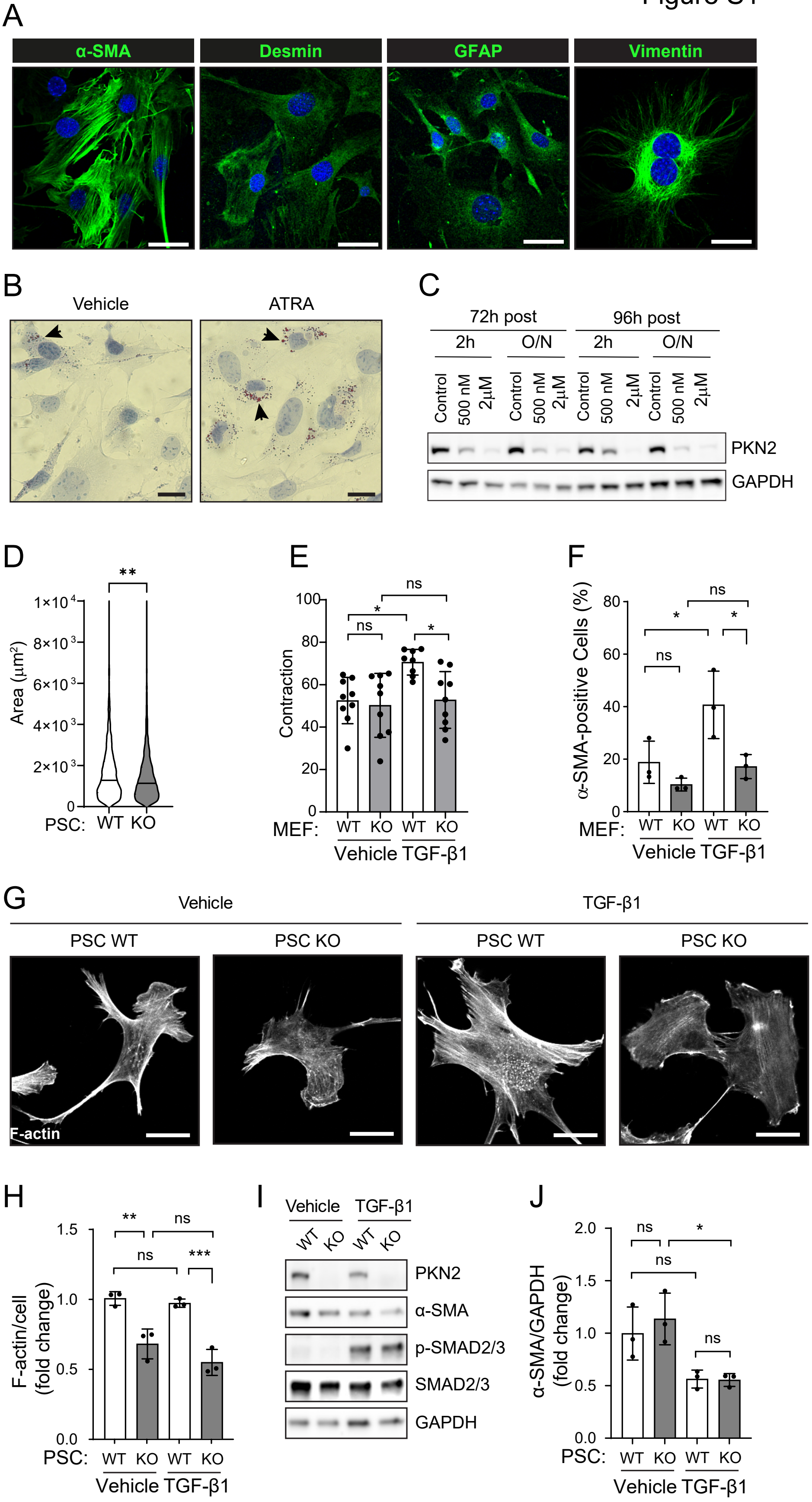

Supplemental Figure 1 (Related to Figure 1). Characterisation of inducible PKN2^{KO} PSCs and MEFs. (A) Immunofluorescence images of α-SMA, Desmin, GFAP and Vimentin staining of PSCs plated on glass coverslips. Scale bar=50 µm. (B) Oil Red O staining of PSCs plated on glass coverslips and treated with vehicle or ATRA daily for 4 days. Scale bar=25 µm. (C) Western Blot analysis of PKN2 deletion *in vitro* in PSCs for indicated time points of incubation and concentration of 4-hydroxy tamoxifen. (D) Quantification of area of WT and PKN2^{KO} PSCs as determined from phalloidin stains imaged by confocal microscopy. (E) Quantification of collagen gel contraction of WT and PKN2^{KO} MEFs treated with 5 ng/ml TGF-β1 or vehicle for 72 h expressed using the formula (1- ratio of gel size/well size)*100. Data are relative to WT MEFs treated with vehicle (n=3, two-way ANOVA with Tukey's multiple comparisons test). (F) Percentage of α-SMA-positive cells in WT or PKN2KO MEFs treated with vehicle or 5 ng/ml TGF-β1 for 72 h (n=3, two-way ANOVA with Tukey's multiple comparisons test). (G) F-actin staining in WT and PKN2^{KO} PSCs treated with vehicle or 5 ng/ml TGF-β1 for 72 h. Scale bar = 25 µm. (H) Number of F-actin fibres per cell treated with vehicle or 5 ng/ml TGF-β1 for 72 h relative to WT PSCs treated with vehicle, quantified by MATLAB algorithm (n=3, >30 cells/condition/experiment). (I) Total α-SMA expression in WT and PKN2^{KO} PSCs treated with vehicle or 5 ng/ml TGF-β1 for 72 h (representative of n=3). (J) Quantification of total α-SMA/GAPDH in WT and KO PSCs treated with vehicle or 5 ng/ml TGF-β1 for 72 h (n=3, two-way ANOVA with Tukey's multiple comparisons test); *p<0.05**p<0.01, ***p<0.001.

Figure S2

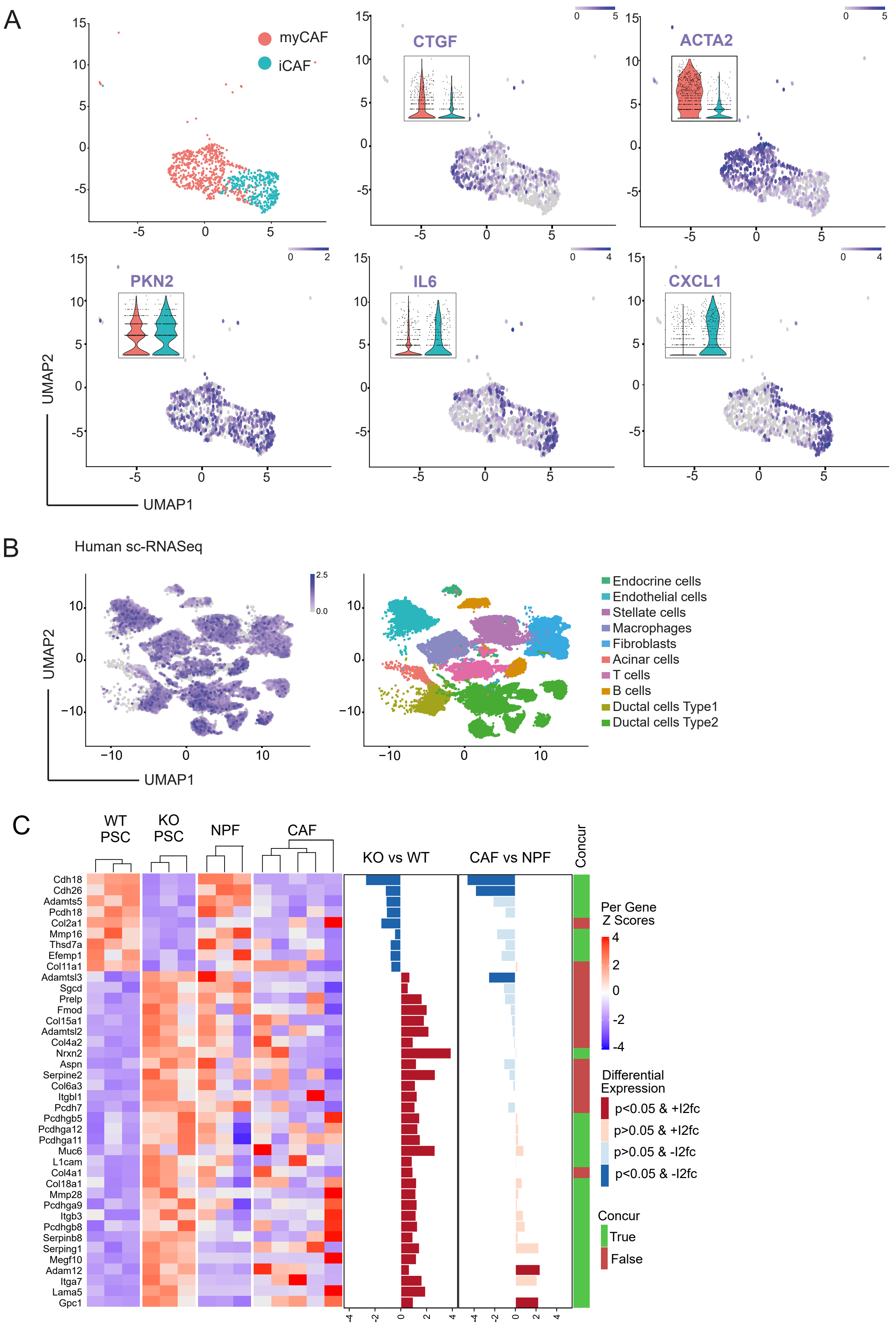

Supplemental Figure 2 (Related to Figure 2). PKN2-deleted PSCs show a distinct matrisome expression profile to normal pancreatic fibroblasts. (A) UMAP plots of single cell RNA-seq data from Biffi et al, 2019 resolving fibroblasts into iCAFs and myCAFs based on iCAF markers Il6, Lif and Cxcl1 and myCAF markers Acta2, Ctgf.; expression level of indicated genes among single cells overlayed in purple on UMAPs. Insets show violin plots of expression levels of indicated genes in iCAFs (pink) and myCAFs (blue). (B) UMAP analyses of single cell RNA seq data from Peng et al, 2019 of various tumour microenvironment cell populations overlayed with their expression level of PKN2 (purple). (C) The expression of ECM panel transcripts was compared between our WT and PKN2KO PSCs and mouse orthotopic pancreatic tumour CAF expression data from Djurek et al, using the panel of DE ECM genes. Concurrence of changes between the two datasets is indicated in the right-hand side bar (concur).

Figure S3

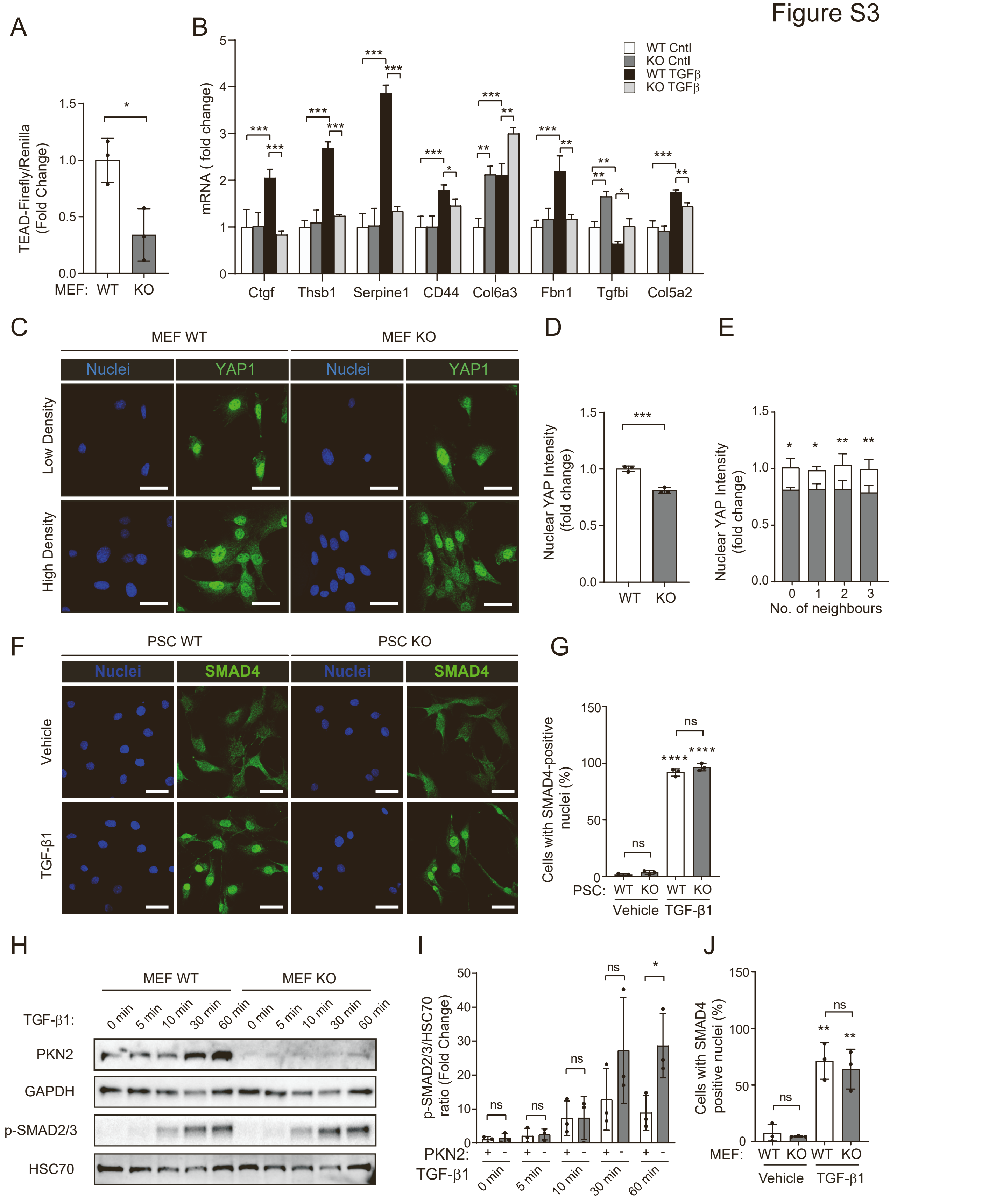

Supplemental Figure 3 (Related to Figure 3). Characterisation of YAP and TGF-β1 signalling in PKN2^{KO} MEFs and PSCs. (A) Normalised expression of TEAD Firefly luciferase reporter relative to the expression of a Renilla luciferase control reporter in WT and KO MEFs grown in 10% serum (unpaired t-test from triplicate transfections; n=2). (B) mRNA expression analysis of indicated genes from targeted RNA-seq data of WT and PKN2^{KO} PSCs treated with either control or TGFβ1 and expressed relative to WT control (n=4; ratio paired t-test). (C) Representative confocal microscopy images of YAP1 localisation (green) in WT and PKN2^{KO} MEFs plated at high or low density on glass coverslips for 48hrs; (minimum of 200 cells/ condition, n=3; scale bar=50 μm). (D) Quantification by Python algorithm of YAP nuclear intensity of all WT and PKN2^{KO} MEFs plated relative to WT (n=3, unpaired t-test). (E) Quantification by Python CellProfiler algorithm of YAP nuclear intensity grouped by number of cell neighbours in WT (white) and PKN2^{KO} (grey) MEFs relative to WT MEFs with zero neighbours.; n=3, two-way ANOVA with Sidak's multiple comparisons test. (F, G) Representative images (F) and quantification (G) (percentage of positive cells) of nuclear SMAD4 (green) in serum-starved or PSCs treated with 5 ng/ml TGF-β1 for 2h (Scale bar = 50 μm, n=3 , two-way ANOVA with Tukey's multiple comparisons test. (H) p-SMAD2/3 induction in WT and PKN2^{KO} MEFs treated with 5 ng/ml TGF-β1 for 0, 5, 10, 30 or 60 mins (representative of n=3). (I) Quantification of pSMAD2/3 induction relative to HSC70 (expressed relative to untreated WT MEFs); n=3, two-way ANOVA with Sidak's multiple comparison's test. (J) Percentage of WT and PKN2^{KO} MEFs with SMAD4-positive nuclei when serum-starved and treated with vehicle or 5 ng/ml TGF-β1 for 2 h; n=3, two-way ANOVA with Tukey's multiple comparisons test. *p<0.05, **p<0.01, ***p<0.001.

Figure S4

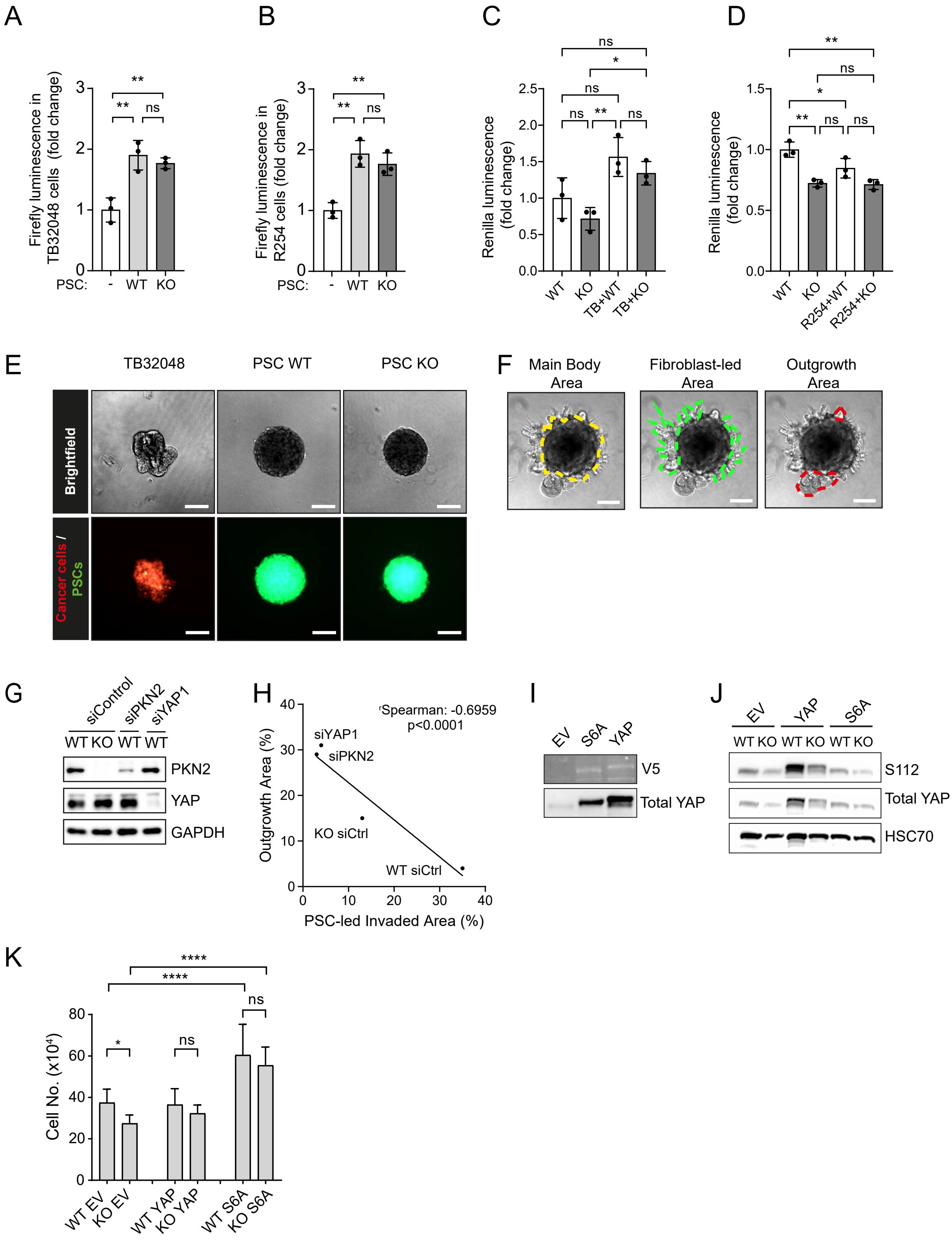

Supplemental Figure 4 (Related to Figure 4). Impact of PKN2 loss on PSC induced growth and invasion. (A, B) Firefly luminescence of TB32048 cells or R254 cells cultured alone or with WT or PKN2^{KO} PSCs in 0.5% serum for 3 days expressed as fold change relative to cells cultured alone (n=3, one-way ANOVA with Tukey's multiple comparisons test). (C,D) Renilla luminescence of WT or PKN2^{KO} PSCs cultured with (C) TB32048 cells or (D) R254 cells in 0.5% serum for 3 days expressed as fold change to WT PSCs cultured alone (n=3, one-way ANOVA with Tukey's multiple comparisons test). (E) Brightfield (top) and epifluorescence images (bottom) of spheroids containing H2B-RFP TB32048 PDAC cells (red), or H2B-GFP WT or PKN2^{KO} PSCs alone (green) embedded in Matrigel matrix for 3 days (n>5). (F) Overlays illustrating definition of spheroid body area (yellow), fibroblast-led invaded area (green) and cancer cell outgrowth area (red) based on cell morphology under brightfield microscope. (G) Western blot analysis of PKN2 and YAP expression in PSCs treated with siRNA against PKN2 or YAP. (H) Correlation of the percentages of PSC-led invaded area and cancer cell outgrowth area relative to total area of each spheroid. (I) Western blot of V5 tag and total YAP in PSCs overexpressing V5-tagged WT YAP, S6A YAP, or empty vector control (EV) as detected by fluorescence (V5 tag) and chemiluminescence (YAP). (J) p-YAP S112, total YAP and HSC70 western blot analysis of PKN2 WT or KO PSCs with empty vector (EV), WT YAP or S6A YAP (S6A). (K) Hemocytometer cell count of EV , WT YAP or YAP S6A PSCs (PKN2 WT and KO) grown in 6-well plates for 8 days in triplicate (n=3, 2-way ANOVA with Tukey's multiple comparisons test).

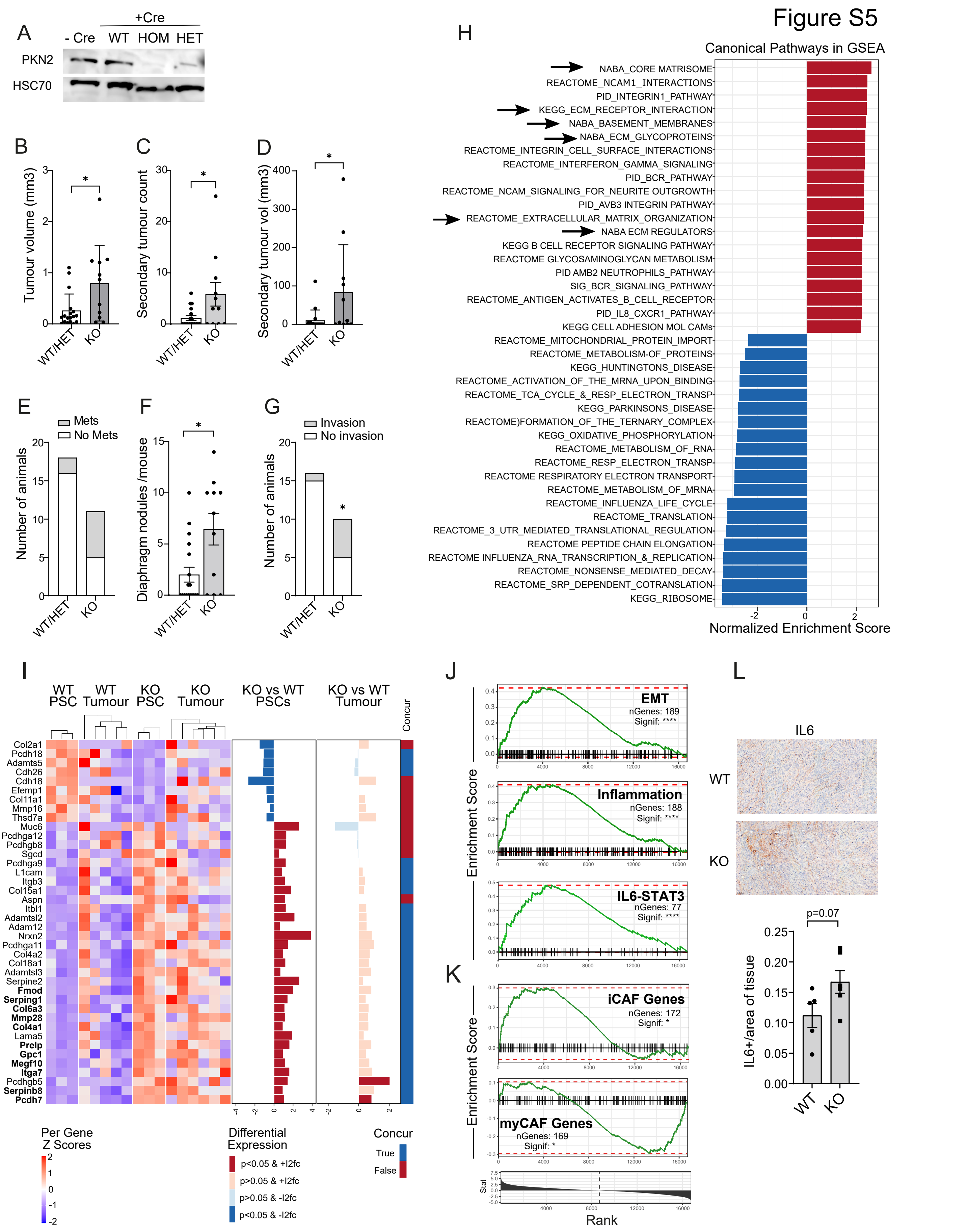

Supplemental Figure 5 (Related to Figure 5). Deletion of stromal PKN2 results in an enhanced iCAF phenotype in tumours in vivo. (A) Western blot of PKN2 and HSC70 in pancreas from Cre negative mice and Cre+ PKN2WT, PKN2KO and PKN2HET littermates. (B-D) Quantification of primary tumour volume (B) secondary tumour count per mouse and secondary tumour volume in controls (PKN2WT and PKN2HET) versus PKN2KO (n=8-11, Student's t-test, *p<0.05). (E-G) Quantification of number of animals with (grey) and without (white) peritoneal metastasis (Fisher's exact test), the number of diaphragmatic nodules per mouse (Student's t-test) (F) and the number of animals with (grey) and without (white) sites of invasion observed in cross-sections of the primary tumour (G) (n=8-11, *p<0.05, Fisher's exact test). (H) GSEA of orthotopic tumours showing the 20 most highly upregulated (red) and downregulated (blue) canonical pathways in PKN2KO tumours compared with WT tumours. (I) Comparison of transcriptomic expression data from WT and PKN2KO PSCs, with RNA-Seq data from tumours grown in WT and PKN2KO mice for DE ECM genes identified in Figure 2C. (J) GSEA enrichment plots for EMT, inflammation and IL6-Jak-STAT3 signalling in orthotopic tumours grown in PKN2KO mice as compared to WT mice (NES >2.0). (K) GSEA enrichment plots of genes upregulated in either iCAFs (bottom panel; NES = 1.41) or myCAFs (top panel; NES= -1.38) in PKN2KO tumours over PKN2WT tumours. (L) Representative images and quantification of IL6 staining in tumours grown in WT and PKN2KO mice.

Figure S6

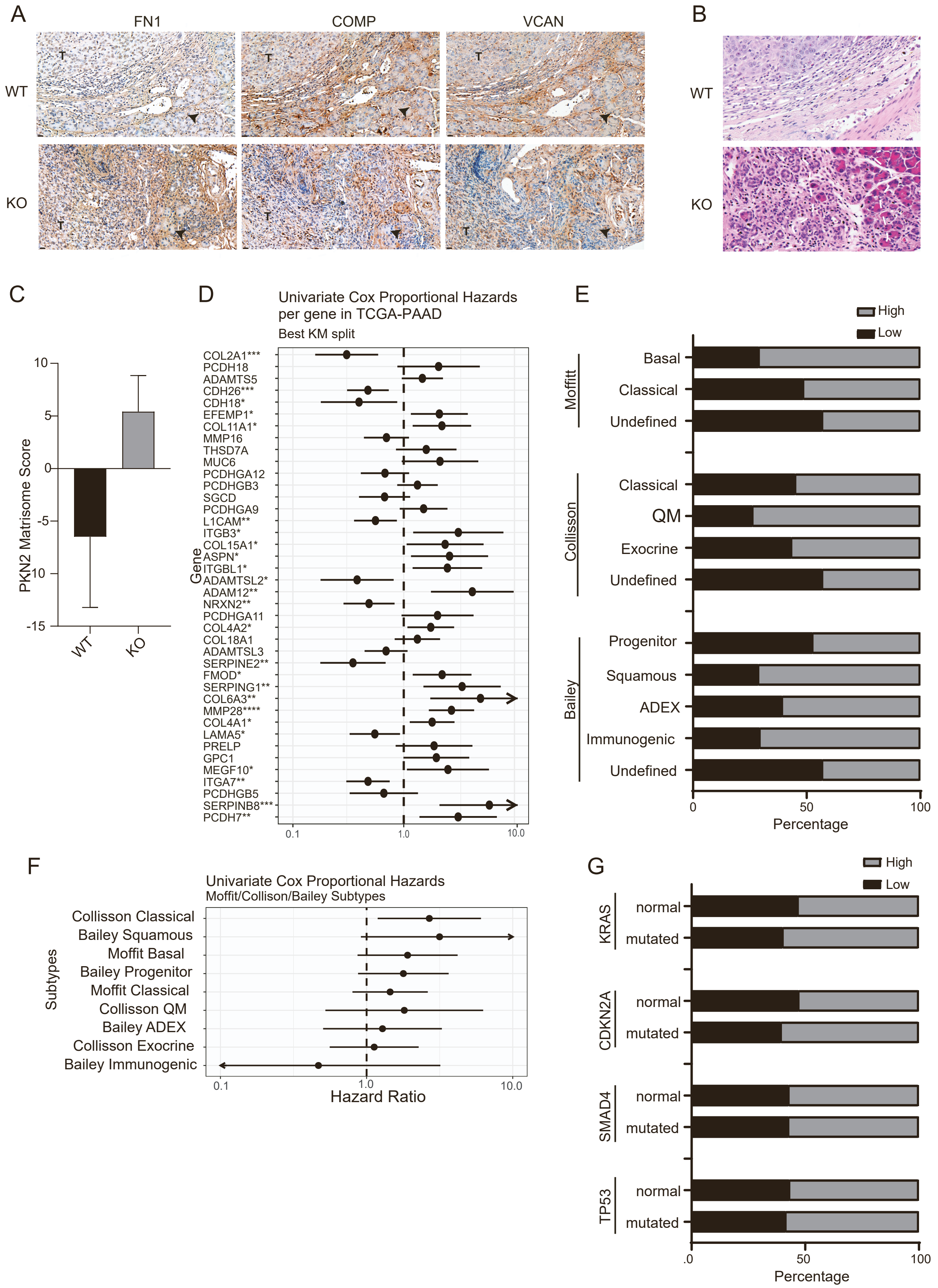

Supplemental Figure 6 (Related to Figure 6). PKN2^{KO} matrisome score identifies PDAC patients with poor prognosis. (A) IHC staining for ECM components FN1, COMP and VCAN, at the tumour edge or invasive front of representative WT (top panels) or PKN2^{KO} (bottom panels) tumours, performed on consecutive sections. (B) H&E of WT (top) and KO (bottom) tumours on sections cut approximately 50μm before those used for MI staining. (C) PKN2^{KO} matrisome score in orthotopic tumours from PKN2 WT and KO mice; n=5-6. (D) Cox proportional hazards test per gene from the PKN2^{KO} matrisome score in TCGA-PAAD data. (E) Percentage of TCGA-PAAD samples within each subtype of the Moffitt, Collison and Bailey datasets exhibiting high (grey bars) and low (black bars) PKN2^{KO} matrisome score. (F) Univariate Cox proportional Hazards test comparing high and low PKN2^{KO} matrisome score within each tumour subtype from Moffitt, Collison and Bailey datasets. (G) Percentage of samples with high or low PKN2^{KO} matrisome score that are WT or mutated for key PDAC driver genes.

SUPPLEMENTAL TABLES

Table S3 (Related to Figure 6): PKN2^{KO} ECM signature

Mouse_ensembl_gene_id	Mgi_symbol	Description	Human_ensembl_gene_id	Hgnc_symbol
ENSMUSG00000020682	Mmp28	matrix metallopeptidase 28 (epilysin) [Source:MGI Symbol;Acc:MGI:2153062]	ENSG00000271447	MMP28
ENSMUSG00000023224	Serping1	serine (or cysteine) peptidase inhibitor, clade G, member 1 [Source:MGI Symbol;Acc:MGI:894696]	ENSG00000149131	SERPING1
ENSMUSG00000024593	Megf10	multiple EGF-like-domains 10 [Source:MGI Symbol;Acc:MGI:2685177]	ENSG00000145794	MEGF10
ENSMUSG00000025348	Itga7	integrin alpha 7 [Source:MGI Symbol;Acc:MGI:102700]	ENSG00000135424	ITGA7
ENSMUSG00000026315	Serpinb8	serine (or cysteine) peptidase inhibitor, clade B, member 8 [Source:MGI Symbol;Acc:MGI:894657]	ENSG00000166401	SERPINB8
ENSMUSG00000029108	Pcdh7	protocadherin 7 [Source:MGI Symbol;Acc:MGI:1860487]	ENSG00000169851	PCDH7
ENSMUSG00000031502	Col4a1	collagen, type IV, alpha 1 [Source:MGI Symbol;Acc:MGI:88454]	ENSG00000187498	COL4A1
ENSMUSG00000034220	Gpc1	glypican 1 [Source:MGI Symbol;Acc:MGI:1194891]	ENSG00000063660	GPC1
ENSMUSG00000041559	Fmod	fibromodulin [Source:MGI Symbol;Acc:MGI:1328364]	ENSG00000122176	FMOD
ENSMUSG00000041577	Prelp	proline arginine-rich end leucine-rich repeat [Source:MGI Symbol;Acc:MGI:2151110]	ENSG00000188783	PRELP
ENSMUSG00000048126	Col6a3	collagen, type VI, alpha 3 [Source:MGI Symbol;Acc:MGI:88461]	ENSG00000163359	COL6A3

Table S5 (Related to STAR Methods- qPCR analysis): Oligonucleotides used in qPCR.

Resource/ Reagent		Source	Identifier
Name	Sequence		
IL-6-forward	5’-AGCCAGAGTCCTTCAGAGAGA-3’	This paper; Merck	N/A
IL-6-forward	5’-GGAGAGCATTGGAAATTGGGG-3’	This paper; Merck	N/A
Lif-Forward	5’-CGGCAACCTCATGAACCAGA-3’	This paper; Merck	N/A
Lif- Reverse	5’-GGAAACGGCTCCCCTTGA-3	This paper; Merck	N/A
Cxcl1-Forward	5’-CCAGAGCTTGAAGGTGTTG-3’	This paper; Merck	N/A
Cxcl1-Reverse	5’-AGCTTCAGGGTCAAGGCAAG-3’	This paper; Merck	N/A
Pparg- Forward	5’-GGGGATGTCTCACAATGCCA-3’	This paper; Merck	N/A
Pparg- Reverse	5’-TGGGTTCAGCTGGTCGATATC-3’	This paper; Merck	N/A
Plin2- Forward	5’-TGCCCATCATCCAGAAGCTG-3’	This paper; Merck	N/A
Plin2- Reverse	5’-AACAATCTCGGACGTTGGCT-3’	This paper; Merck	N/A
Diaph3- Forward	5’-GAGAAGCGACCCAAGTTGCAT-3’	This paper; Merck	N/A
Diaph3-Reverse	5’-GAAGGGGAGGTCTCTCTTTCTT-3’	This paper; Merck	N/A
Flna- Forward	5’-GTACCGTGTCCGGGCTGTGC-3’	This paper; Merck	N/A
Flna- Reverse	5’- ACATGCTCGCCACCGAAGCG-3’	This paper; Merck	N/A
Anln-Forward	5’-TGGGGCTGAGCAGATGGTCG-3’	This paper; Merck	N/A

Anln- Reverse	5’-TCCGGGACTGGCCATAACTGAAGA-3’	This paper; Merck	N/A
Ankrd1-Forward	5’-AAACGGACGGCACTCCACCG-3’	This paper; Merck	N/A
Ankrd1- Reverse	5’-CGCTGTGCTGAGAAGCTTGTCTCT-3’	This paper; Merck	N/A
Amotl2- Forward	5’-AACCGCCACCTGGCAAGCAA-3’	This paper; Merck	N/A
Amotl2- Reverse	5’-GGTCCTCGATGGCACCACGC-3’	This paper; Merck	N/A
Sdpr- Forward	5’-GCCCAGCAGGTGCGCTATGA-3’	This paper; Merck	N/A
Sdpr-Reverse	5’-CGGGGTGGCTTCCACGAGGT-3’	This paper; Merck	N/A
18s-Forward	5’-CACGGGAAACCTCACCCGGC-3’	This paper; Merck	N/A
18s- Reverse	5’-AACGGCCATGCACCACCACC-3’	This paper; Merck	N/A

Table S6 (Related to STAR Methods- siRNA transfection): Oligonucleotides used in siRNA experiment.

Reagent		Source	Identifier	
Reagent	Sequence		Pool Identifier	Identifier
siCtrl #1	UAGCGACUAAACACAUCAA	Dharmacon		D-001210-01-20
siPKN2 #1	GGAAAGUUACAACAGAUAA	Dharmacon	M-065210-02	D-065210-01
siPKN2 #2	UAUCCAAGGUUCUUAUCUA	Dharmacon	M-065210-02	D-065210-02
siPKN2 #3	CGACAUCAAGGAUCGAAUA	Dharmacon	M-065210-02	D-065210-03
siPKN2 #17	AGACUAGUGAGACGAGCUA	Dharmacon	M-065210-02	D-065210-17
siYAP1	GGAGAAGUUUACUACAUAA	Dharmacon	M-046247-01	D-046247-01
siYAP1	CCACCAAGCUAGAUAAAGA	Dharmacon	M-046247-01	D-046247-02
siYAP1	GAGAUGCAAUGAACAUAGA	Dharmacon	M-046247-01	D-046247-03
siYAP1	CAAUAGUUCCGAUCCCUUU	Dharmacon	M-046247-01	D-046247-04
